# Novel Tyrosine Kinase Inhibitors to Target Chronic Myeloid Leukemia

**DOI:** 10.3390/molecules27103220

**Published:** 2022-05-18

**Authors:** Valeria Ciaffaglione, Valeria Consoli, Sebastiano Intagliata, Agostino Marrazzo, Giuseppe Romeo, Valeria Pittalà, Khaled Greish, Luca Vanella, Giuseppe Floresta, Antonio Rescifina, Loredana Salerno, Valeria Sorrenti

**Affiliations:** 1 Department of Drug and Health Sciences, University of Catania, Viale A. Doria 6, 95125 Catania, Italy; valeria_consoli@yahoo.it (V.C.); s.intagliata@unict.it (S.I.); marrazzo@unict.it (A.M.); gromeo@unict.it (G.R.); vpittala@unict.it (V.P.); lvanella@unict.it (L.V.); arescifina@unict.it (A.R.); sorrenti@unict.it (V.S.); 2 Department of Molecular Medicine, College of Medicine and Medical Sciences, Princess Al Jawhara Centre for Molecular Medicine, Arabian Gulf University, Manama 329, Bahrain; khaledfg@agu.edu.bh; 3 Department of Analytical, Environmental and Forensic Sciences, King’s College London, London SE1 9NH, UK; giuseppe.floresta@unict.it

**Keywords:** chronic myeloid leukemia, tyrosine kinase inhibitors, nilotinib derivatives, structure–activity relationship studies, docking studies, molecular dynamic simulation, heme oxygenase

## Abstract

This paper reports on a novel series of tyrosine kinase inhibitors (TKIs) potentially useful for the treatment of chronic myeloid leukemia (CML). The newly designed and synthesized compounds are structurally related to nilotinib (NIL), a second-generation oral TKI, and to a series of imatinib (IM)-based TKIs, previously reported by our research group, these latter characterized by a hybrid structure between TKIs and heme oxygenase-1 (HO-1) inhibitors. The enzyme HO-1 was selected as an additional target since it is overexpressed in many cases of drug resistance, including CML. The new derivatives **1a**–**j** correctly tackle the chimeric protein BCR-ABL. Therefore, the inhibition of TK was comparable to or higher than NIL and IM for many novel compounds, while most of the new analogs showed only moderate potency against HO-1. Molecular docking studies revealed insights into the binding mode with BCR-ABL and HO-1, providing a structural explanation for the differential activity. Cytotoxicity on K562 CML cells, both NIL-sensitive and -resistant, was evaluated. Notably, some new compounds strongly reduced the viability of K562 sensitive cells.

## 1. Introduction

Chronic myeloid leukemia (CML) belongs to blood malignancies and accounts for about 15% of adult leukemia. CML is caused by a chromosomal translocation leading to the Philadelphia chromosome (Ph) that generates the BCR-ABL oncogene, which produces, in turn, the chimeric oncoprotein BCR-ABL with high tyrosine kinase (TK) activity [1]. The abnormal phosphorylation caused by BCR-ABL over-activity results in increased myeloid stem cell proliferation with consequent initiation, maintenance, and progression of CML. Imatinib (IM), the first tyrosine kinase inhibitor (TKI) approved by the FDA in 2001, represents a milestone in CML therapy [2]. It binds to the catalytic domain of BCR-ABL protein, preventing the binding of ATP, the phosphorylation of tyrosine residues on various substrates, and, finally, cancer cell proliferation. Although IM radically changed the lifespan of CML patients, about one-third of them treated with IM show no optimal outcomes due to the onset of drug resistance [3]. Thereby, new-generation TKIs have been developed, and, among them, nilotinib (NIL), dasatinib, bosutinib, and ponatinib are already approved [4]. The most common cause of resistance is point mutations in the BCR-ABL gene. The second-generation TKIs dasatinib, NIL, and bosutinib can overcome a broad spectrum of mutations resistant to IM, resulting more potent than IM itself. On the other hand, they are not effective against T315I mutation, which is treatable only with the third-generation TKI ponatinib [5]. Although new-generation TKIs improved the outcome of CML, therapy is potentially lifelong, and severe side effects are associated with pharmacological treatment [6]. In addition, a high percentage of CML patients remain resistant to all kinds of TKIs, justifying further efforts in the development of new drugs. For instance, Wang Ji-shi et al. reported that possible mechanisms involved in NIL-resistant K562-R cells are associated with BCR-ABL, heme oxygenase (HO) system, and multidrug resistance 1 (MDR1) overexpression along with down-regulation of caspase-3 mRNA and protein levels [7,8]. In vitro evidence also suggests that ATP-binding cassette subfamily C member 6 (ABCC6) overexpression may play a role in the development of resistance to both NIL and dasatinib, although the contribution to resistance in the clinical setting remains to be elucidated [9]. HO is an enzyme that exerts a cytoprotective physiological role by degrading the pro-oxidant heme into free iron and simultaneous production of anti-inflammatory and antioxidant molecules, such as carbon monoxide, biliverdin, and bilirubin [10]. Two main isoforms, the first inducible (HO-1) and the second constitutive (HO-2), are active in mammalian cells and may serve as therapeutic targets [11,12]. Many factors induce HO-1 synthesis and activity, including heat, the same substrate heme, heavy metals, reactive oxygen species (ROS), hypoxia, nitric oxide (NO), ultraviolet radiations, and xenobiotic, generally leading to cytoprotection [13,14]. However, HO-1 overexpression may be a double-edged sword, especially in cancer. Indeed, aberrant HO-1 activity in cancer cells contributes to their survival, growth, progression, metastasis, and development of tolerance to chemotherapeutic, radiation, and photodynamic therapies [15,16]. Therefore, HO-1 inhibition may be considered for more effective anticancer treatments, specifically to reduce drug resistance [17,18]. To this end, so far, we have developed a large library of selective HO-1 inhibitors belonging to the class of imidazole-based derivatives [11,18,19,20,21,22].

**Figure 1:**
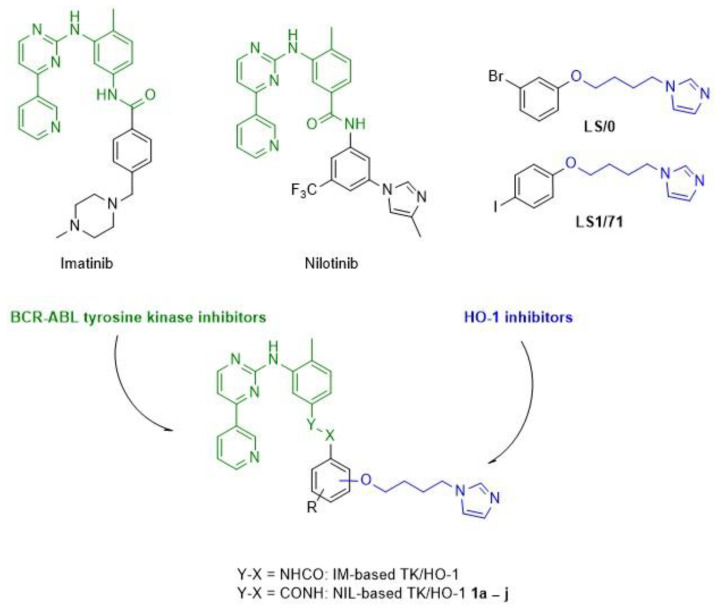
Chemical structure of IM, NIL, and HO-1 inhibitors **LS/0** and **LS1/71** and general structures of the IM/NIL-based derivatives.

**Scheme 1:**
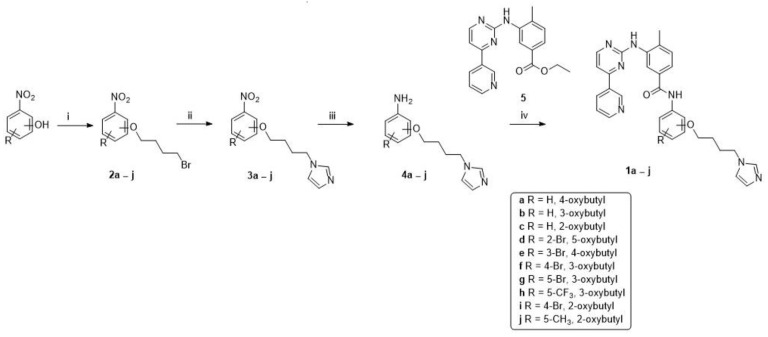
Reagents and conditions: (i) 1,4-dibromobutane, acetone, K_2_CO_3_, MW 90 °C, 45′; (ii) imidazole, NaH, THF, rt, 24 h; (iii) iron powder, NH_4_Cl, CH_3_OH/H_2_O, 65 ℃, 3 h; (iv) intermediate **5**, dry THF, *t*-BuOK, rt, under nitrogen, overnight.

Many studies reported in the literature demonstrate that BCR-ABL affects the expression of HO-1 in CML cells [23,24,25]. It was found that HO-1 levels are increased in IM-resistant cells in comparison with IM-sensitive ones [26]. Interestingly, we recently demonstrated that silencing HO-1 with siRNA, as well as inhibition of HO-1 enzymatic activity by imidazole-based inhibitors, restored the sensitivity of IM-resistant cells [17,26]. It has also been reported that compounds able to inhibit HO-1 protein enhanced not only IM but also NIL effects in decreasing leukemia cells’ survival [27]. These data show that HO-1 is a promising novel target in BCR-ABL TKI-resistant CML. In our previous study, we developed TKI/HO-1 hybrid compounds that inhibit both the enzymatic activity of HO-1 and BCR-ABL TK in K562 IM-resistant cells (Figure 1) [28]. These multitarget compounds were obtained by hybridizing the structure of IM and aryloxybutylimidazole HO-1 inhibitors **LS/0** and **LS1/71** (Figure 1) in order to obtain the same effect achieved with a combination of IM and HO-1 inhibitors. This strategy follows a well-established medicinal chemistry approach to avoid the potential side effects of a negative interaction between two different drugs administered simultaneously [29,30]. Interestingly, we found that many hybrids were able to inhibit both targets and reduced the viability of CML IM-resistant cells by apoptosis and by an increase of the ROS levels. These outcomes confirmed that a single molecular entity may function as the combination of two drugs and that HO-1 inhibition may overcome IM-resistance in CML [28]. To continue a research project that includes design, synthesis, molecular modeling, and in vitro anticancer evaluation of new hybrid compounds obtained by fusing the structure of TKIs and HO-1 inhibitors, in this work, we designed and synthesized a new series of compounds **1a**–**j** (Figure 1, Scheme 1), in which the IM-like moiety present in the previous series of TKIs is replaced by a NIL-like portion. Therefore, the main structural modification is the inversion of the central amide group (Figure 1). NIL being a more potent TKI than IM, this new series of derivatives should provide higher cytotoxicity in CML cell lines. The new compounds also bear an aryloxybutylimidazole portion, which shows the key chemical features required for HO-1 inhibition (an imidazole ring and a hydrophobic portion, linked via an oxybutyl chain) and which should be well tolerated inside the BCR-ABL binding pocket. To gain insight into structure–activity relationship (SAR) studies, the newly synthesized derivatives carry various substituents at the benzamide ring with the aryloxybutylimidazole moiety placed at different positions. New compounds were tested to evaluate their capacity of inhibiting BCR-ABL TK and HO-1 activities and their cytotoxicity in both NIL-resistant and -sensitive CML K562 cell lines. Differently from IM resistance, few studies regarding the involvement of HO-1 overexpression in NIL resistance have been described; therefore a K562 NIL-resistant line was developed, and HO-1 level was determined by Western blot analysis. Docking studies were also performed to gain information regarding the binding mode at both targets. 

## 2. Results and Discussions

### 2.1. Chemistry

Reaction conditions for preparing the final compounds **1a**–**j** are described in Scheme 1. Bromobutoxy nitrobenzenes **2a**–**j** were synthesized starting from the commercially available nitro phenols through etherification with 1,4-dibromobutane in acetone and in the presence of K_2_CO_3_. Imidazolylbutoxy nitro analogs **3a**–**j** were obtained through the nucleophilic displacement of intermediates **2a**–**j** with imidazole in tetrahydrofuran (THF) using NaH. The reduction of the nitro group to give aniline intermediates **4a**–**j** occurred using iron powder and NH_4_Cl in a mixture of methanol/water. In the final step, condensation of compounds **4a**–**j** with the commercially available ethyl 4-methyl-3-((4-(pyridin-3-yl)pyrimidin-2-yl)amino)benzoate **5** in dry THF and in the presence of potassium tert-butoxide (*t*-BuOK) gave the final hybrid compounds **1a**–**j**, also characterized by their ^1^H and ^13^C NMR spectroscopic data (full spectra in Figures S1–S20).

### 2.2. TK and HO-1 Inhibition

Compounds **1a**–**j** were designed and synthesized as novel NIL derivatives, bearing a phenylaminopyrimidine portion and an additional aryloxyalkylimidazole moiety. Novel compounds were tested to investigate their ability to inhibit both BCR-ABL TK and HO-1. A FRET-based Z’-Lyte assay was used to evaluate the TK inhibitory activity of compounds **1a**–**j**. The results are shown in Table 1 and are expressed as IC_50_ values (μM), compared to those of NIL and IM. Most of the novel derivatives showed a good level of potency inhibiting BCR-ABL in the micromolar range. In particular, noteworthy results were obtained with compounds **1a**, **1e**, and **1g**, which stand out as being active in the nanomolar range. In fact, these last three compounds were more potent TKIs than IM with IC_50_ values ranging from 0.037 to 0.109 μM. Moreover, the activity of compound **1e** is similar to NIL (IC_50_ = 0.037 and 0.039 μM, respectively), resulting the most potent of the whole series. The position of the oxybutylimidazole moiety in the benzamide ring strongly affected the inhibitory properties of these compounds. Derivatives with the highest TKI activity carry the oxybutylimidazole moiety at 4- or 3-position (TK IC_50_ **1a**, **1e**, and **1g** = 0.109, 0.037, and 0.077 μM, respectively), whereas derivatives with the lowest TKI activity were 2-oxyalkyl-substituted (TK IC_50_ **1c** and **1i** = 14.65 and 13.67 μM, respectively) or carried a 2-bromine residue (TK IC_50_ **1d** = 16.16 μM). These data indicate that the binding with the protein is not favored by the presence of any type of ortho-substitution of the benzamide ring. Introduction of a bromine, methyl, or trifluoromethyl R substituent at various positions of the benzamide ring of **1a**–**c** did not afford homogeneous results but improved activity in the case of 4-oxybutyl derivatives (**1e** vs. **1a**), 3-oxybutyl analogs (**1b** vs. **1g** and **1h**), and 2-oxybutyl analog (**1c** vs. **1j**), whereas an opposite trend was observed in the case of other 3-oxybutyl and 2-oxybutyl analogs (**1b** vs. **1f** and **1c** vs. **1i**, respectively).

HO-1 activity was measured in microsomal fractions of rat spleen obtained as described in the experimental section [13]. The activity was determined by measuring the bilirubin formation using the difference in absorbance at 464–530 nm. HO-1 inhibitors **LS/0** and **LS1/71** were used as reference compounds. HO-1 inhibition from compounds **1a**–**j** is expressed as IC_50_ values (μM), and results are summarized in Table 1. Novel derivatives were less potent than the reference compounds and the previous IM-based hybrids [28]. Nevertheless, some observed differences in the results among the tested compounds allow us to discuss SAR as follows. HO-1 inhibitory activity mainly depends on the position of the oxybutylimidazole moiety and only to a lesser extent on the R substituent on the benzamide ring. On the whole, the presence of the oxybutylimidazole moiety at the 4-position of the benzamide ring gave the best contribution (**1a** HO-1 IC_50_ = 44.79 μM). Conversely, compounds with the oxybutylimidazole moiety at the 3- and 2-position (**1b** and **1c**) gave higher HO-1 IC_50_ values. These results contrast with the previous IM-based series, where the 2-oxybutyl derivatives were more potent than 4- and 3-substituted analogs. Furthermore, the introduction of a substituent in the benzamide ring did not remarkably change this rule. In general, the bromine residue gave the best contribution as demonstrated by 3- and 5-bromine-derivatives **1e** and **1g** (HO-1 IC_50_ value 55.14 and 50.95 μM, respectively). However, for the 4-bromine-2-oxybutyl derivative **1i**, a drop in HO-1 inhibition was observed (HO-1 IC_50_ = 83.44 μM); this trend was further emphasized for 5-methyl-2oxybutyl derivative **1j** (HO-1 IC_50_ about 3 mM). The introduction of a trifluoromethyl group as a substituent, selected because present in the structure of NIL, provided only moderate HO-1 inhibitory activity (**1h**, HO-1 IC_50_ = 65.36 μM). Another difference from the previous IM-based hybrids is that unsubstituted compounds **1a** and **1b** were more potent than substituted ones **1d**–**1j** (except for **1e** and **1g**). 

### 2.3. In Vitro Cytotoxic Activity on K562S and K562R Cells

**Figure 2:**
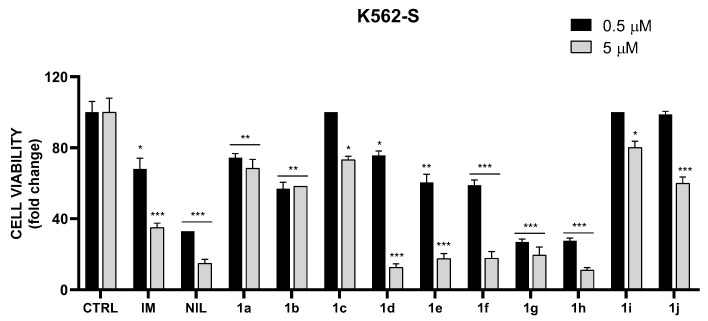
Effects of derivatives **1a**–**j** on cell viability of K562-S cell line at 0.5 and 5 μM concentration. *** *p* ≤ 0.0005 vs. CTR, ** *p* ≤ 0.005 vs. CTRL, * *p* ≤ 0.05 vs. CTRL.

These novel NIL-based compounds were developed as potential anticancer agents, and thus measurement of the cytotoxicity of all new compounds was assessed in K562 CML cells, both NIL-sensitive (K562-S) and -resistant (K562-R). We found that many derivatives influenced proliferation of sensitive cells; in particular, **1g** and **1h** showed a significant reduction in K562-S cell viability, comparable to NIL and better than IM at the same concentrations (Figure 2). Since HO-1 overexpression in IM-resistant cells is well documented [23,24,25], and due to previous published TK/HO-1 IM-based hybrids (Figure 1) [28] showing strong efficacy against K562 IM-resistant cells, we hypothesized that new NIL-based hybrids **1a**–**j** could be effective also in the case of NIL resistance. Moreover, few studies report on the involvement of HO-1 in NIL resistance [27]. Therefore, we established K562 cell line resistance to NIL and investigated the involvement of HO-1 overexpression as one of the possible mechanisms of resistance. When NIL resistance was achieved, compounds **1a**–**j** were tested on K562-R cells at a 20 nM concentration (close enough to NIL IC_50_), and in this case, they did not show a significant reduction in cell viability (Figures S21 and S22). Moreover, Western blot analysis was performed to establish HO-1 involvement in NIL resistance. As shown in Figure S23, HO-1 expression did not change depending on resistance establishment, demonstrating that HO-1 overexpression is not involved in K562 cell NIL resistance and suggesting an HO-1-independent mechanism behind it. However, compounds **1g** and **1h** developed in the present study stand out for their potency as antiproliferative agents being more effective than IM in K562-S cells. Therefore, these new molecules may be considered as promising leading compounds for the development of new BCR-ABL inhibitors as anticancer agents.

### 2.4. Docking Studies

A molecular docking study was performed to highlight the interaction of the new set of compounds with BCR-ABL kinase and HO-1. The binding modes of all new molecules were studied in comparison with IM in BCR-ABL kinase and (2*R*,4*S*)-2-[2-(4-chlorophenyl)ethyl]-2-[(1H-imidazol-1-yl)methyl]-4-[((5-trifluoromethylpyridin-2-yl)thio)methyl]-1,3-dioxolane (QC-80) in HO-1. The X-ray crystal structures of the BCR-ABL kinase domain in complex with IM (PDB ID: 1IEP) [32] and HO-1/QC-80 (PDB ID: 3HOK) [33] were used as the protein structures. The docking procedure was validated by comparing the experimental and calculated binding potencies and the RMSD of IM and QC-80 to their original binding sites of the respective proteins. To validate the BCR-ABL kinase model, we compared our results with those of Lin et al. [34] and the ones previously reported by us [28]. Moreover, the same validated docking procedure as previously published by our research group for validating the HO-1 model was used [35,36,37]. Our docking-derived calculated binding potencies are in good agreement with the experimental ones as reported in Table 2 and Table 3. The simulated complexes of IM and QC-80 with the respective co-crystallized protein show high correspondence to the corresponding crystallographic structure with an RMSD value of 0.76 Å and 0.75 Å, respectively. Once the models were validated, the set of compounds (**1a**–**j**) were docked inside the binding pocket of BCR-ABL kinase and HO-1. The obtained poses and molecular interactions for all the studied molecules are reported in the supplementary material (Figures S24–S44). 

#### 2.4.1. Docking Studies on BCR-ABL Kinase

**Figure 3:**
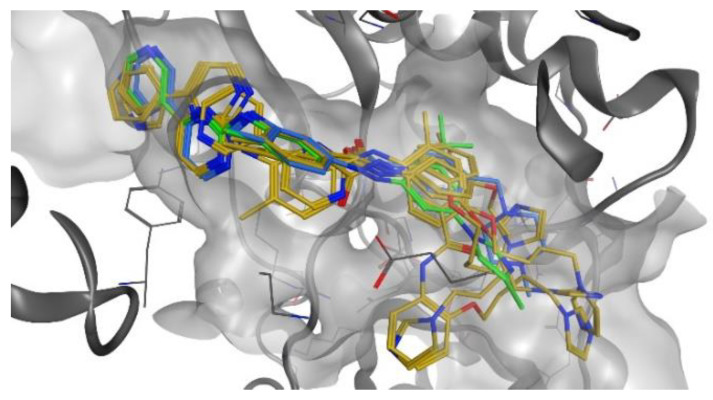
Binding poses of selected compounds **1a**, **1c**, **1e**, **1g**, and **1j** (yellow) inside the binding pocket of BCR-ABL. IM (blue), NIL (green). For detailed interaction information, see supplementary materials.

The calculated energies of binding and the poses of the studied compounds for BCR-ABL kinase are shown in Table 2 and Figure 3 and Figures S24–S34. Results of calculated binding energies for the molecules shown in Table 2 indicate a high correlation between the estimated free energies of binding and the experimentally measured IC_50_. All the studied molecules are able to interact with the protein in a similar way to that of reference compounds IM and NIL. Interestingly, only the compounds **1b**, **1c**, and **1d** cannot properly allocate the IM-like portion of the molecule inside the binding pocket and cannot achieve the optimal interactions for the kinase inhibition, compared to the most potent compounds of the series. Particularly, compounds **1a**, **1e**, **1g**, and **1h** reduced TK activity better than IM (Figures S25, S29, S31 and S32), compared to all the other compounds that possess a lower activity than IM. The N-(4-methyl-3-((4-(pyridin-3-yl)pyrimidin-2-yl)amino)phenyl) moiety of molecules **1a**, **1e**, **1g**, and **1j** is always located in the same pocket formed by Ile313, Tyr253, Leu248, Phe317, Met318, Leu370, Val256, and Thr315. Differently, as already mentioned, the less potent compound **1c**, due to the presence of the oxybutylimidazole in 2-position, is not able to allocate the same portion correctly inside the binding pocket, pushing the molecule more externally toward the surface of the protein. Otherwise, the oxybutylimidazole group may be directed in different ways in relation to its position on the benzamide ring but always points to the protein’s external surface in all docked compounds. Judging by the molecular modeling result and looking at the experimental IC_50_, we can conclude that the oxybutylimidazole moiety can be placed in 3- and 4-positions without any loss of activity and any relevant change to the central core of the “IM-like portion” of the molecule location inside BCR-ABL. Differently, the positioning of the oxybutylimidazole in 2-position will result in a steric hindrance of the molecule that will not be able to allocate itself properly, resulting in a loss of activity. Nevertheless, some significant differences can be achieved with a different allocation of the R substituents. For example, the methyl group in 5-position of **1j** decreases the molecule’s activity for a steric clash with Leu298 and Ile293. Interestingly, the -CF_3_ group of **1h** could be easily allocated inside a pocket formed by Val379, Leu298, and Thr351, which is the same pocket that accommodates the -CF_3_ group of nilotinib in the crystal structure. In addition, the bromine in 3/5-position of **1e** and **1g** is well accommodated in a near pocket formed by Ile293, Val299, His361, and Ala380. When the bromine is allocated in this pocket, both molecules can interact with Asp381 (H-bond donor) and Glu286 (H-bond acceptor); moreover, the more potent **1e** is able to allocate the IM-like portion exactly like the IM and NIL, and it can achieve another set of stabilizing interaction inside the binding pocket (i.e., Met318 (H-bond donor), Val256 (H-Pi interaction), and Thr315 (H-bond acceptor). Differently, when the bromine is in 4-position (Gly383, Ile293, Leu354, Val289) as in molecule **1i**, the compound is not able to reach optimal activity due also to the presence of the oxybutylimidazole in 2-position. Having the bromine in the same 4-position allocated inside the same pocket formed by Ile293, Leu354, Val289, the oxybutylimidazole in 3-position would result in a slightly more active compound as measured by molecule **1f**.

#### 2.4.2. Docking Studies on HO-1

**Figure 4:**
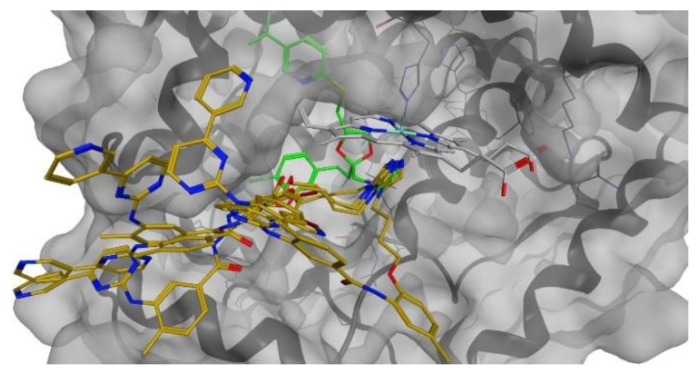
Binding poses of selected compounds **1a**, **1c**, **1e**, **1g**, and **1j** (yellow) inside the binding pocket of HO-1. QC-80 (green). For detailed interaction information, see supplementary materials.

The results of the docking calculation and the docking poses for HO-1 are reported in Table 3 and Figure 4 and Figures S35–S44, respectively. The molecules were initially docked into the binding site of HO-1, and then the best pose was minimized toward an MD simulation of 50 ns. After the MD simulation, a rescoring of all the molecules allowed obtaining a good agreement between the final calculated energies of binding and the IC_50_ experimental values that emerged from the HO-1 inhibition assay, as reported in Table 3. The iron(II) of the prosthetic group in HO-1 is precisely coordinated by the imidazole (nitrogen atom) ring of all the studied compounds in the eastern pocket. The result of this interaction is that the coordinated iron is now protected from oxidation by the disruption of an ordered solvent structure involving the crucial Asp140 hydrogen-bond network (Tyr58, Tyr114, Arg136, and Asn210) and resulting in a displacement of water molecules inside the binding pocket needed for the physiological catalysis. On the other hand, the position of the oxybutylimidazole and the R group can slightly influence the potency and the dual activity of molecules, but still, none of the molecules were found to be potent HO-1 inhibitors, differently from a similar set of compounds published by us that revealed a good to excellent inhibition of HO-1 [28]. In the case at hand, the MD simulation studies allowed us to understand the lower activity. In fact, despite the good initial static occupation of the catalytic site obtained by the docking calculation, the MD simulation allowed us to study the compounds over time dynamically, and, in 50 ns, none of the studied compounds was able to effectively remain in the western or northeastern regions of the HO-1 binding pocket. As shown by the binding poses in the reported Figure 4 and Figures S35–S44, the only stabilizing interactions of the whole set of molecules were achieved with amino acids in the protein’s surface, generally resulting in low potency. The less potent compound **1j** (Figure S44) due to the steric hindrance caused by the position of the oxybutylimidazole group and the R substituent is unable to perform any stabilizing interaction, not even with the surface of the protein; this determines the very low activity found, unlike all the other compounds studied which are partially able to interact with the surface of the protein.

In conclusion, the docking calculation and MD simulation allow explaining the low activity toward the HO-1, indicating that despite some interaction with the surface of the protein, the molecules are not able to achieve good potency due to the impossibility to interact appropriately with the hydrophobic binding pocket of HO-1 (i.e., the western and the northeastern regions).

## 3. Materials and Methods

### 3.1. Chemistry

Melting points were determined by using an Electrothermal IA9200 apparatus containing a digital thermometer. Determinations were achieved after introducing glass capillary tubes, filled with analytes, inside the apparatus. ^1^H NMR and ^13^C NMR spectra were recorded on Varian Inova Unity (200 and 500 MHz) spectrometers in DMSO-*d*_6_ or CD_3_OD solutions. Chemical shifts are given in δ values to two digits after the decimal point in part per million (ppm), using tetramethylsilane (TMS) as the internal standard; coupling constants (J) are given in Hz. Signal multiplicities are indicated with the following abbreviations: s (singlet), d (doublet), t (triplet), q (quartet), m (multiplet), br (broad signal). Reactions were monitored by thin-layer chromatography (TLC), carried out on Merck plates (Kieselgel 60 F_254_), using UV light (254 nm and 366 nm) for visualization, and developed using iodine chamber. Flash column chromatography was performed on Merck silica gel 60 0.040–0.063 mm (230–400 mesh). Biotage^®^ SNAP cartridges KP-Sil were also used. Synthetic procedures achieved through microwave were performed with a CEM Discover instrument using closed Pyrex glass tubes (ca. 10 mL) with Teflon-coated septa. Reagents, solvents, and starting materials were purchased from commercial suppliers. Synthesis and characterization of all intermediates are described in Supplementary Material.

#### General Procedure for the Synthesis of Final Compounds (**1a**–**j**)

Ethyl 4-methyl-3-((4-(pyridin-3-yl)pyrimidin-2-yl)amino)benzoate **5** (1.58 mmol) was dissolved in THF (20 mL). A solution of the appropriate imidazolylbutoxy aniline **4a**–**j** in THF (10 mL) was added under nitrogen atmosphere. After 5 min, *t*-BuOK (7.93 mmol) was added, and the reaction mixture was stirred at room temperature overnight. Water was added, and the resulted aqueous layer was extracted with EtOAc (3 × 50 mL). The combined extracts were washed with brine, dried on anhydrous Na_2_SO_4_, and evaporated to obtain a residue, which was purified by flash column chromatography on silica gel using 8.5 EtOAc:1.5 CH_3_OH as eluent.

*N*-(4-(4-(1H-imidazol-1-yl)butoxy)phenyl)-4-methyl-3-((4-(pyridin-3-yl)pyrimidin-2-yl)amino)benzamide (**1a**).

Brown solid; mp 140–142 °C; yield 25.3%. ^1^H NMR (500 MHz, DMSO-*d*_6_): δ 10.07 (s, 1H), 9.27 (s, 1H), 9.11 (s, 1H), 8.69–8.68 (m, 1H), 8.54 (d, *J* = 5 Hz, 1H), 8.45–8.43 (m, 1H), 8.25 (s, 1H), 7.73–7.65 (m, 4H), 7.52–7.39 (m, 2H + 1H), 7.19 (s, 1H), 6.92–6.90 (m, 3H), 4.03 (t, *J* = 5 Hz, 2H), 3.96 (t, *J* = 5 Hz, 2H), 2.34 (s, 3H), 1.89–1.83 (m, 2H), 1.67–1.62 (m, 2H). ^13^C NMR (125 MHz, DMSO-*d*_6_): δ 164.8, 161.6, 161.1, 159.6, 154.8, 151.4, 148.1, 138.0, 137.2, 136.0, 134.3, 132.8, 132.3, 132.2, 130.2, 128.4, 124.2, 123.8, 123.4, 122.0, 119.3, 114.3, 107.8, 67.1, 45.7, 27.4, 25.8, 18.2. Anal. Calcd. for (C_30_H_29_N_7_O_2_): C, 69.35; H, 5.63; N, 18.87. Found: C, 69.09; H, 5.65; N, 18.82.

*N*-(3-(4-(1H-imidazol-1-yl)butoxy)phenyl)-4-methyl-3-((4-(pyridin-3-yl)pyrimidin-2-yl)amino)benzamide (**1b**).

Brown solid; mp 118–120 °C; yield 26%. ^1^H NMR (500 MHz, DMSO-*d*_6_): δ 10.15 (s, 1H), 9.23 (s, 1H), 9.07 (s, 1H), 8.66–8.64 (m, 1H), 8.50 (d, *J* = 5 Hz, 1H), 8.42–8.40 (m, 1H), 8.24 (s, 1H), 7.69–7.67 (m, 2H), 7.49–7.47 (m, 1H), 7.43–7.38 (m, 3H), 7.33–7.31 (m, 1H), 7.23–7.18 (m, 2H), 6.90 (s, 1H), 6.66–6.64 (m, 1H), 4.01 (t, *J* = 5 Hz, 2H), 3.93 (t, *J* = 5 Hz, 2H), 2.32 (s, 3H), 1.86–1.81 (m, 2H), 1.66–1.60 (m, 2H). ^13^C NMR (125 MHz, DMSO-*d*_6_): δ 165.7, 161.9, 161.3, 159.8, 159.0, 151.7, 148.3, 140.5, 138.2, 136.5, 134.7, 132.9, 132.4, 130.6, 129.7, 128.3, 124.4, 124.2, 123.7, 119.7, 113.0, 110.0, 108.2, 107.1, 67.2, 46.1, 27.6, 26.0, 18.4. Anal. Calcd. for (C_30_H_29_N_7_O_2_): C, 69.35; H, 5.63; N, 18.87. Found: C, 69.12; H, 5.64; N, 18.91.

*N*-(2-(4-(1H-imidazol-1-yl)butoxy)phenyl)-4-methyl-3-((4-(pyridin-3-yl)pyrimidin-2-yl)amino)benzamide (**1c**).

Orange oil; yield 20%. ^1^H NMR (500 MHz, CD_3_OD): δ 9.21 (s, 1H), 8.57 (m, 1H), 8.50–8.44 (m, 2H + 1H), 7.96 (d, *J* = 10 Hz, 1H), 7.56 (d, *J* = 10 Hz, 1H), 7.49 (s, 1H), 7.44–7.42 (m, 1H), 7.36–7.32 (m, 2H), 7.13–7.10 (m, 1H), 6.98–6.93 (m, 3H), 6.84 (s, 1H), 3.98 (t, *J* = 5 Hz, 2H), 3.92 (t, *J* = 5 Hz, 2H), 2.38 (s, 3H), 1.89–1.83 (m, 2H), 1.70–1.64 (m, 2H). ^13^C NMR (125 MHz, CD_3_OD): δ 167.7, 163.5, 162.3, 160.4, 151.9, 151.5, 148.9, 139.5, 136.6, 136.5, 134.3, 134.2, 132.0, 129.0, 128.3, 126.8, 125.4, 124.1, 123.8, 123.7, 121.8, 120.4, 113.1, 109.2, 69.0, 47.7, 28.9, 27.4, 18.4. Anal. Calcd. For (C_30_H_29_N_7_O_2_): C, 69.35; H, 5.63; N, 18.87. Found: C, 69.18; H, 5.61; N, 18.89.

*N*-(5-(4-(1H-imidazol-1-yl)butoxy)-2-bromophenyl)-4-methyl-3-((4-(pyridin-3-yl)pyrimidin-2-yl)amino)benzamide (**1d**).

Yellow solid; mp 69.8–72.0 °C; yield 23.5%. ^1^H NMR (500 MHz, CDCl_3_): δ 9.27 (s, 1H), 8.93 (s, 1H), 8.71 (d, *J* = 5 Hz, 1H), 8.54 (d, *J* = 5 Hz, 1H), 8.41–8.38 (m, 1H), 8.33 (s, 1H), 8.28 (s, 1H), 7.61–7.59 (m, 1H), 7.42–7.36 (m, 2H), 7.24–7.22 (m, 2H), 7.16 (s, 1H), 7.08 (s, 1H), 6.57 (s, 1H), 4.20 (t, *J* = 5 Hz, 2H), 4.05 (t, *J* = 5 Hz, 2H), 3.65 (s, 2H), 2.45 (s, 3H), 2.09–2.03 (m, 2H), 1.85–1.80 (m, 2H). ^13^C NMR (125 MHz, CDCl_3_): δ 165.4, 163.0, 160.6, 159.2, 158.8, 151.7, 148.7, 138.3, 136.8, 134.8, 133.1, 132.6, 132.5, 131.2, 125.6, 123.9, 121.9, 119.8, 119.7, 112.2, 109.0, 107.4, 104.3, 67.5, 48.0, 29.8, 27.9, 26.1, 18.4. Anal. Calcd. for (C_30_H_28_BrN_7_O_2_): C, 60.21; H, 4.72; N, 16.38. Found: C, 60.32; H, 4.70; N, 16.42.

*N*-(4-(4-(1H-imidazol-1-yl)butoxy)-3-bromophenyl)-4-methyl-3-((4-(pyridin-3-yl)pyrimidin-2-yl)amino)benzamide (**1e**).

Yellow oil; yield 25%. ^1^H NMR (500 MHz, CD_3_OD): δ 9.24 (s, 1H), 8.63–8.62 (m, 1H), 8.54–8.52 (m, 1H), 8.47 (d, *J* = 5 Hz, 1H), 8.36 (s, 1H), 7.94 (s, 1H), 7.79 (s, 1H), 7.65–7.50 (m, 3H), 7.40–7.36 (m, 2H), 7.20 (s, 1H), 7.02–6.98 (m, 2H), 4.17 (t, *J* = 5 Hz, 2H), 4.05 (t, *J* = 5 Hz, 2H), 2.39 (s, 3H), 2.08–2.01 (m, 2H), 1.82–1.77 (m, 2H). ^13^C NMR (125 MHz, CD_3_OD): δ 168.4, 163.7, 162.5, 160.4, 153.5, 151.9, 148.9, 139.3, 137.2, 136.6, 134.5, 134.2, 134.1, 131.8, 128.4, 127.3, 125.4, 124.6, 124.5, 122.7, 120.8, 114.5, 112.6, 109.1, 70.0, 48.0, 29.0, 27.1, 18.4. Anal. Calcd. for (C_30_H_28_BrN_7_O_2_): C, 60.21; H, 4.72; N, 16.38. Found: C, 60.39; H, 4.71; N, 16.45.

*N*-(3-(4-(1H-imidazol-1-yl)butoxy)-4-bromophenyl)-4-methyl-3-((4-(pyridin-3-yl)pyrimidin-2-yl)amino)benzamide (**1f**).

Orange oil; yield 30%. ^1^H NMR (500 MHz, DMSO-*d*_6_): δ 10.25 (s, 1H), 9.27 (s, 1H), 9.13 (s, 1H), 8.68 (d, *J* = 5 Hz, 1H), 8.54 (d, *J* = 5 Hz, 1H), 8.45–8.43 (m, 1H), 8.27 (s, 1H), 7.73–7.71 (m, 1H), 7.64 (br s, 2H), 7.52–7.47 (m, 3H), 7.43–7.38 (m, 2H), 7.18 (s, 1H), 6.89 (s, 1H, 4.08–4.03 (m, 4H), 2.35 (s, 3H), 1.94–1.88 (m, 2H), 1.73–1.68 (m, 2H). ^13^C NMR (125 MHz, DMSO-*d*_6_): δ 165.2, 161.6, 161.0, 159.5, 154.5, 151.4, 148.1, 140.0, 138.0, 137.2, 136.4, 134.3, 132.5, 132.4, 132.1, 130.3, 128.4, 124.2, 123.8, 123.4, 119.2, 113.7, 107.9, 105.9, 104.6, 68.0, 45.6, 27.3, 25.6, 18.2. Anal. Calcd. For (C_30_H_28_BrN_7_O_2_): C, 60.21; H, 4.72; N, 16.38. Found: C, 60.26; H, 4.70; N, 16.39.

*N*-(3-(4-(1H-imidazol-1-yl)butoxy)-5-bromophenyl)-4-methyl-3-((4-(pyridin-3-yl)pyrimidin-2-yl)amino)benzamide (**1g**).

Yellow solid; mp 162.0–164.4 °C; yield 28%. ^1^H NMR (500 MHz, DMSO-*d*_6_): δ 10.24 (s, 1H), 9.27 (s, 1H), 9.13 (s, 1H), 8.68 (br s, 1H), 8.54 (d, *J* = 5 Hz, 1H), 8.45–8.42 (m, 1H), 8.26 (s, 1H), 7.71–7.64 (m, 3H), 7.52–7.41 (m, 4H), 7.19 (s, 1H), 6.88 (d, *J* = 5 Hz, 2H), 4.04–3.97 (m, 4H), 2.34 (s, 3H), 1.88–1.82 (m, 2H), 1.67–1.62 (m, 2H). ^13^C NMR (125 MHz, DMSO-*d*_6_): δ 165.4, 161.6, 161.0, 159.6, 159.5, 151.4, 148.1, 141.5, 138.1, 137.2, 136.5, 134.3, 132.2, 132.1, 130.3, 128.4, 124.2, 123.8, 123.5, 121.7, 119.2, 115.0, 112.4, 107.9, 105.6, 67.4, 45.6, 27.3, 25.6, 18.2. Anal. Calcd. For (C_30_H_28_BrN_7_O_2_): C, 60.21; H, 4.72; N, 16.38. Found: C, 60.15; H, 4.74; N, 16.33.

*N*-(3-(4-(1H-imidazol-1-yl)butoxy)-5-(trifluoromethyl)phenyl)-4-methyl-3-((4-(pyridin-3-yl)pyrimidin-2-yl)amino)benzamide (**1h**). 

Yellow solid; mp 180.3–182.5 °C; yield 27%. ^1^H NMR (500 MHz, DMSO-*d*_6_): δ 10.41 (s, 1H), 9.27 (s, 1H), 9.14 (s, 1H), 8.68 (br s, 1H), 8.55 (d, *J* = 5 Hz, 1H), 8.45–8.43 (m, 1H), 8.29 (s, 1H), 7.81–7.65 (m, 4H), 7.52–7.43 (m, 3H), 7.19 (s, 1H), 6.96 (s, 1H), 6.89 (s, 1H), 4.07–4.03 (m, 4H), 2.35 (s, 3H), 1.91–1.85 (m, 2H), 1.71–1.65 (m, 2H). ^13^C NMR (125 MHz, DMSO-*d*_6_): δ 165.6, 161.6, 161.0, 159.6, 159.2, 151.4, 148.1, 141.2, 138.1, 137.2, 136.7, 134.3, 132.1, 130.4, 130.1, 128.4, 125.1, 124.3, 123.8, 123.5, 119.2, 109.8, 108.8, 105.9, 67.5, 45.6, 27.3, 25.6, 18.2. Anal. Calcd. For (C_31_H_28_F_3_N_7_O_2_): C, 63.37; H, 4.80; N, 16.69. Found: C, 63.22; H, 4.82; N, 16.74.

*N*-(2-(4-(1H-imidazol-1-yl)butoxy)-4-bromophenyl)-4-methyl-3-((4-(pyridin-3-yl)pyrimidin-2-yl)amino)benzamide (**1i**).

Yellow solid; mp 94–96 °C; yield 20%. ^1^H NMR (200 MHz, DMSO-*d*_6_): δ 9.38 (s, 1H), 9.27 (s, 1H), 9.16 (s, 1H), 8.70–8.67 (m, 1H), 8.54 (d, *J* = 4 Hz, 1H), 8.47–8.41 (m, 1H), 8.23 (s, 1H), 7.74–7.62 (m, 3H), 7.54–7.37 (m, 3H), 7.27–7.10 (m, 3H), 6.88 (s, 1H), 4.07–3.91 (m, 4H), 2.35 (s, 3H), 1.86–1.75 (m, 2H), 1.66–1.56 (m, 2H). ^13^C NMR (125 MHz, CDCl_3_): δ 165.3, 162.8, 160.7, 159.3, 151.7, 148.5, 148.3, 138.3, 134.8, 133.5, 133.1, 132.6, 131.2, 127.2, 125.9, 124.5, 123.9, 121.9, 120.4, 119.3, 116.5, 114.8, 108.7, 68.1, 47.7, 27.7, 26.2, 18.4. Anal. Calcd. for (C_30_H_28_BrN_7_O_2_): C, 60.21; H, 4.72; N, 16.38. Found: C, 59.98; H, 4.73; N, 16.41.

*N*-(2-(4-(1H-imidazol-1-yl)butoxy)-5-methylphenyl)-4-methyl-3-((4-(pyridin-3-yl)pyrimidin-2-yl)amino)benzamide (**1j**).

Brown oil; yield 18%. ^1^H NMR (200 MHz, DMSO-*d*_6_): δ 9.26–9.18 (m, 2H + 1H), 8.70–8.68 (m, 1H), 8.67–8.53 (m, 2H), 8.23 (s, 1H), 7.64–7.37 (m, 6H), 7.05–6.82 (m, 4H), 3.98–3.88 (m, 4H), 2.35 (s, 3H), 2.26 (s, 3H), 1.84–1.74 (m, 2H), 1.63–1.57 (m, 2H). ^13^C NMR (50 MHz, CD_3_OD): δ 167.6, 163.5, 162.3, 160.4, 151.8, 149.3, 148.9, 139.4, 136.6, 136.5, 134.3, 134.2, 132.0, 131.3, 129.0, 127.9, 127.0, 125.4, 124.6, 123.8, 123.7, 120.4, 112.9, 109.2, 69.0, 28.9, 27.4, 20.9, 18.4. Anal. Calcd. For (C_31_H_31_N_7_O_2_): C, 69.77; H, 5.86; N, 18.37. Found: C, 70.01; H, 5.84; N, 18.32.

### 3.2. Biology

#### 3.2.1. Preparation of Spleen Microsomal Fractions

Since HO-1 protein is abundant in the rat spleen as well documented [38], rat spleen microsomal fraction prepared by differential centrifugation was used as source of the HO-1 protein. This microsomal preparation represents the most native (i.e., closest to in vivo) form of HO-1. Spleen microsomal fractions were obtained according to Ryter et al. [13]. The experiments reported in the present paper complied with current Italian law and met the guidelines of the Institutional Animal Care and Use Committee of Ministry of Health (Directorate General for Animal Health and Veterinary Medicines, Italy) “Dosing of enzymatic activities in rat microsomes” (2018–2022), project code 02769.N.VLY. Male Sprague–Dawley albino rats (150 g body weight and age 45 d) used in our experimental conditions to obtain spleen microsomal fraction had free access to water and were kept at room temperature with a natural photo-period (12 h light–12 h dark cycle). To obtain a homogenate (15%, *w/v*) of spleens, each rat was sacrificed, and its spleen was excised and weighed. Spleens pooled from five rats were homogenized in 50 mM Tris buffer, pH 7.4, containing 0.25 M sucrose, using a Potter–Elvehjem homogenizing system with a Teflon pestle. The microsomal fraction of rat spleen homogenate was obtained by differential centrifugation: first centrifugation at 10,000× *g* for 20 min at 4 °C and a second centrifugation of the supernatant at 100,000× *g* for 60 min at 4 °C. The 100,000× *g* pellet (microsomes) was resuspended in 100 mM potassium phosphate buffer, pH 7.8, containing 2 mM MgCl_2_. The rat spleen fraction was divided into aliquots and stored at −80 °C for up to 2 months, and then its protein concentration was measured. 

#### 3.2.2. Preparation of Biliverdin Reductase

Biliverdin reductase was obtained by using rat liver cytosol. Three volumes of a solution 1.15% KCl *w/v* and Tris buffer 20 mM, pH 7.8, were used to homogenize on ice liver tissue. The 10,000× *g* supernatant was obtained by differential centrifugation: first centrifugation at 10,000× *g* for 20 min at 4 °C and a second centrifugation at 10,000× *g* for 1 h at 4 °C to sediment the microsomes. The 10,000× *g* supernatant was stored in small amounts at −80 °C, and then its protein concentration was measured.

#### 3.2.3. Measurement of HO-1 Enzymatic Activity in Microsomal Fraction of Rat Spleen

The HO-1 activity was determined by measuring the bilirubin formation using the difference in absorbance at 464–530 nm, as described by Ryter et al. [13]. A final volume of 500 μL of incubation mixtures contained 20 mM Tris–HCl, pH 7.4, (1 mg/mL) microsomal extract, 0.5–2 mg/mL biliverdin reductase, 1 mM NADPH, 2 mM glucose 6-phosphate (G6P), 1 U G6P dehydrogenase, 25 μM hemin, and 10 μL of DMSO (or the same volume of DMSO solution of test compounds to a final concentration of 100, 10, and 1 μM). Enzymatic reactions were carried out in the dark for 60 min at 37 °C in a circulating water bath. The reactions were stopped by the addition of 500 μL of chloroform. After recovering the chloroform phase, the amount of bilirubin formed was measured with a double-beam spectrophotometer as OD464–530 nm (extinction coefficient, 40 mM/cm^−1^ for bilirubin). The enzymatic activity was defined as Units (one Unit is the amount of enzyme catalyzing the formation of 1 nmol of bilirubin/mg protein/h).

#### 3.2.4. Cell Cultures

K562 cells (CCL-243 ATCC) were cultured in RPMI 1640 (L0500, Biowest, Riverside, MO, USA) supplemented with 10% Fetal Bovine Serum (FBS) and 1% penicillin–streptomycin and incubated at 37 °C in 5% CO_2_. K562 cells were seeded at a concentration of 1 × 10^5^ cells/mL of culture medium. The NIL-resistant sub-line (K562-R) was obtained following a protocol of exposition to increasing concentrations of NIL, monitoring cell proliferation and viability with trypan blue exclusion method every 48 h. Briefly, cells were harvested by centrifugation and resuspended in 5 mL of fresh medium; then an aliquot of 10 μL was taken and mixed with Trypan Blue (93595, Sigma-Aldrich, Milan, Italy) stain (1:1). The mixture was applied on a counting chamber, and cells were counted on a binocular microscope. The parental, sensitive cells (K562-S) were maintained in parallel cultures without NIL to be used as controls. The initial NIL concentration used was 5 nM, and then it was increased in each step until 200 nM. Once resistance was established (after approximately 9 months), K562-R and K562-S were treated with compounds at different concentrations.

#### 3.2.5. In Vitro Cytotoxicity

K562-R cells were treated with compounds **1a**–**j** and NIL (079266, Fluorochem, Hadfield UK) at a concentration of 20 nM (close enough to NIL IC_50_). Sensitive K562 cells were treated with the same compounds and NIL or IM at 5 and 0.5 μM concentrations. Treatments were maintained for 48 h, and then cell viability was assessed: (a) with 0.2% (final concentration) trypan blue dye exclusion analysis which is used to determine the number of viable cells present in a cell suspension and (b) with XTT assay.

(a) Briefly, the cell number was determined by counting the viable cells in a Burker counting chamber divided into 16 fields of 1 mm^2^. The percentage of viable cells was obtained by applying the following equation: % viable cells = (VC/TC) × 100, where VC = viable cells counted and TC = total cells counted (stained plus unstained cells).
(b) The XTT (2,3-bis-(2-methoxy-4-nitro-5-sulfophenyl)-2H-tetrazolium-5-carboxanilide) assay is based on the extracellular reduction of XTT by NADH produced in the mitochondria via trans-plasma membrane electron transport and an electron mediator. Reduction of XTT during the assay produces a water-soluble orange-colored formazan product. The amount of formazan was proportionate to the number of viable cells in the sample. Finally, absorbance (OD) was measured in a microplate reader (Biotek Synergy-HT, Winooski, VT, USA) at λ = 450 nm.

Results are expressed as mean ± SD.

#### 3.2.6. FRET-Based Z′-Lyte Assay

The effect of compounds on the kinase activity of BCR-ABL was assessed in 384-well black plates using the FRET-based Z′-Lyte Kinase Assay Kit–Tyr 2 Peptide according to the manufacturer’s instructions (Invitrogen, Carlsbad, CA, USA). IM and NIL were used as positive controls to validate the screening assay. Briefly, 10 μL per well reaction contained 12 μM ATP, 2 μM Tyr 2 Peptide, 0.0247 μg/mL ABL-1 (P3049-10 μg, Life Technologies, Waltham, MA, USA) inhibitors as appropriate. The final concentrations tested for each compound were 0.005, 0.5, 5, 25, 50 μM. The reaction was performed at room temperature for 1 h; then 5 μL of development reagent was added and incubated for another 1 h at room temperature, followed by the addition of 5 μL of stop solution. Fluorescence signal ratio of 445 nm (coumarin)/520 nm (fluorescein) was detected using a microplate reader (Biotek Synergy-HT, Winooski, VT, USA). Data express the mean values of three different experiments; every test was conducted in quadruplicate.

#### 3.2.7. Western Blot

Western blot analysis was performed to assess HO-1 protein levels following resistance induction; in particular, protein samples were collected from cells resistant to 50, 100, 150, and 200 nM. Cells were harvested, and pellets were sonicated and centrifugated at 2500 rpm for 10 min at 4 °C to extract proteins from the total lysate. Protein quantification was made, and samples (60 μg) were diluted in 4× NuPage LDS sample buffer (Invitrogen, Waltham, MA, USA, NP0007), heated at 80 °C for 5 min, and then separated by ExpressPlus™ PAGE Gel 12% acrylamide (GenScript, Piscataway, NJ, USA) with a Tris-MOPS running buffer (GenScript, Piscataway, NJ, USA) by electrophoresis. Proteins were then transferred to a PVDF membrane (Bio-Rad, Milan, Italy) using the TransBlot^®^ SE Semi-Dry Transfer Cell (Bio-Rad, Milan, Italy), and blots were blocked using the Odyssey Blocking Buffer (LI-COR Biosciences, Lincoln, NE, USA) for 1 h at room temperature. Membranes were incubated overnight with HO-1 (GTX101147, diluted 1:1000, GeneTex, Irvine, CA, USA) and β-actin (GTX109639, diluted 1:7000, GeneTex) primary antibodies. Goat anti-rabbit secondary antibody was used to detect blots (dil. 1:7000). Blots were scanned, and densitometric analysis was performed with the Odyssey Infrared Imaging System (LI-COR, Milan, Italy). Values were normalized to β-actin.

### 3.3. Docking Studies

The studied molecules were drawn using Marvin Sketch and minimized toward molecular mechanics by Merck molecular force field (MMFF94) optimization using the Marvin Sketch geometrical descriptors plugin [39]. The protonation states of the molecules were calculated assuming a neutral pH. The MMFF91 obtained 3D structures were subsequently optimized using the parameterized model number 3 (PM3) semi-empirical Hamiltonian implemented in the MOPAC package (vMOPAC2016) [40]. Docking calculations were made using AutoDock with the default docking parameters. At first, the point charges were assigned according to the AMBER14 force field and then corrected to mimic the less polar Gasteiger charges used to optimize the AutoDock scoring function. The setup was performed with the YASARA molecular modeling program [41]. The Lamarckian genetic algorithm (LGA) implemented in AutoDock was used for the calculations. The ligand-centered maps were generated by AutoGrid with a spacing of 0.375 Å and dimensions that encompass all atoms extending 5 Å from the surface of the ligand. All of the parameters were inserted at their default settings. In the docking tab, the macromolecule and ligand are selected, and GA parameters are set as ga_runs = 100, ga_pop_size = 150, ga_num_evals = 20,000,000, ga_num_generations = 27,000, ga_elitism = 1, ga_mutation_rate = 0.02, ga_crossover_rate = 0.8, ga_crossover_mode = two points, ga_cauchy_alpha = 0.0, ga_cauchy_beta = 1.0, number of generations for picking worst individual = 10. The X-ray crystal structures of the co-crystal HO-1/QC-80 (PDB ID: 3HOK) and of the crystal structure of the BCR-ABL kinase domain in complex with IM (PDB ID: 1IEP) were downloaded from the Protein Data Bank (www.rcsb.org, accessed on 18 April 2022). Only chain B and the prosthetic-heme group were retained from the crystal structures of the HO-1/QC-80 complex. Water molecules were also removed. All amino acid residues were kept rigid, whereas all single bonds of ligands were treated as fully flexible for both proteins. For all the molecules docked in HO-1, the best energies of binding for HO-1 were obtained after a molecular dynamics (MD) simulation. In all these cases, the ligands were docked into the selected binding sites of HO-1 at first; then the best pose was manually selected, and then the complex (ligand/HO-1) was minimized toward an MD simulation of 50 ns. At the end of the MD simulation, each ligand was extracted and re-docked into the binding site. The MD simulation was made in explicit water using YASARA as software. A 10 Å simulation cell around all atoms was used. AMBER 14 [42] force field was used for the simulation. Simulation temperature was set at 298 K, and the simulation cell was uniformly rescaled to reach a pressure of 1 bar; the pH was set at 7. The simulation was run for 50 ns, and single snapshots were recorded every 250 ps. BIOVIA Discovery Studio Visualizer 4.5 was used to generate all the figures in the paper.

### 3.4. Statistical Analysis

At least three independent experiments were performed for each analysis. The statistical significance of the differences between the experimental groups was determined by Fisher’s method for analysis of multiple comparisons, and the data are presented as mean ± SD.

## 4. Conclusions

The discovery of new-generation TKIs has revolutionized the treatment of patients with CML, although resistance toward BCR-ABL TKIs is still a relevant clinical problem [43,44]. The mechanisms behind the onset of resistance need to be further elucidated; however, literature data suggest that genomic amplification of BCR-ABL and HO-1 may be involved [8,27]. Based on the promising results derived from a previous series of IM-based hybrids that tackle both BCR-ABL and HO-1 [28], we performed structural modifications to develop new derivatives **1a**–**j**. In particular, we replaced the IM-based backbone with a NIL-like portion, maintaining the aryloxyalkylimidazole moiety. We chose NIL as the new backbone since this drug emerged as a new-generation TKI, 30-fold more potent than IM. Notably, the new compounds significantly inhibited BCR-ABL TK; in particular, three of them, **1a**, **1e**, **1g**, showed IC_50_ values in the nanomolar range. Moreover, we investigated their effects on the viability of NIL-resistant and -sensitive K562 cells. Interestingly, the new compounds significantly reduced the viability of K562-S cells, and two of them, **1g** and **1h**, showed cytotoxic properties similar to NIL and IM. Docking studies confirmed that these new compounds behave like TKIs showing the same interactions of IM and NIL at a molecular level. In addition, the MD simulation explained the only moderate inhibition toward HO-1 and the difference observed with respect to the previous series. Although the new compounds object of this work did not meet the expectations regarding HO-1 inhibition, we demonstrated for the first time that HO-1 is not involved in nilotinib-resistance mechanisms. The results of our study may provide new ideas to optimize the design of new TKIs endowed with potent activity.

## Figures and Tables

**Table 1:**
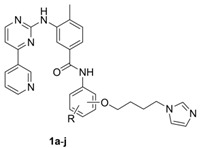
Inhibition potency of derivatives **1a**–**j** toward HO-1 and BCR-ABL kinase.

Compound	BCR-ABL IC_50_ (μM) ± SD	HO-1 IC_50_ (μM) ± SD
**1a**	0.109 ± 0.0016	44.79 ± 2.58
**1b**	4.19 ± 0.16	64.56 ± 0.69
**1c**	14.65 ± 0.283	149.38 ± 8.1
**1d**	16.16 ± 0.208	116.00 ± 5.24
**1e**	0.037 ± 0.0012	55.14 ± 0.71
**1f**	5.21 ± 0.224	100.24 ± 3.29
**1g**	0.077 ± 0.0036	50.95 ± 0.99
**1h**	0.77 ± 0.042	65.36 ± 1.39
**1i**	13.67 ± 0.139	83.44 ± 2.3
**1j**	5.45 ± 0.223	3059 ± 201
NIL	0.039 ^a^	–
IM	0.309 ^a^	–
**LS/0**	–	2.10 ± 0.3 ^b^
**LS1/71**	–	1.00 ± 0.05 ^b^

^a^ Data taken from Reference [31]. ^b^ Data taken from Reference [17].

**Table 2:** Docking calculated energies of binding for the selected compounds in BCR-ABL kinase.

Compound	IC_50_ (μM)	Calc.△*G*_B_ (kcal/mol)	Calc. *K*_i_ (μM)
**1a**	0.109	−9.59	0.092
**1b**	4.19	−7.74	2.10
**1c**	14.65	−6.33	22.78
**1d**	16.16	−6.45	18.67
**1e**	0.037	−10.29	0.028
**1f**	5.21	−7.15	5.68
**1g**	0.077	−9.26	0.161
**1h**	0.77	−7.64	2.48
**1i**	13.67	−6.19	28.94
**1j**	5.45	−7.05	6.75
**NIL**	0.039	−10.01	0.045
**IM**	0.309	−8.99	0.255

**Table 3:** Docking calculated energies of binding for the selected compounds in HO-1.

Compound	IC_50_ (μM)	Calc.△*G*_B_ (kcal/mol)	Calc. *K*_i_ (μM)
**1a**	44.79	−5.99	40.46
**1b**	64.56	−5.76	59.34
**1c**	149.38	−5.18	158.89
**1d**	116.00	−5.26	138.00
**1e**	55.14	−5.69	67.15
**1f**	100.24	−5.46	98.37
**1g**	50.95	−5.94	44.03
**1h**	65.36	−5.61	76.40
**1i**	83.44	−5.46	98.64
**1j**	3059	−4.05	1071.09
**QC-80** ^a^	2.1	−7.55	2.904

^a^ Data taken from Reference [33].

## Data Availability

Not applicable.

## Supplementary Materials

The following supporting information can be downloaded online: https://www.mdpi.com/article/10.3390/molecules27103220/s1. Synthesis and analytical data of intermediates **2****a**–**j**, **3a**–**j**, **4a**–**j**; Figures S1–S20: ^1^H and ^13^C NMR spectra of final compounds **1a**–**j**; Figures S21–S22: viability of K562-S and K562-R cell lines, Figure S23: HO-1 expression following NIL-resistance induction; Figures S24–S44: calculated interactions with BCR-ABL and HO-1. Refs. [17,45] are cited in Supplementary Material.

## Sample Availability

Not available.
