# Supplementary material for: Novel Tyrosine Kinase Inhibitors to Target Chronic Myeloid Leukemia
Source: Molecules. 2022 May 18;27(10):3220. doi: 10.3390/molecules27103220 (PMC9143943; doi:10.3390/molecules27103220)
Supplement: Supplementary file 1 [file molecules-27-03220-s001.zip › molecules-1668367 SI.pdf]

Supporting Information

Tyrosine kinase inhibitors to target chronic myeloid leukemia

Valeria Ciaffaglione [a,*,1], Valeria Consoli [a,1], Sebastiano Intagliata [a], Agostino Marrazzo [a], Giuseppe Romeo [a], Valeria Pittalà [a], Khaled Greish [b], Luca Vanella [a], Giuseppe Floresta [c], Antonio Rescifina [a], Loredana Salerno [a,*], Valeria Sorrenti [a]

[a] Department of Drug and Health Sciences, University of Catania, Catania – Italy

[b] Department of Molecular Medicine, College of Medicine and Medical Sciences, Princess Al-Jawhara Centre for Molecular Medicine, Arabian Gulf University, Manama, Kingdom of Bahrain

[c] Department of Analytical, Environmental and Forensic Sciences, King's College London, London, UK

Synthesis and analytical data of intermediates **2a**–**j**, **3a**–**j**, **4a**–**j**	**S2-S7**
Figures S1-S20: ^{1}H and ^{13}C NMR spectra of final compounds **1a**–**j**	**S8-S17**
Figures S21-S22: viability of K562-S and K562-R cell lines	**S18**
Figure S23: HO-1 expression following NIL-resistance induction	**S19**
Figures S24-S44: calculated interactions with BCR-ABL and HO-1	**S20–S40**
References	**S41**

*Synthesis of intermediates **2a–j**, **3a–j**, **4a–j***

*General procedure for the synthesis of bromobutoxy nitrobenzene derivatives (**2a–j**)*

The appropriate commercially available nitrophenol (6.8 mmol) was dissolved in acetone (9 mL); K_2CO_3 (20 mmol) and 1,4-dibromobutane (20 mmol) were added. The reaction mixture was stirred for 45 min under microwave irradiation in a sealed vial (90 °C, 150 W, 150 Psi). The solvent was evaporated under vacuum, water was added, and the resulted aqueous layer was extracted with EtOAc (3 × 50 mL). The organic layer was dried over anhydrous Na_2SO_4, filtered, and concentrated. The obtained residue was purified by column chromatography using a cyclohexane/EtOAc (9:1) mixture as eluent. Analytical and spectral data are reported only for unknown compounds **2d–j**. Compounds **2a–c** were previously reported in the literature [1].

*1-Bromo-4-(4-bromobutoxy)-2-nitrobenzene (**2d**)*

Yellow oil; yield 45 %. ^1H NMR (200 MHz, DMSO-d_6): δ 7.77 (d, J = 10 Hz, 1H, aromatic), 7.65–7.63 (m, 1H, aromatic), 7.24–7.18 (m, 1H, aromatic), 4.09 (t, J = 6 Hz, 2H, CH_2O), 3.61 (t, J = 6 Hz, 2H, CH_2Br), 2.02–1.77 (m, 4H, CH_2CH_2).

*2-Bromo-1-(4-bromobutoxy)-4-nitrobenzene (**2e**)*

Yellow solid; mp 30–32.3 °C; yield 30 %. ^1H NMR (200 MHz, DMSO-d_6): δ 8.44 (s, 1H, aromatic), 8.30–8.25 (m, 1H, aromatic), 7.33 (d, J = 10 Hz, 1H, aromatic), 4.27 (t, J = 6 Hz, 2H, CH_2O), 3.65 (t, J = 6 Hz, 2H, CH_2Br), 2.06–1.88 (m, 4H, CH_2CH_2).

*1-Bromo-2-(4-bromobutoxy)-4-nitrobenzene (**2f**)*

Yellow oil; yield 60 %. ^1H NMR (200 MHz, DMSO-d_6): δ 7.94–7.74 (m, 3H, aromatic), 4.26 (t, J = 6 Hz, 2H, CH_2O), 3.65 (t, J = 6 Hz, 2H, CH_2Br), 2.06–1.88 (m, 4H, CH_2CH_2).

*1-Bromo-3-(4-bromobutoxy)-5-nitrobenzene (**2g**)*

Orange oil; yield 60 %. ^1H NMR (200 MHz, DMSO-d_6): δ 7.96–7.94 (m, 1H, aromatic), 7.74–7.67 (m, 2H, aromatic), 4.17 (t, J = 6 Hz, 2H, CH_2O), 3.63 (t, J = 6 Hz, 2H, CH_2Br), 2.01–1.82 (m, 4H, CH_2CH_2).

*1-(4-Bromobutoxy)-3-nitro-5-(trifluoromethyl)benzene (**2h**)*

Yellow oil; yield 40 %. ^{1}H NMR (200 MHz, DMSO-d_6): δ 8.27–8.01 (m, 2H, aromatic), 7.79 (s, 1H, aromatic), 4.25 (t, *J* = 6 Hz, 2H, CH_2O), 3.64 (t, *J* = 6 Hz, 2H, CH_2Br), 2.03–1.85 (m, 4H, CH_2CH_2).

*4-Bromo-2-(4-bromobutoxy)-1-nitrobenzene (**2i**)*

Orange solid; mp 54.5–57.4 °C; yield 64.5 %. ^{1}H NMR (200 MHz, DMSO-d_6): δ 7.86 (d, *J* = 8 Hz, 1H, aromatic), 7.62 (s, 1H, aromatic), 7.36–7.31 (m, 1H, aromatic), 4.24 (t, *J* = 8 Hz, 2H, CH_2O), 3.62 (t, *J* = 8 Hz, 2H, CH_2Br), 2.00–1.81 (m, 4H, CH_2CH_2).

*1-(4-Bromobutoxy)-4-methyl-2-nitrobenzene (**2j**)*

Yellow oil; yield 30 %. ^{1}H NMR (200 MHz, DMSO-d_6): δ 7.69 (s, 1H, aromatic), 7.49–7.43 (m, 1H, aromatic), 7.27–7.23 (m, 1H, aromatic), 4.15 (t, *J* = 6 Hz, 2H, CH_2O), 3.61 (t, *J* = 6 Hz, 2H, CH_2Br), 2.30 (s, 3H. CH_3), 2.03–1.76 (m, 4H, CH_2CH_2).

*General procedure for the synthesis of nitrophenoxybutyl-1H-imidazole derivatives (**3a**–**j**)*

To a suspension of NaH (7.34 mmol) in THF (20 mL), 1*H*-imidazole (7.34 mmol) was added. After the disappearance of effervescence, the appropriate bromobutoxy nitrobenzene derivative **2a**–**j** (3.67 mmol) was added. The reaction mixture was left stirring for 24 h. The solvent was evaporated under vacuum, and the residue was suspended in NaOH 0.1 N aqueous solution (100 mL). The aqueous layer was extracted with EtOAc (2 × 50 mL) and the organic layer washed with brine. The organic layer was dried over anhydrous Na_2SO_4, filtered and concentrated. The obtained residue was purified by flash chromatography using an EtOAc/methanol (9.5:0.5) mixture as eluent. Analytical and spectral data are reported only for unknown compounds **3b–j**. Compound **3a** was previously reported in the literature [2].

*1-(4-(3-Nitrophenoxy)butyl)-1H-imidazole (**3b**)*

Yellow oil; yield 20 %. ^{1}H NMR (200 MHz, DMSO-d_6): δ 7.84–7.83 (m, 1H, imidazole), 7.71–7.53 (m, 3H, aromatic), 7.44–7.38 (m, 1H, aromatic), 7.21 (s, 1H, imidazole), 6.90 (s, 1H, imidazole), 4.14–4.01 (m, 4H, CH_2N, CH_2O), 1.95–1.80 (m, 2H, O-CH_2-CH_2-CH_2-CH_2-N), 1.73–1.60 (m, 2H, O-CH_2-CH_2-CH_2-CH_2-N).

*1-(4-(2-Nitrophenoxy)butyl)-1H-imidazole (**3c**)*

Yellow oil; yield 20.7 %. ^{1}H NMR (200 MHz, DMSO-d_6): δ 7.89–7.84 (m, 1H, aromatic), 7.69–7.60 (m, 2H, aromatic), 7.34 (d, J = 8 Hz, 1H, imidazole), 7.17–7.07 (m, 1H + 1H, aromatic + imidazole), 6.89 (s, 1H, imidazole), 4.16 (t, J = 6 Hz, 2H, CH_2O), 4.03 (t, J = 6 Hz, 2H, CH_2N), 1.94–1.79 (m, 2H, O-CH_2-CH_2-CH_2-CH_2-N), 1.71–1.58 (m, 2H, O-CH_2-CH_2-CH_2-CH_2-N).

*1-(4-(4-Bromo-3-nitrophenoxy)butyl)-1H-imidazole (**3d**)*

Yellow oil; yield 80 %. ^{1}H NMR (200 MHz, DMSO-d_6): δ 7.77 (d, J = 8 Hz, 1H, imidazole), 7.65–7.62 (m, 2H, aromatic), 7.22–7.16 (m, 1H + 1H, aromatic + imidazole), 6.89 (s, 1H, imidazole), 4.09–3.99 (m, 4H, CH_2N, CH_2O), 1.92–1.78 (m, 2H, O-CH_2-CH_2-CH_2-CH_2-N), 1.71–1.61 (m, 2H, O-CH_2-CH_2-CH_2-CH_2-N).

*1-(4-(2-Bromo-4-nitrophenoxy)butyl)-1H-imidazole (**3e**)*

Yellow oil; yield 23.3 %. ^{1}H NMR (200 MHz, DMSO-d_6): δ 8.44 (s, 1H, aromatic), 8.30–8.24 (m, 1H, imidazole), 7.66 (s, 1H, aromatic), 7.33–7.20 (m, 1H + 1H, aromatic + imidazole), 6.90 (s, 1H, imidazole), 4.23 (t, J = 5 Hz, 2H, CH_2O), 4.07 (t, J = 6 Hz, 2H, CH_2N), 1.98–1.84 (m, 2H, O-CH_2-CH_2-CH_2-CH_2-N), 1.77–1.67 (m, 2H, O-CH_2-CH_2-CH_2-CH_2-N).

*1-(4-(2-Bromo-5-nitrophenoxy)butyl)-1H-imidazole (**3f**)*

Yellow solid; mp 112.8–115.7 °C; yield 75 %. ^{1}H NMR (200 MHz, DMSO-d_6): δ 7.93–7.89 (m, 1H, imidazole), 7.82–7.73 (m, 2H, aromatic), 7.66 (s, 1H, aromatic), 7.20 (s, 1H, aromatic), 6.90 (s, 1H, imidazole), 4.22 (t, J = 6 Hz, 2H, CH_2O), 4.06 (t, J = 6 Hz, 2H, CH_2N), 1.98–1.85 (m, 2H, O-CH_2-CH_2-CH_2-CH_2-N), 1.77–1.67 (m, 2H, O-CH_2-CH_2-CH_2-CH_2-N).

*1-(4-(3-Bromo-5-nitrophenoxy)butyl)-1H-imidazole (**3g**)*

Brown oil; yield 62.5 %. ^{1}H NMR (200 MHz, DMSO-d_6): δ 7.96–7.94 (m, 1H, aromatic), 7.73–7.65 (m, 2H + 1H, aromatic + imidazole), 7.20–7.19 (m, 1H, imidazole), 6.89 (s, 1H, imidazole), 4.16–4.00 (m, 4H, CH_2N, CH_2O), 1.93–1.79 (m, 2H, O-CH_2-CH_2-CH_2-CH_2-N), 1.72–1.59 (m, 2H, O-CH_2-CH_2-CH_2-CH_2-N).

*1-(4-(3-Nitro-5-(trifluoromethyl)phenoxy)butyl)-1H-imidazole (**3h**)*

White solid; mp 73.7–77.6 °C; yield 93 %. ^{1}H NMR (200 MHz, DMSO-d_6): δ 8.05–8.00 (m, 1H + 1H, aromatic + imidazole), 7.77 (s, 1H, aromatic), 7.67 (s, 1H, aromatic), 7.21 (s, 1H, imidazole), 6.90 (s, 1H, imidazole), 4.21 (t, J = 6 Hz, 2H, CH_2O), 4.05 (t, J = 6 Hz, 2H, CH_2N), 1.92–1.85 (m, 2H, O-CH_2-CH_2-CH_2-CH_2-N), 1.72–1.65 (m, 2H, O-CH_2-CH_2-CH_2-CH_2-N).

*1-(4-(5-Bromo-2-nitrophenoxy)butyl)-1H-imidazole (**3i**)*

Yellow solid; mp 91.6–95.3 °C; yield 78 %. ^{1}H NMR (200 MHz, DMSO-d_6): δ 7.86 (d, J = 10 Hz, 1H, imidazole), 7.64–7.60 (m, 2H, aromatic), 7.36–7.30 (m, 1H, aromatic), 7.17 (s, 1H, imidazole), 6.89 (s, 1H, imidazole), 4.20 (t, J = 6 Hz, 2H, CH_2O), 4.03 (t, J = 6 Hz, 2H, CH_2N), 1.89–1.78 (m, 2H, O-CH_2-CH_2-CH_2-CH_2-N), 1.70–1.60 (m, 2H, O-CH_2-CH_2-CH_2-CH_2-N).

*1-(4-(4-Methyl-2-nitrophenoxy)butyl)-1H-imidazole (**3j**)*

Yellow solid; mp 70.2–74.5 °C; yield 86 %. ^{1}H NMR (200 MHz, DMSO-d_6): δ 7.69–7.63 (m, 1H + 1H, aromatic + imidazole), 7.48–7.43 (m, 1H, aromatic), 7.25–7.16 (m, 1H + 1H, aromatic + imidazole), 6.89 (s, 1H, imidazole), 4.15–3.99 (m, 4H, CH_2N, CH_2O), 2.30 (s, 3H, CH_3), 1.93–1.78 (m, 2H, O-CH_2-CH_2-CH_2-CH_2-N), 1.70–1.56 (m, 2H, O-CH_2-CH_2-CH_2-CH_2-N).

*General procedure for the synthesis of (1H-imidazol-1-yl)butoxy aniline derivatives (**4a**–**j**)*

To a solution of the appropriate nitrophenoxybutyl-1*H*-imidazole derivative (**3a**–**j**) (1.24 mmol) in methanol/water (1:1, 20 mL), iron powder (8.68 mmol) and NH_4Cl (8.68 mmol) were added. The reaction mixture was left stirring at 65 °C for 3 hours. After cooling, the mixture was filtered using a Celite pad. The solvent was evaporated under vacuum, and $NaHCO_3$ aqueous solution (100 mL) was added. The aqueous layer was extracted with EtOAc (3 × 50 mL), and the organic layer was washed with brine. The organic layer was dried over anhydrous Na_2SO_4, filtered, and concentrated to afford the amine.

*4-(4-(1H-Imidazol-1-yl)butoxy)aniline (**4a**)*

Brown oil; yield 92 %. ^{1}H NMR (200 MHz, DMSO-d_6): δ 7.63 (s, 1H, imidazole), 7.18 (s, 1H, aromatic), 6.88 (s, 1H, imidazole), 6.66–6.59 (m, 1H + 1H, aromatic + imidazole), 6.52–6.46 (m, 2H, aromatic), 4.60 (s, 2H, NH_2), 4.00 (t, J = 6 Hz, 2H, CH_2O), 3.80 (t, J = 6 Hz,

2H, CH_2N), 1.89–1.75 (m, 2H, O-CH_2-CH_2-CH_2-CH_2-N), 1.63–1.50 (m, 2H, O-CH_2-CH_2-CH_2-CH_2-N).

*3-(4-(1H-Imidazol-1-yl)butoxy)aniline (**4b**)*

Brown oil; yield 93 %. ^{1}H NMR (200 MHz, DMSO-d_6): 7.64 (s, 1H, imidazole), 7.19 (s, 1H, imidazole), 6.91–6.83 (m, 2H, aromatic), 6.14–6.03 (m, 2H + 1H, aromatic + imidazole), 5.04 (s, 2H, NH_2), 4.01 (t, J = 6 Hz, 2H, CH_2O), 3.84 (t, J = 6 Hz, 2H, CH_2N), 1.86–1.79 (m, 2H, O-CH_2-CH_2-CH_2-CH_2-N), 1.63–1.55 (m, 2H, O-CH_2-CH_2-CH_2-CH_2-N).

*2-(4-(1H-Imidazol-1-yl)butoxy)aniline (**4c**)*

Brown oil; yield 98 %. ^{1}H NMR (200 MHz, DMSO-d_6): 7.68 (s, 1H, imidazole), 7.21 (s, 1H, aromatic), 6.90 (s, 1H, imidazole), 6.77–6.60 (m, 2H + 1H, aromatic + imidazole), 6.53–6.44 (m, 1H, aromatic), 4.06–3.89 (m, 4H, CH_2N, CH_2O), 1.97–1.82 (m, 2H, O-CH_2-CH_2-CH_2-CH_2-N), 1.71–1.58 (m, 2H, O-CH_2-CH_2-CH_2-CH_2-N).

*5-(4-(1H-Imidazol-1-yl)butoxy)-2-bromoaniline (**4d**)*

Yellow oil; yield 87 %. ^{1}H NMR (200 MHz, DMSO-d_6): δ 7.64 (s, 1H, aromatic), 7.19–7.15 (m, 2H, aromatic), 6.89 (s, 1H, imidazole), 6.36 (d, J = 2 Hz, 1H, imidazole), 6.11–6.06 (m, 1H, imidazole), 5.26 (s, 2H, NH_2), 4.01 (t, J = 6 Hz, 2H, CH_2O), 3.85 (t, J = 6 Hz, 2H, CH_2N), 1.86–1.75 (m, 2H, O-CH_2-CH_2-CH_2-CH_2-N), 1.66–1.56 (m, 2H, O-CH_2-CH_2-CH_2-CH_2-N).

*4-(4-(1H-Imidazol-1-yl)butoxy)-3-bromoaniline (**4e**)*

Yellow oil; yield 85.5 %. ^{1}H NMR (200 MHz, DMSO-d_6): δ 7.64 (s, 1H, imidazole), 7.18 (s, 1H, aromatic), 6.88–6.78 (m, 2H + 1H, aromatic + imidazole), 6.53–6.48 (m, 1H, imidazole), 4.92 (s, 2H, NH_2), 4.03 (t, J = 6 Hz, 2H, CH_2N), 3.86 (t, J = 6 Hz, 2H, CH_2O), 1.94–1.90 (m, 2H, O-CH_2-CH_2-CH_2-CH_2-N), 1.66–1.52 (m, 2H, O-CH_2-CH_2-CH_2-CH_2-N).

*3-(4-(1H-Imidazol-1-yl)butoxy)-4-bromoaniline (**4f**)*

Brown solid; mp 104–106.5 °C; yield 94.5 %. ^{1}H NMR (200 MHz, DMSO-d_6): δ 7.65 (s, 1H, imidazole), 7.19–7.08 (m, 1H + 1H, aromatic + imidazole), 6.90 (s, 1H, aromatic), 6.28 (s, 1H, imidazole), 6.12–6.07 (m, 1H, aromatic), 5.28 (s, 2H, NH_2), 4.05 (t, J = 6 Hz, 2H,

CH_2O), 3.91 (t, *J* = 6 Hz, 2H, CH_2N), 1.95–1.81 (m, 2H, O-CH_2-CH_2-CH_2-CH_2-N), 1.70–1.60 (m, 2H, O-CH_2-CH_2-CH_2-CH_2-N).

*3-(4-(1H-Imidazol-1-yl)butoxy)-5-bromoaniline (**4g**)*

Brown oil; yield 92.4 %. ^{1}H NMR (200 MHz, DMSO-d_6): δ 7.64 (s, 1H, imidazole), 7.19 (s, 1H, aromatic), 6.89 (s, 1H, imidazole), 6.32 (s, 1H, imidazole), 6.23–6.21 (m, 1H, aromatic), 6.08–6.06 (m, 1H, aromatic), 5.40 (s, 2H, NH_2), 4.01 (t, *J* = 8 Hz, 2H, CH_2O), 3.86 (t, *J* = 8 Hz, 2H, CH_2N), 1.88–1.74 (m, 2H, O-CH_2-CH_2-CH_2-CH_2-N), 1.65–1.51 (m, 2H, O-CH_2-CH_2-CH_2-CH_2-N).

*3-(4-(1H-Imidazol-1-yl)butoxy)-5-(trifluoromethyl)aniline (**4h**)*

Brown oil; yield 90 %. ^{1}H NMR (200 MHz, DMSO-d_6): δ 7.65 (s, 1H, imidazole), 7.19 (s, 1H, imidazole), 6.89 (s, 1H, imidazole), 6.44 (s, 1H, aromatic), 6.33–6.30 (s, 2H, aromatic), 5.59 (s, 2H, NH_2), 4.05–3.89 (m, 4H, CH_2O, CH_2N), 1.88–1.80 (m, 2H, O-CH_2-CH_2-CH_2-CH_2-N), 1.65–1.57 (m, 2H, O-CH_2-CH_2-CH_2-CH_2-N).

*2-(4-(1H-Imidazol-1-yl)butoxy)-4-bromoaniline (**4i**)*

Brown oil; yield 90 %. ^{1}H NMR (200 MHz, DMSO-d_6): δ 7.65 (s, 1H, imidazole), 7.20 (s, 1H, imidazole), 6.89–6.78 (m, 2H + 1H, aromatic + imidazole), 6.58–6.54 (m, 1H, aromatic), 4.88 (s, 2H, NH_2), 4.05–3.91 (m, 4H, CH_2O, CH_2N), 1.92–1.81 (m, 2H, O-CH_2-CH_2-CH_2-CH_2-N), 1.70–1.60 (m, 2H, O-CH_2-CH_2-CH_2-CH_2-N).

*2-(4-(1H-Imidazol-1-yl)butoxy)-5-methylaniline (**4j**)*

Brown solid; mp 57.1–61.2 °C; yield 98 %. ^{1}H NMR (200 MHz, DMSO-d_6): δ 7.64 (s, 1H, aromatic), 7.20 (s, 1H, imidazole), 6.89 (s, 1H, imidazole), 6.62 (d, *J* = 8 Hz, 1H, aromatic), 6.44 (s, 1H, imidazole), 6.31–6.26 (m, 1H, aromatic), 4.59 (s, 2H, NH_2), 4.01 (t, *J* = 6 Hz, 2H, CH_2O), 3.87 (t, *J* = 6 Hz, 2H, CH_2N), 2.11 (s, 3H, CH_3), 1.95–1.80 (m, 2H, O-CH_2-CH_2-CH_2-CH_2-N), 1.69–1.59 (m, 2H, O-CH_2-CH_2-CH_2-CH_2-N).

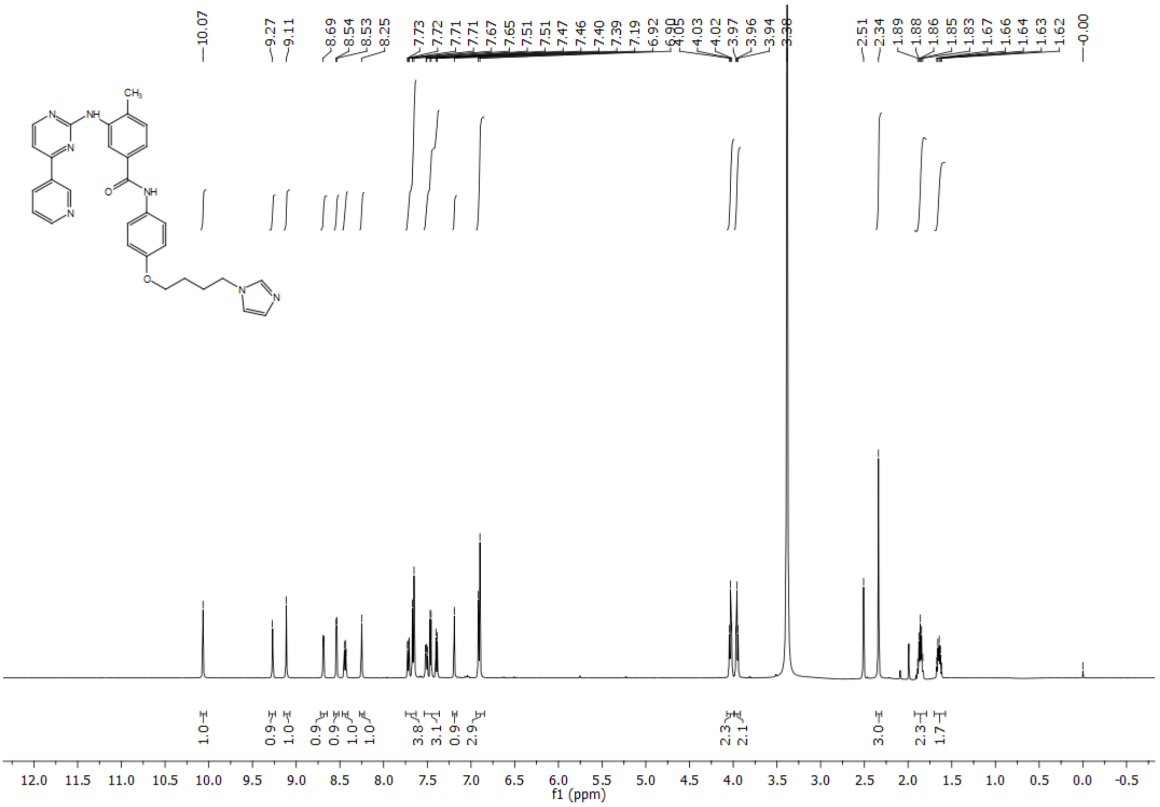

Figure S1. ^{1}H NMR (500 MHz, DMSO-d_6) of compound **1a**.

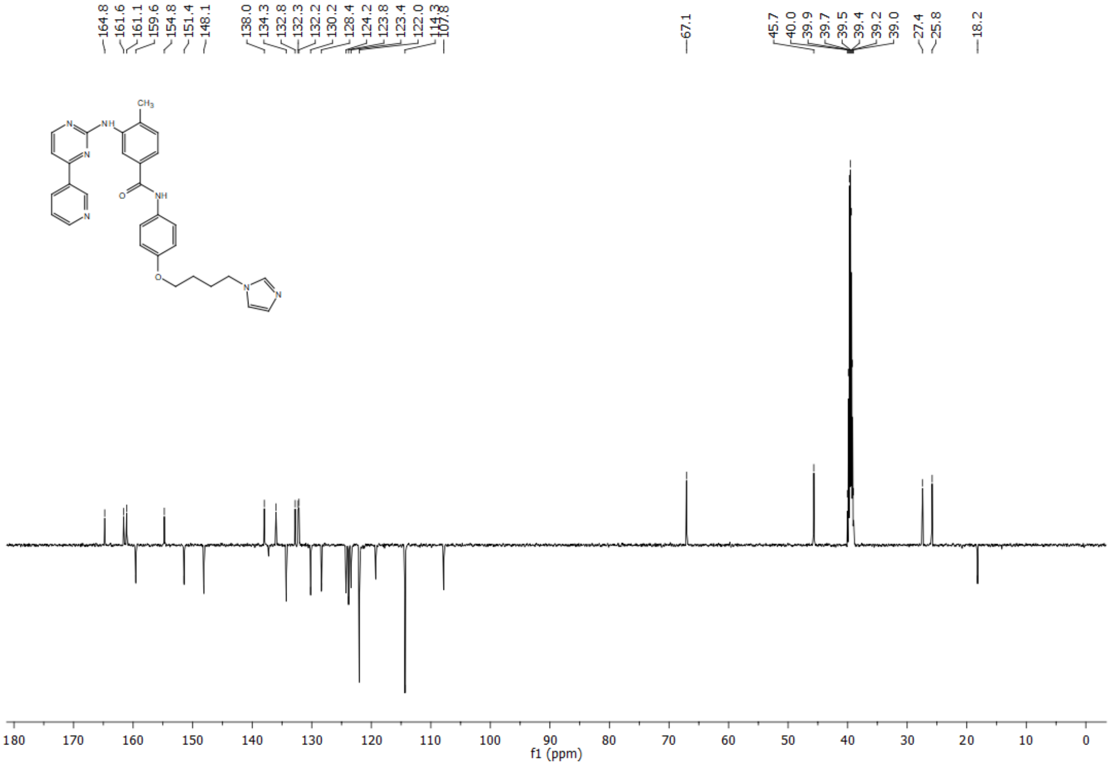

Figure S2. ^{13}C NMR (125 MHz, DMSO-d_6) of compound **1a**.

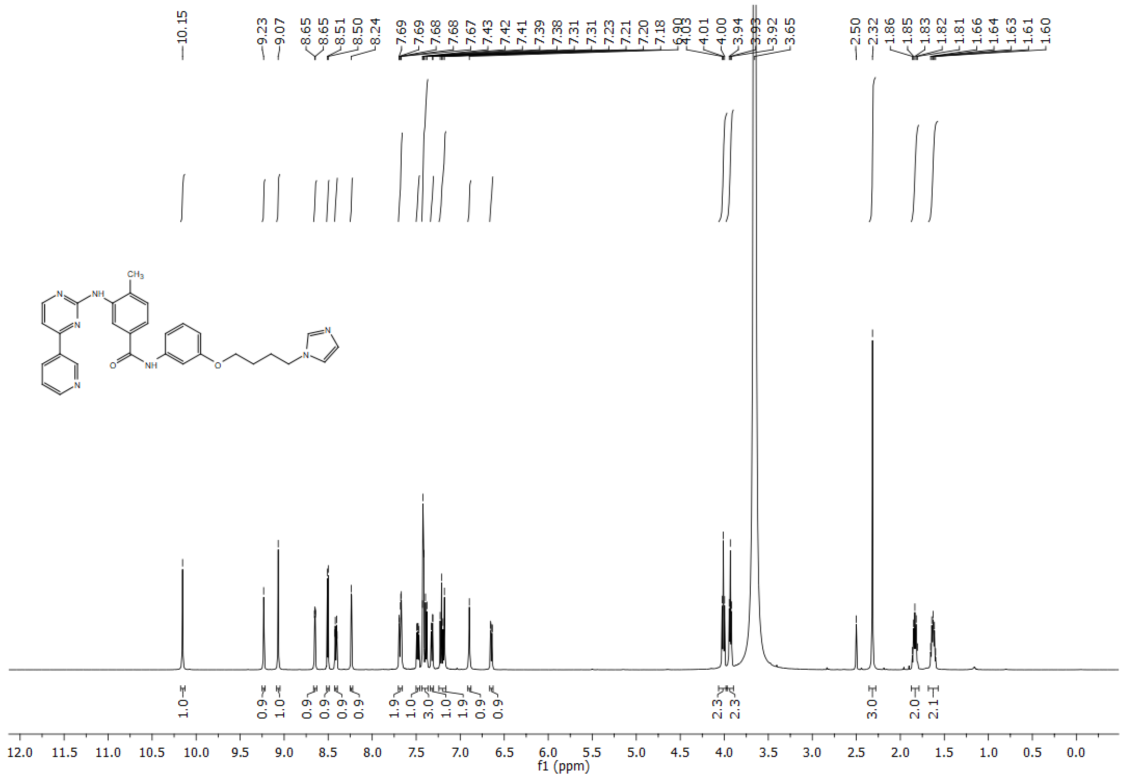

Figure ^{1}H NMR (500 MHz, DMSO-d_6) of compound **1b**.

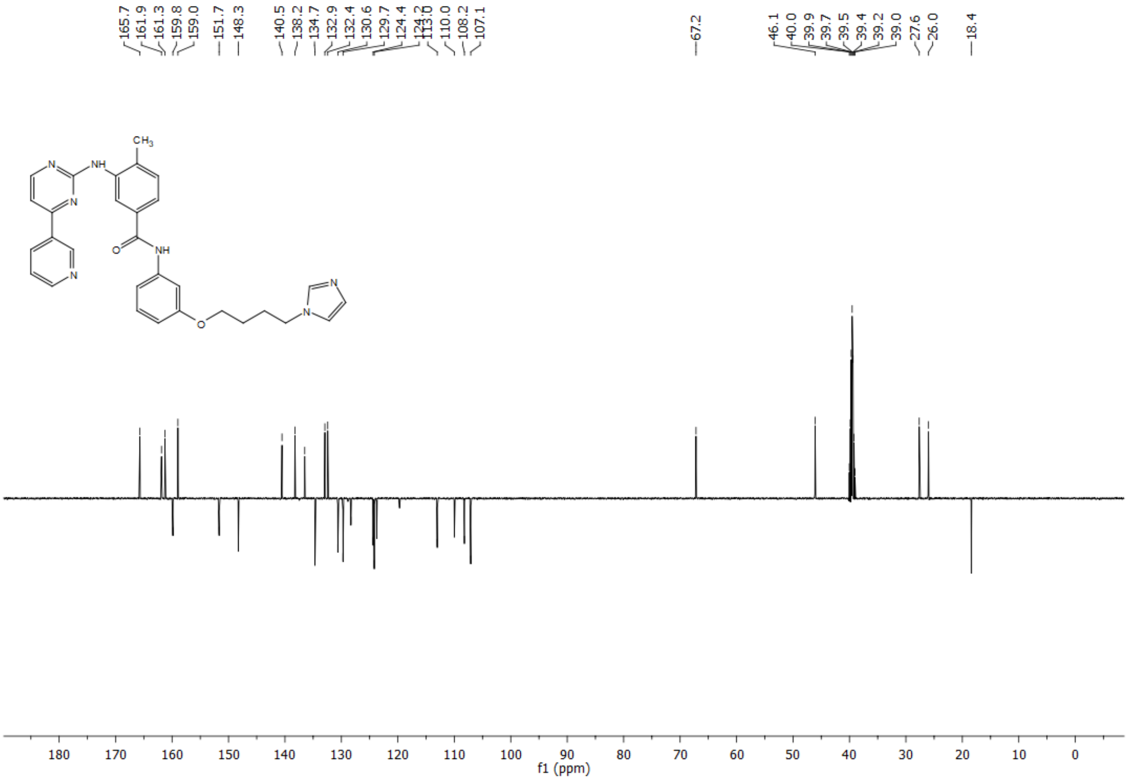

Figure S4. ^{13}C NMR (125 MHz, DMSO-d_6) of compound **1b**.

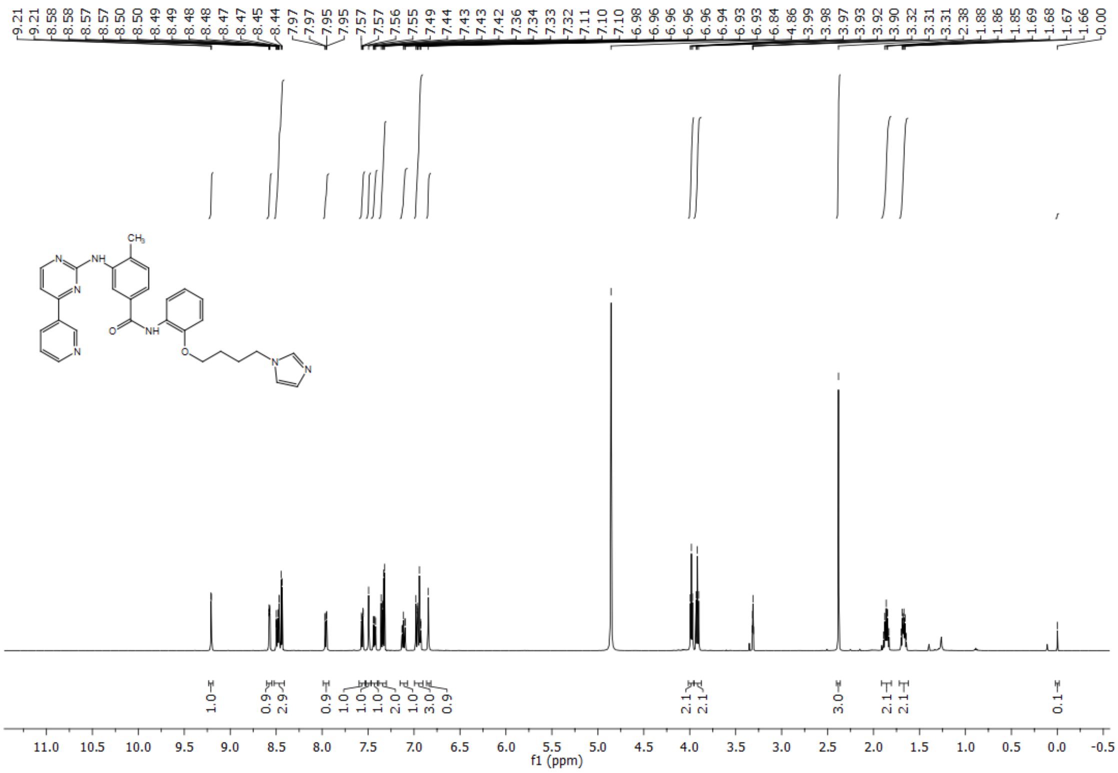

Figure S5. ^{1}H NMR (500 MHz, CD_3OD) of compound **1c**.

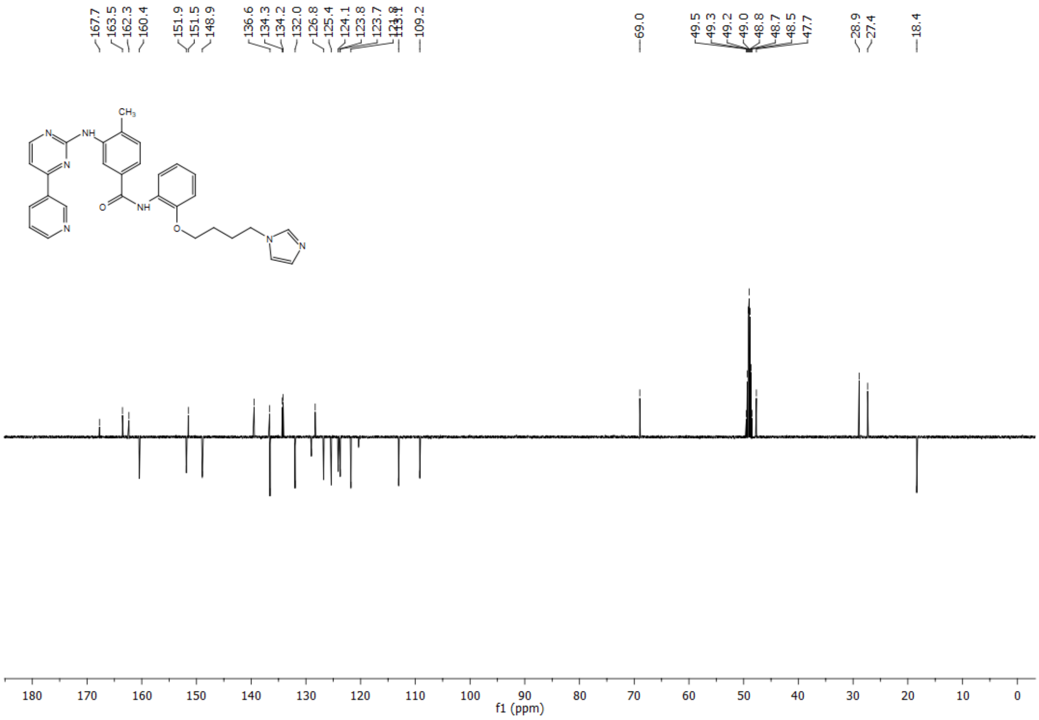

Figure S6. ^{13}C NMR (125 MHz, CD_3OD) of compound **1c**.

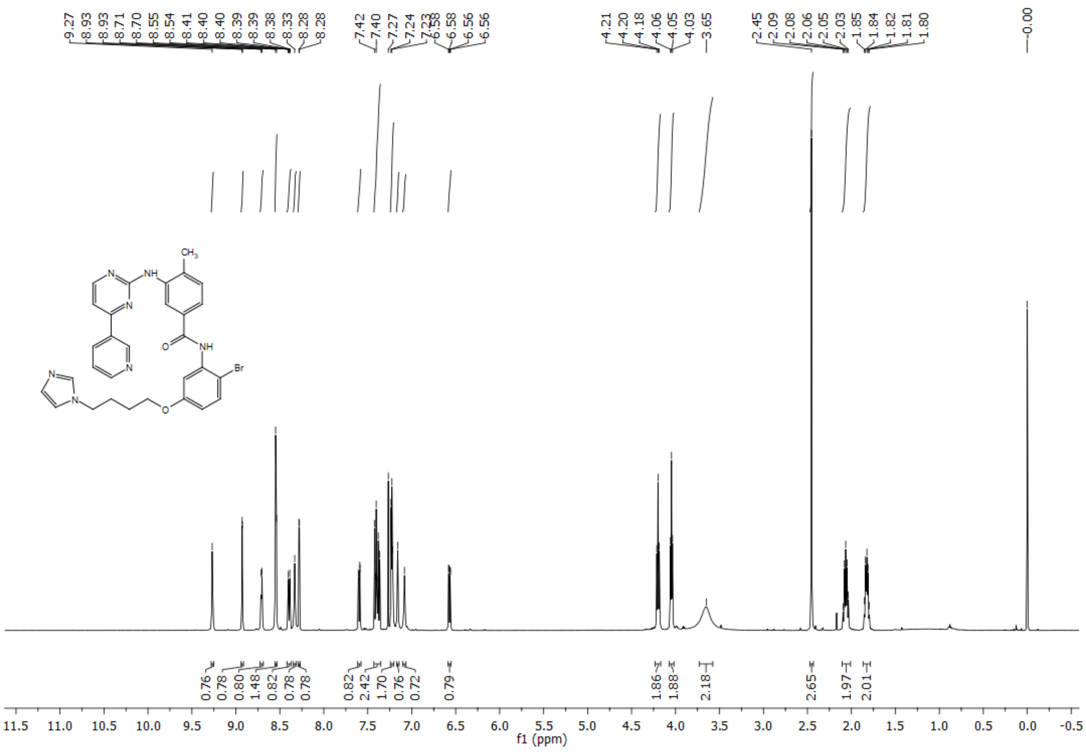

Figure S7. ^{1}H NMR (500 MHz, $CDCl_3$) of compound **1d**.

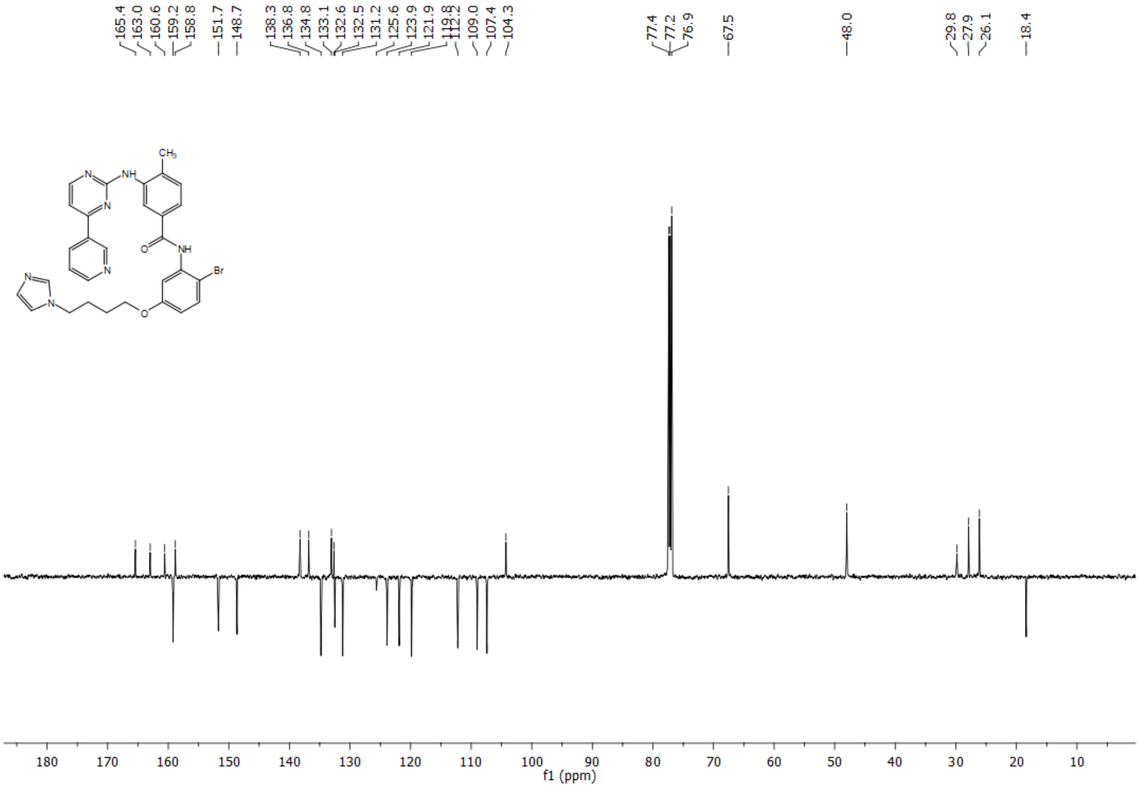

Figure S8. ^{13}C NMR (125 MHz, $CDCl_3$) of compound **1d**.

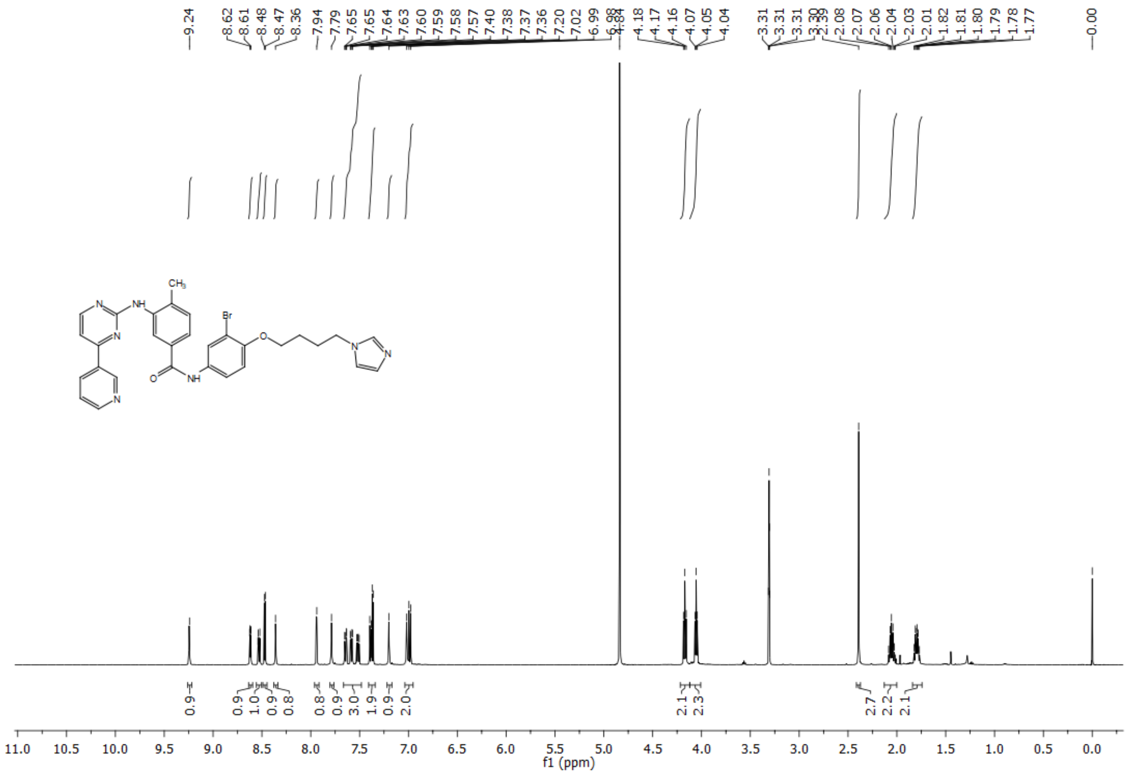

Figure S9. ^{1}H NMR (500 MHz, CD_3OD) of compound **1e**.

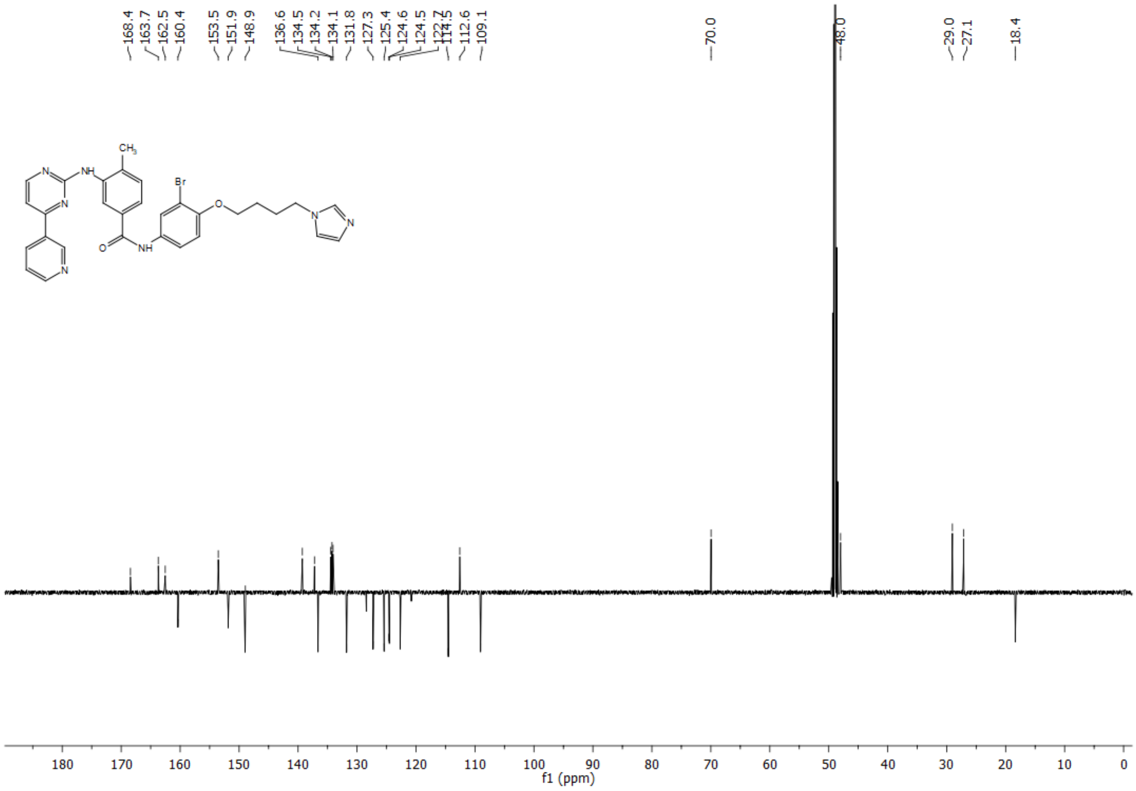

Figure S10. ^{13}C NMR (125 MHz, CD_3OD) of compound **1e**.

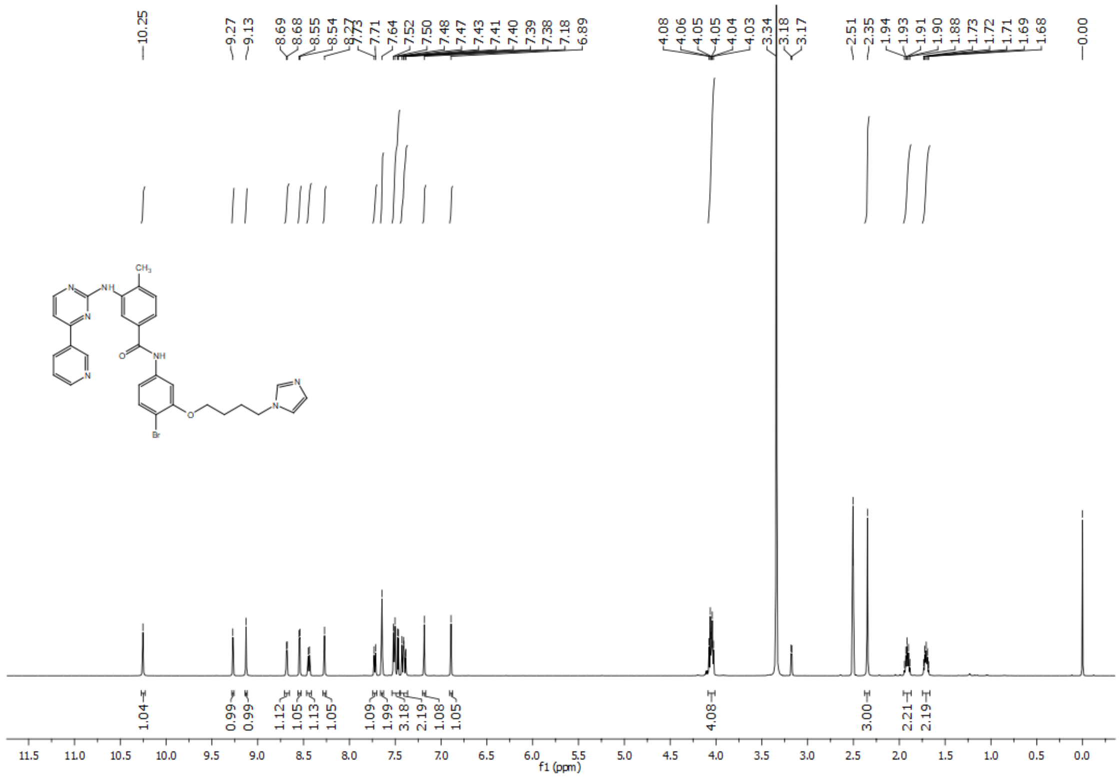

Figure S11. ^{1}H NMR (500 MHz, DMSO-d_6) of compound **1f**.

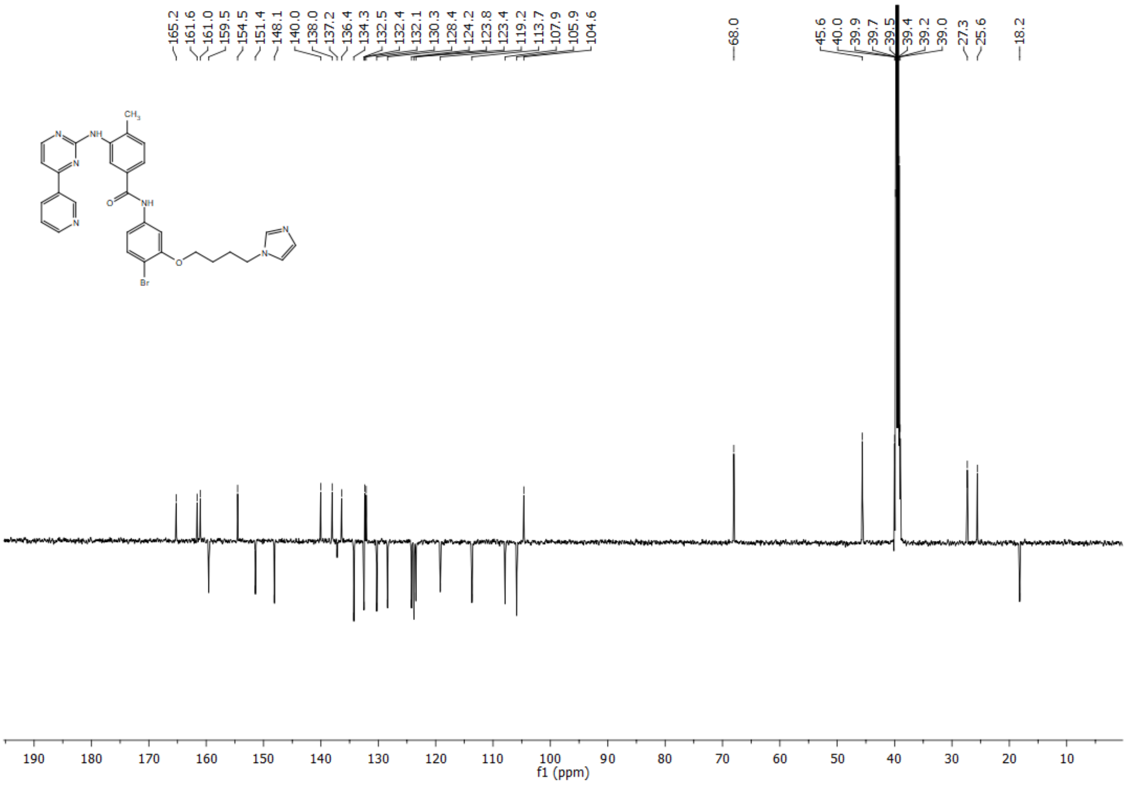

Figure S12. ^{13}C NMR (125 MHz, DMSO-d_6) of compound **1f**.

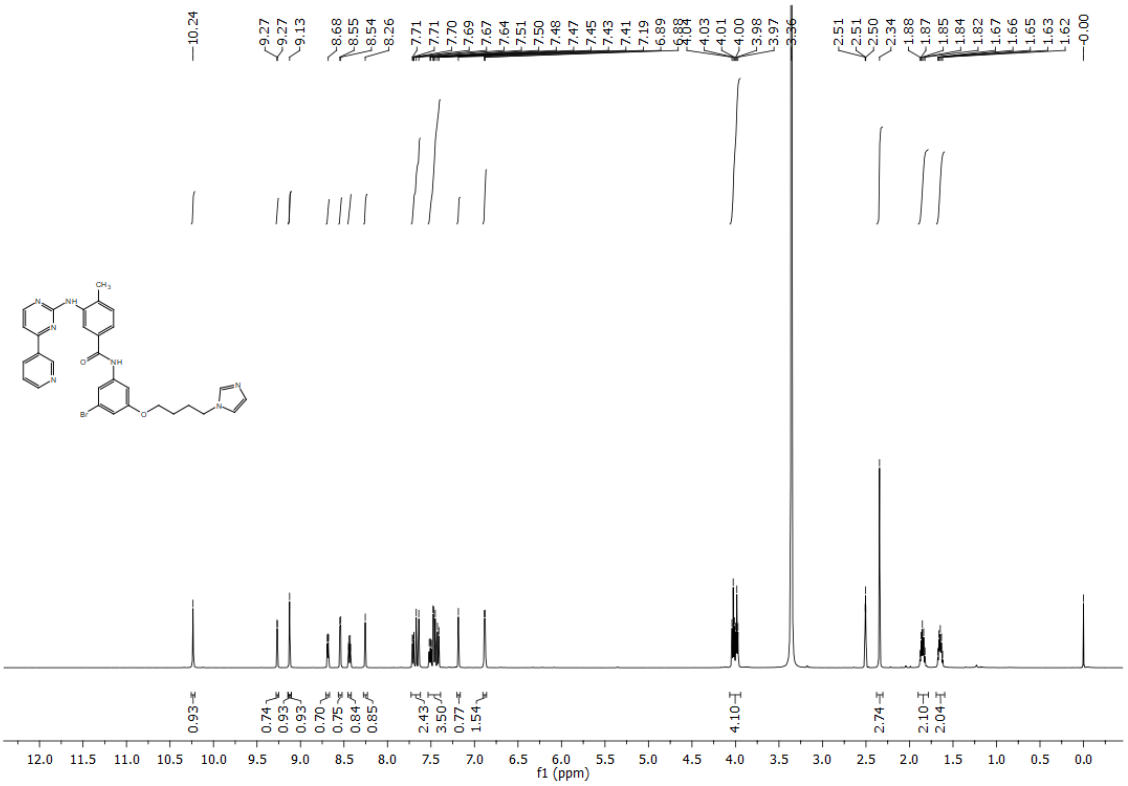

Figure S13. ^{1}H NMR (500 MHz, DMSO-d_6) of compound **1g**.

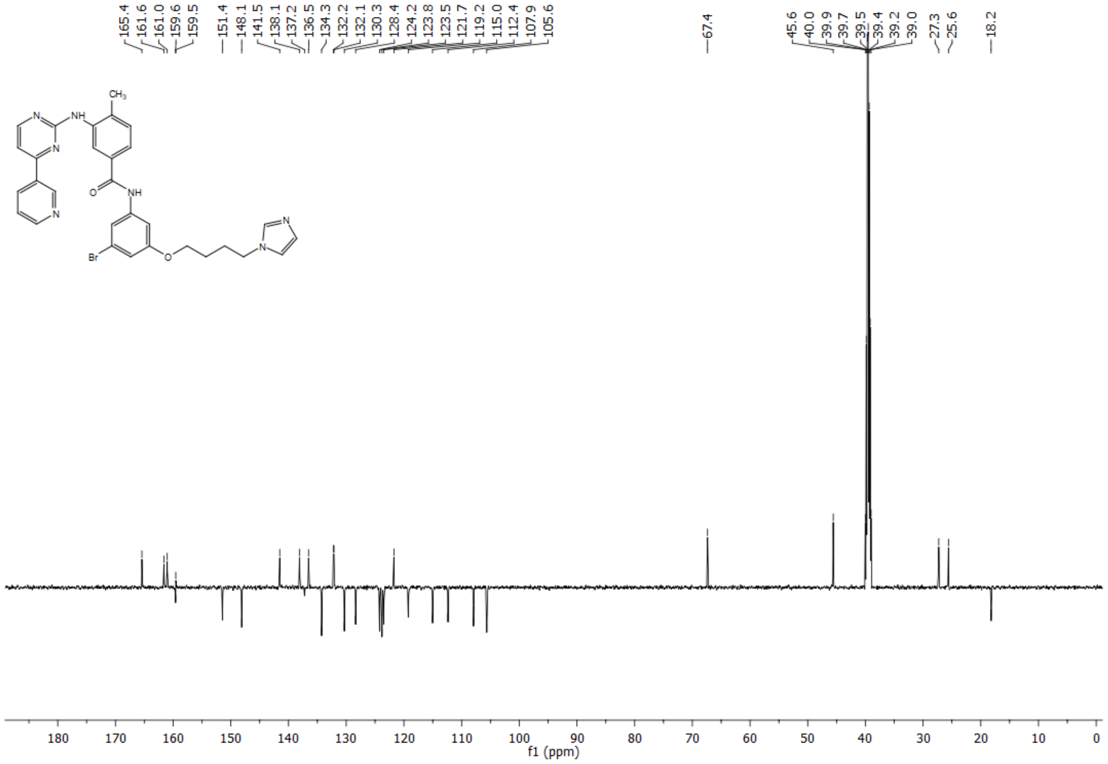

Figure S14. ^{13}C NMR (125 MHz, DMSO-d_6) of compound **1g**.

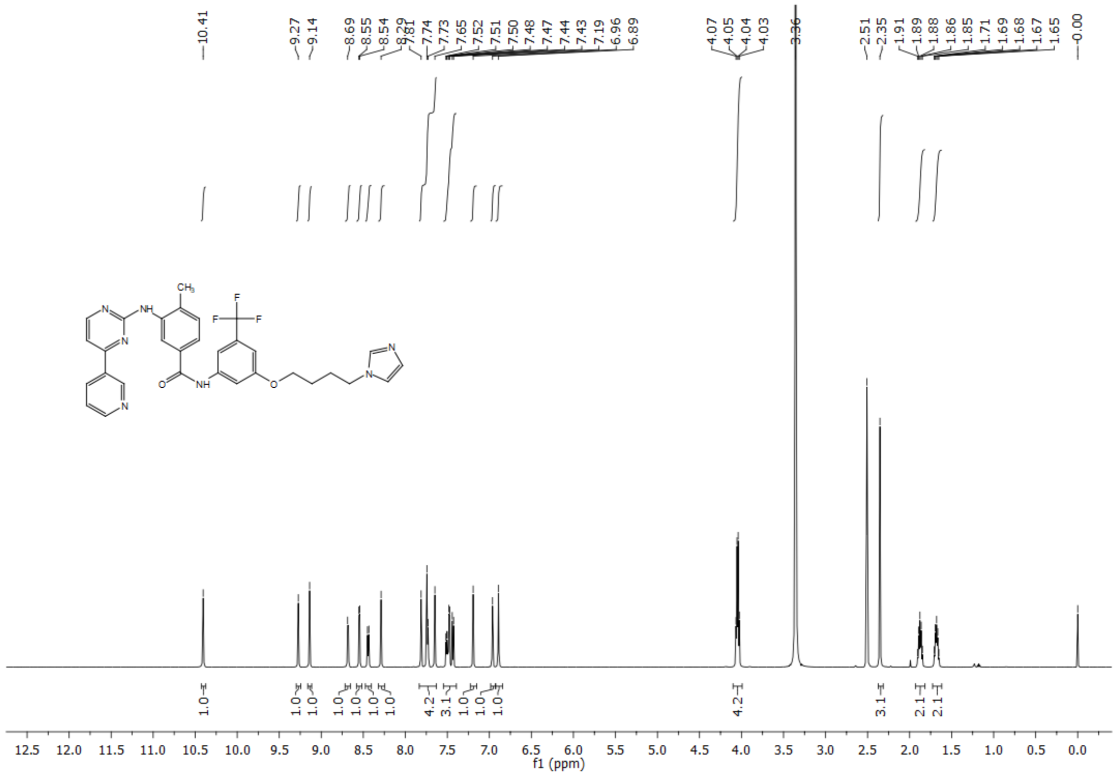

Figure S15. ^{1}H NMR (500 MHz, DMSO-d_6) of compound **1h**.

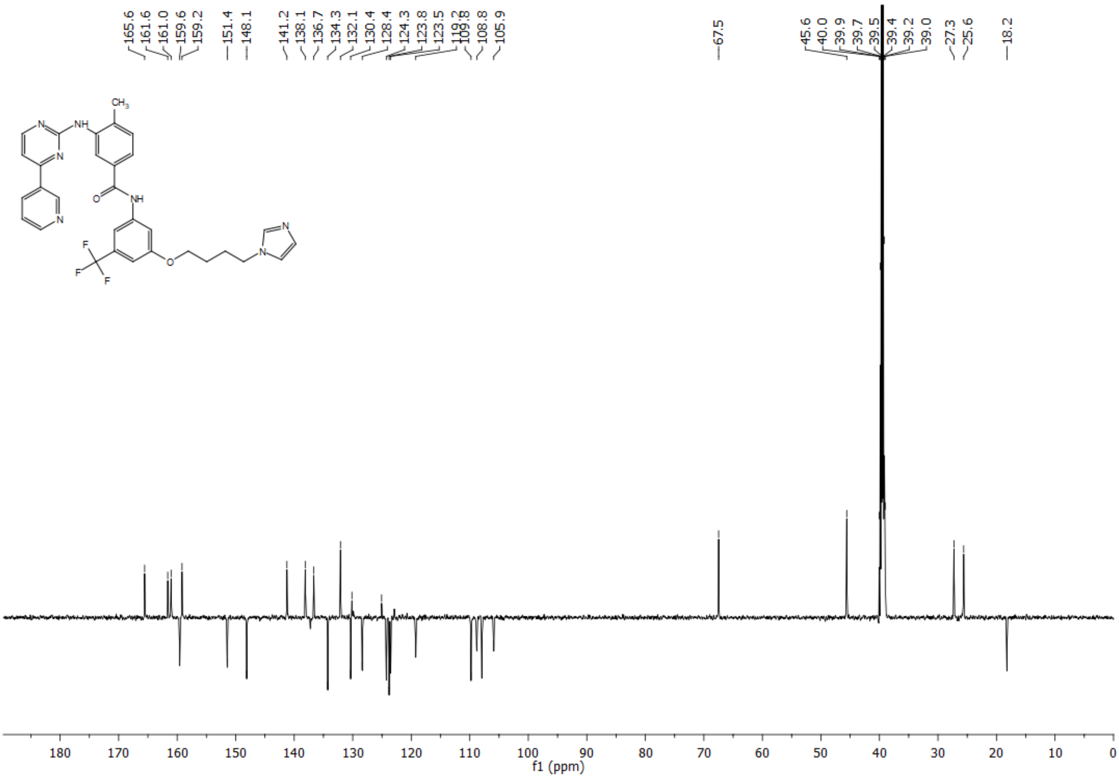

Figure S16. ^{13}C NMR (125 MHz, DMSO-d_6) of compound **1h**.

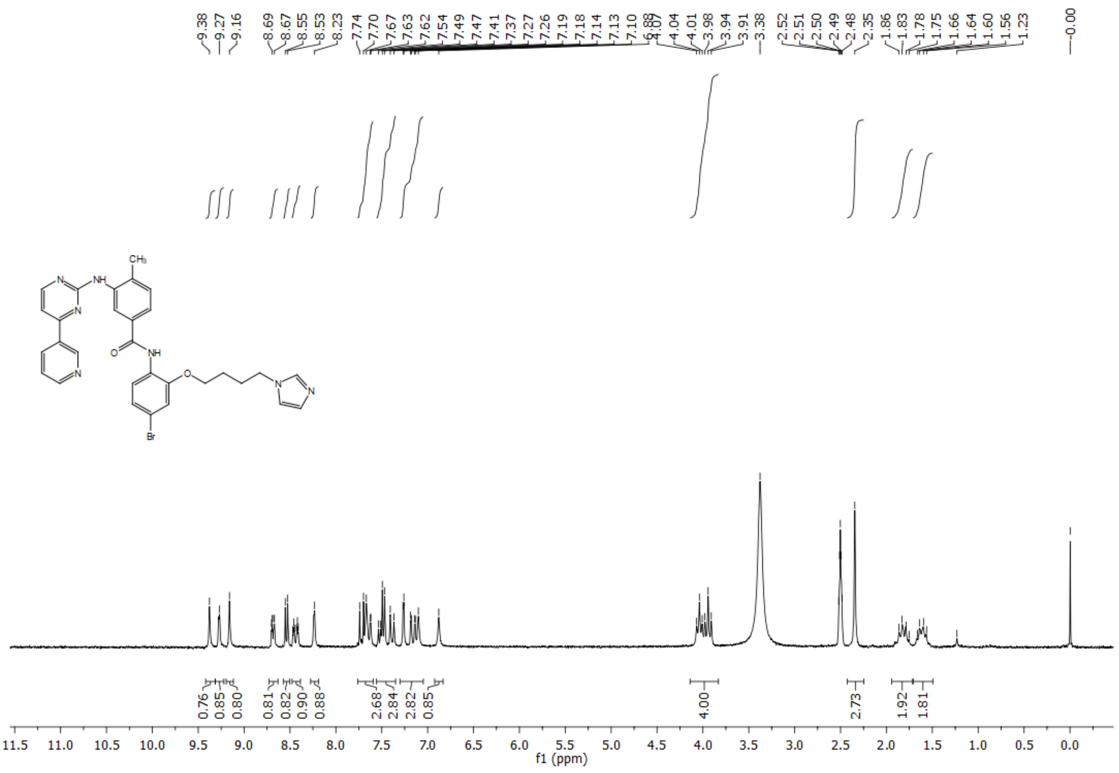

Figure S17. ^{1}H NMR (200 MHz, DMSO-d_6) of compound **1i**.

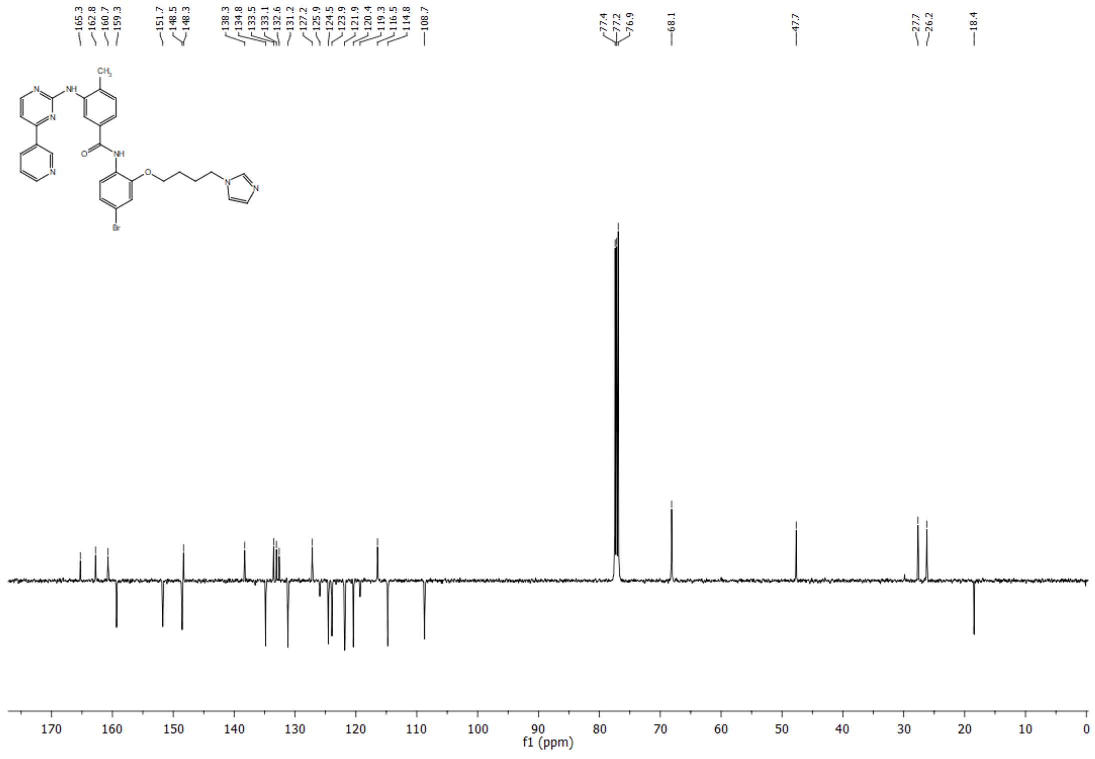

Figure S18. ^{13}C NMR (125 MHz, $CDCl_3$) of compound **1i**.

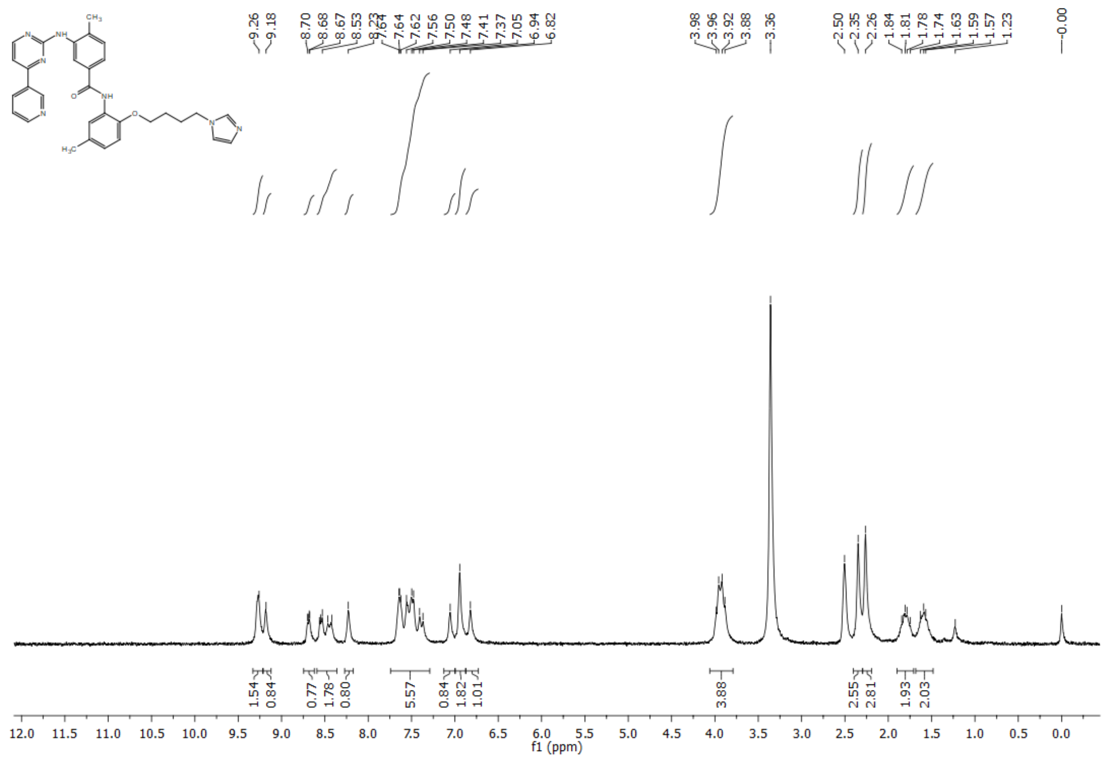

Figure S19. ^{1}H NMR (200 MHz, DMSO-d_6) of compound **1j**.

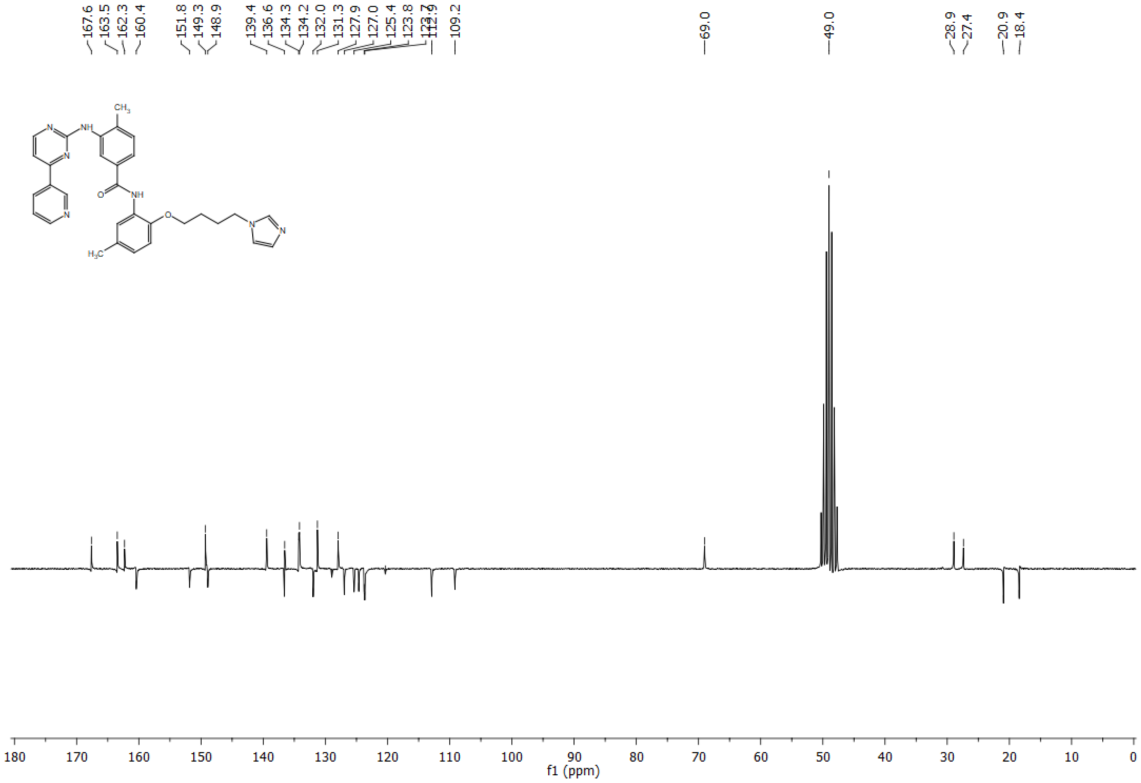

Figure S20. ^{13}C NMR (50 MHz, CD_3OD) of compound **1j**.

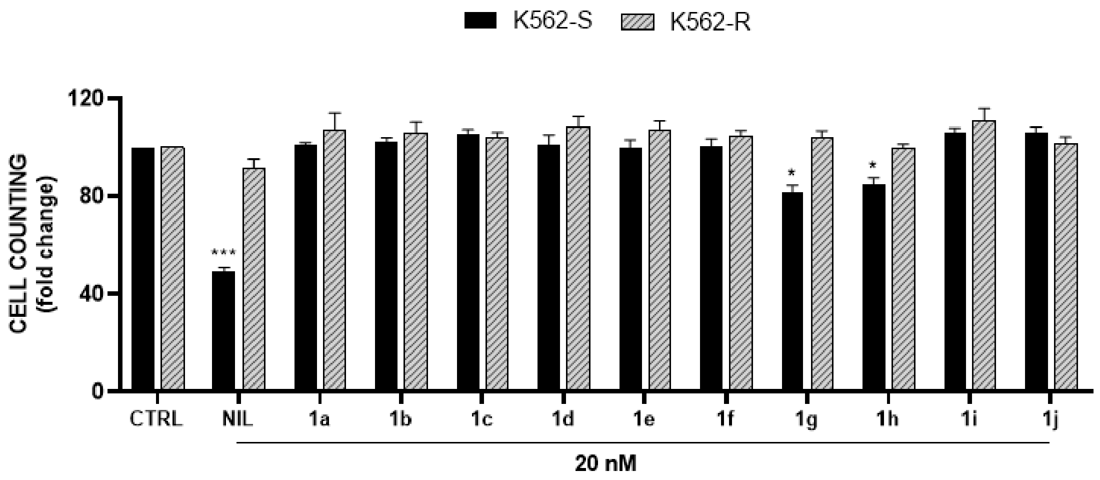

Figure S21. Effect of hybrids on cell counting of K562-S and K562-R cell lines at 20 nM. ***p≤ 0,0005 vs CTRL, **p≤ 0,005 vs CTRL, *p≤ 0,05 vs CTRL.

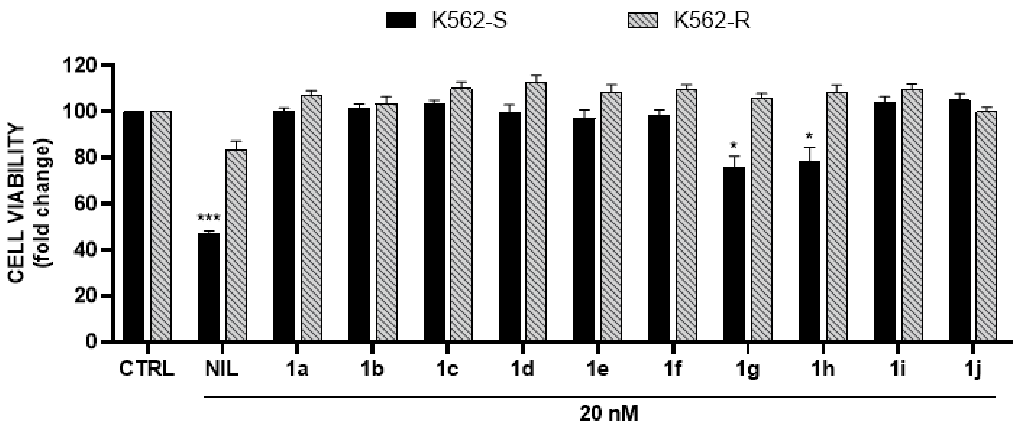

Figure S22. Evaluation of hybrids effect on cell viability of K562-R and K562-S. ***p≤ 0,0005 vs CTRL, *p≤ 0,05 vs CTRL.

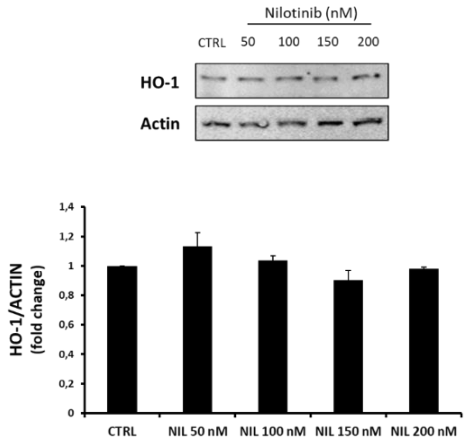

Figure S23. HO-1 expression following NIL-resistance induction.

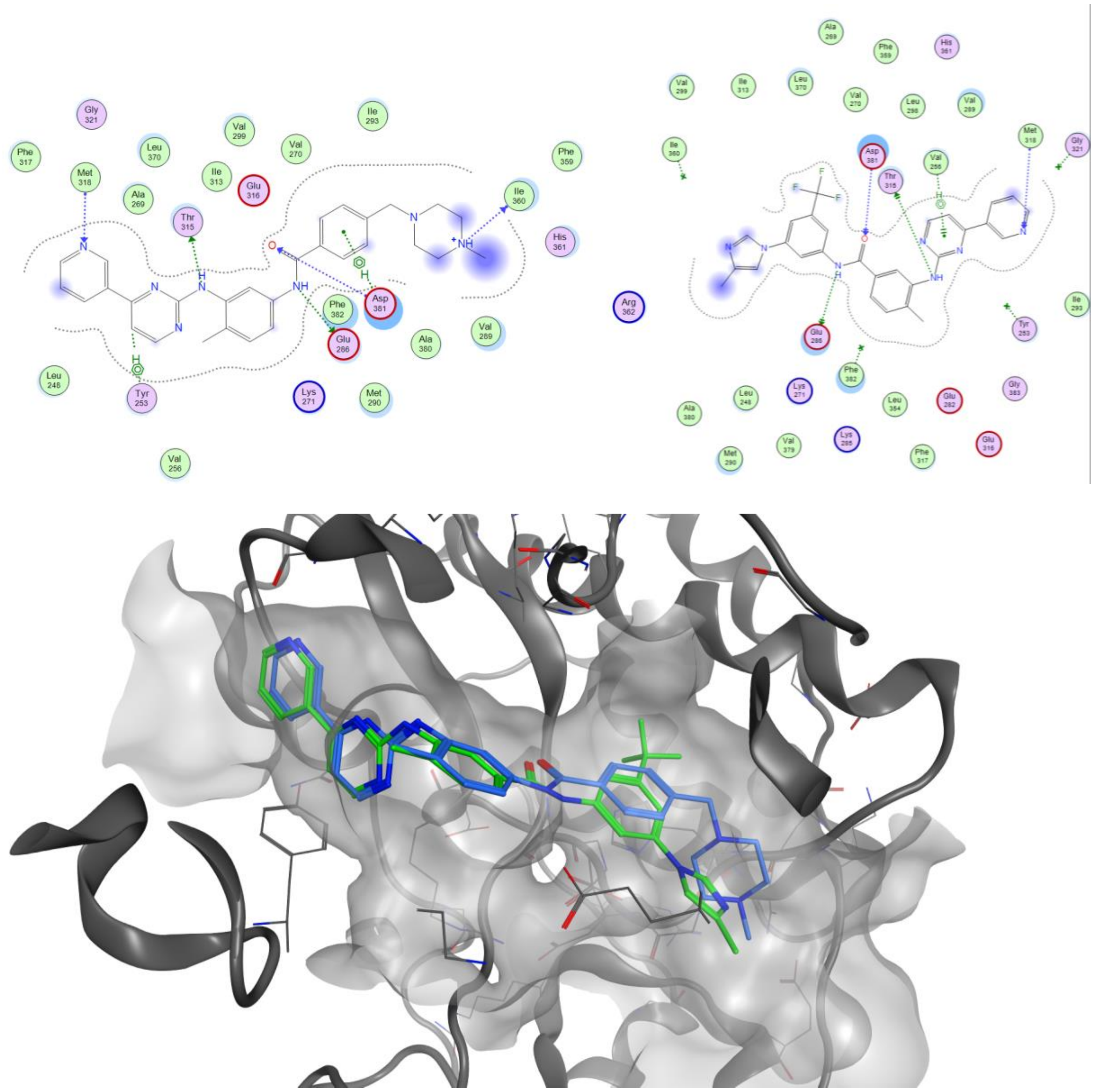

Figure S24. Up left) Imatinib 2D interaction with BCR-ABL kinase. Up right) Nilotinib 2D interactions with BCR-ABL kinase with BCR-ABL kinase. Down) Docked poses of imatinib (blue) and nilotinib (green) inside the binding pocket of BCR-ABL kinase (PDB ID: 1IEP).

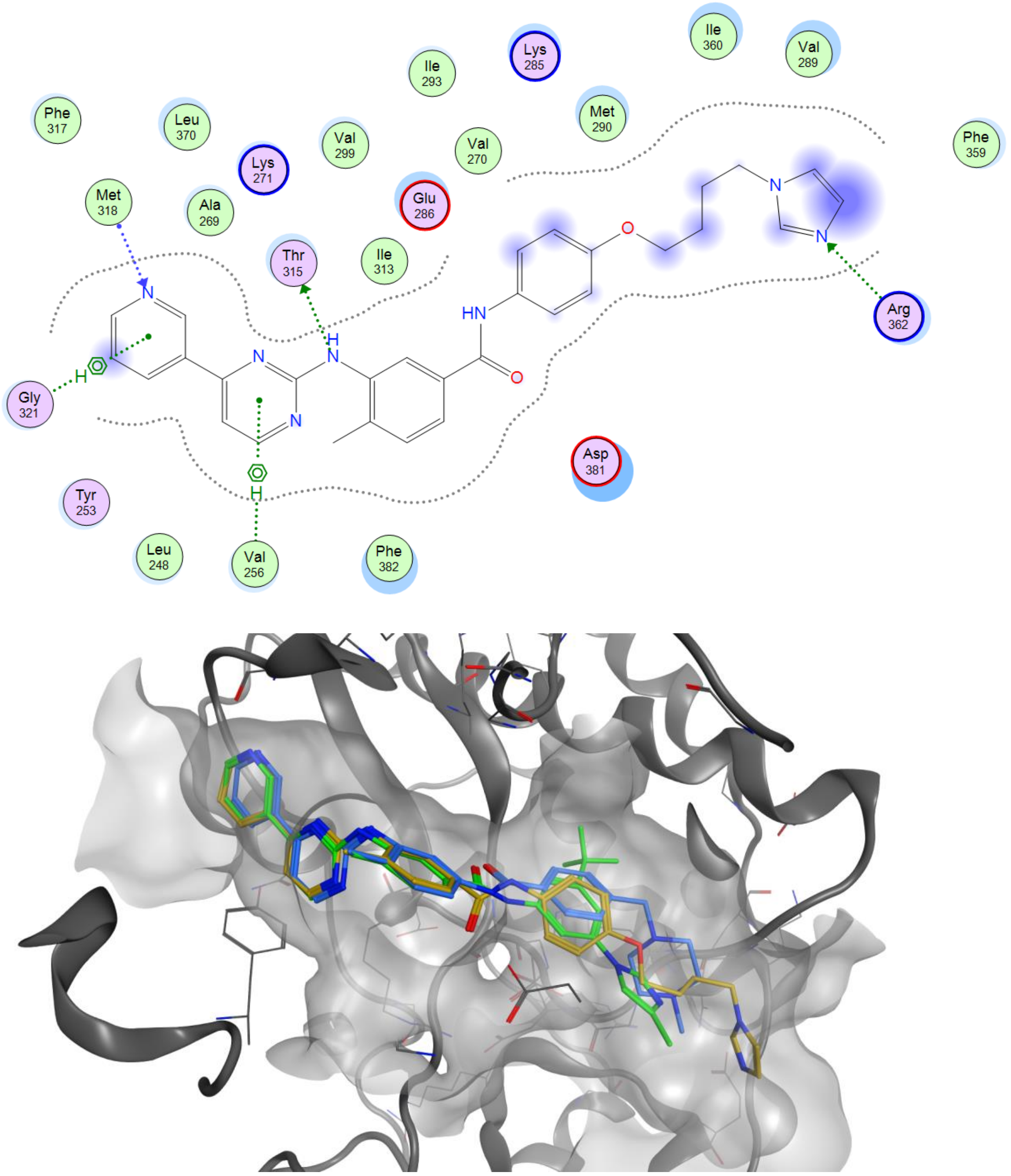

Figure S25. Up) **1a** 2D interaction with BCR-ABL kinase. Down) Docked poses of imatinib (blue), nilotinib (green) and **1a** (yellow) inside the binding pocket of BCR-ABL kinase (PDB ID: 1IEP).

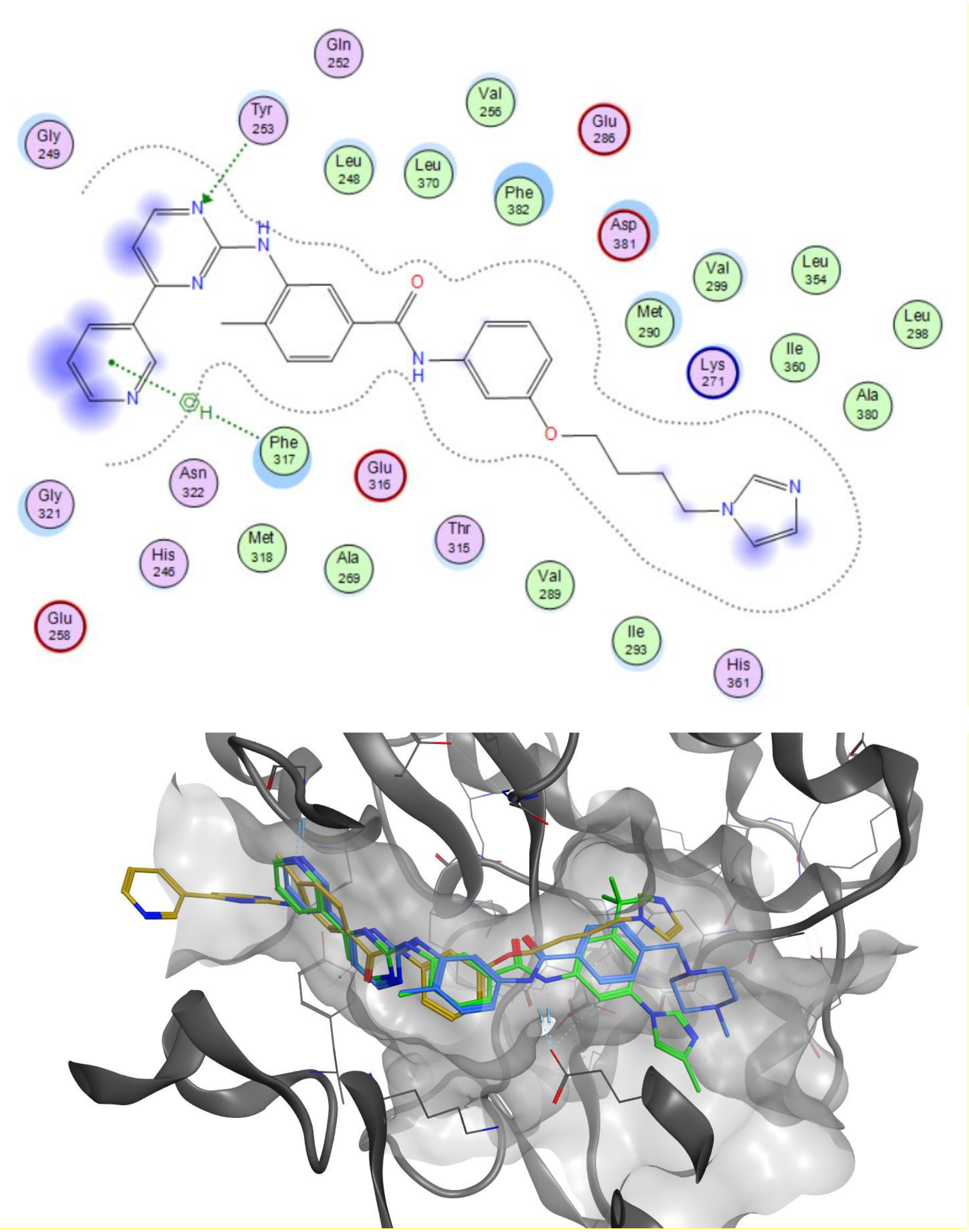

Figure S26. Up) **1b** 2D interaction with BCR-ABL kinase. Down) Docked poses of imatinib (blue), nilotinib (green) and **1b** (yellow) inside the binding pocket of BCR-ABL kinase (PDB ID: 1IEP).

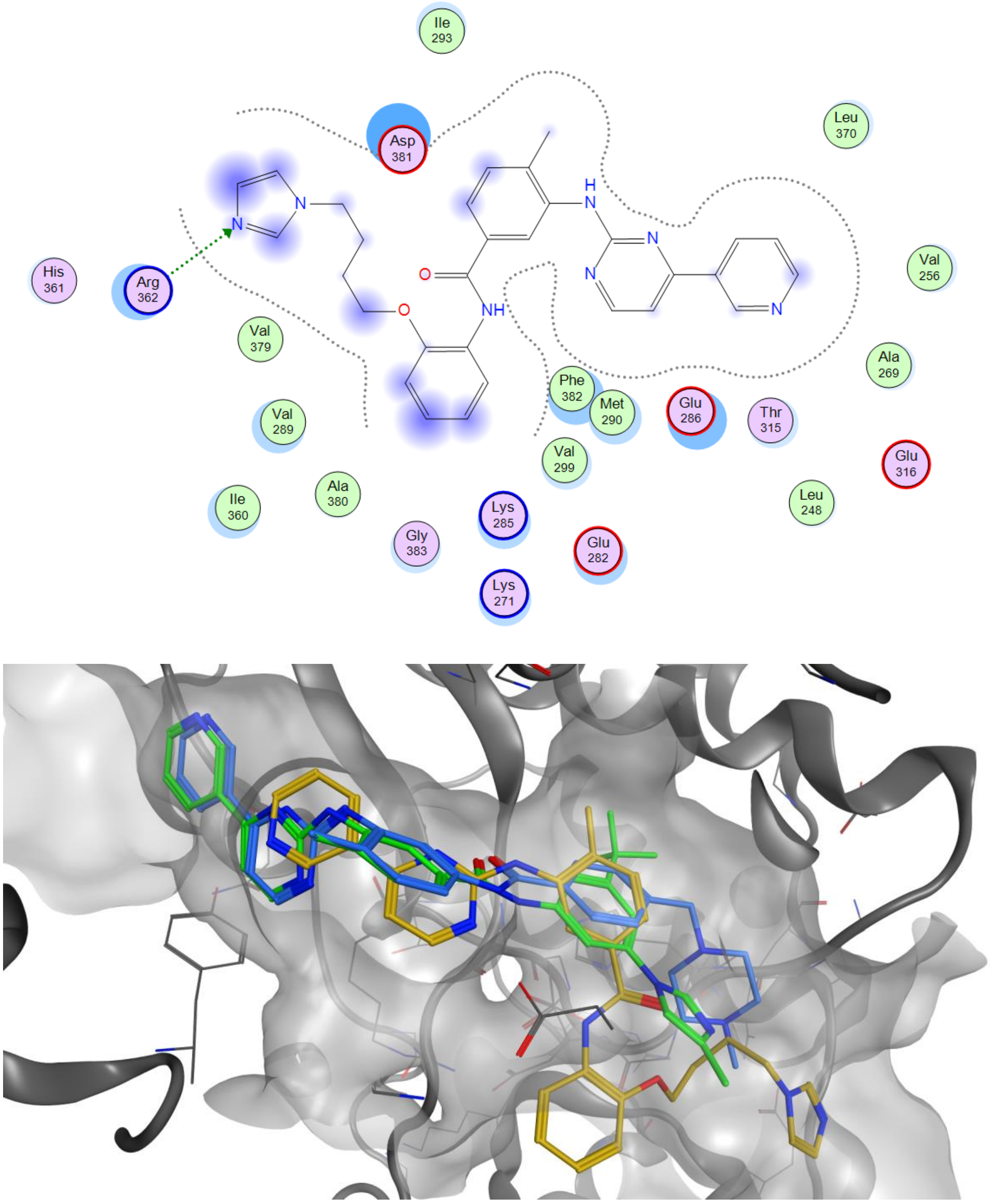

Figure S27. Up) **1c** 2D interaction with BCR-ABL kinase. Down) Docked poses of imatinib (blue), nilotinib (green) and **1c** (yellow) inside the binding pocket of BCR-ABL kinase (PDB ID: 1IEP).

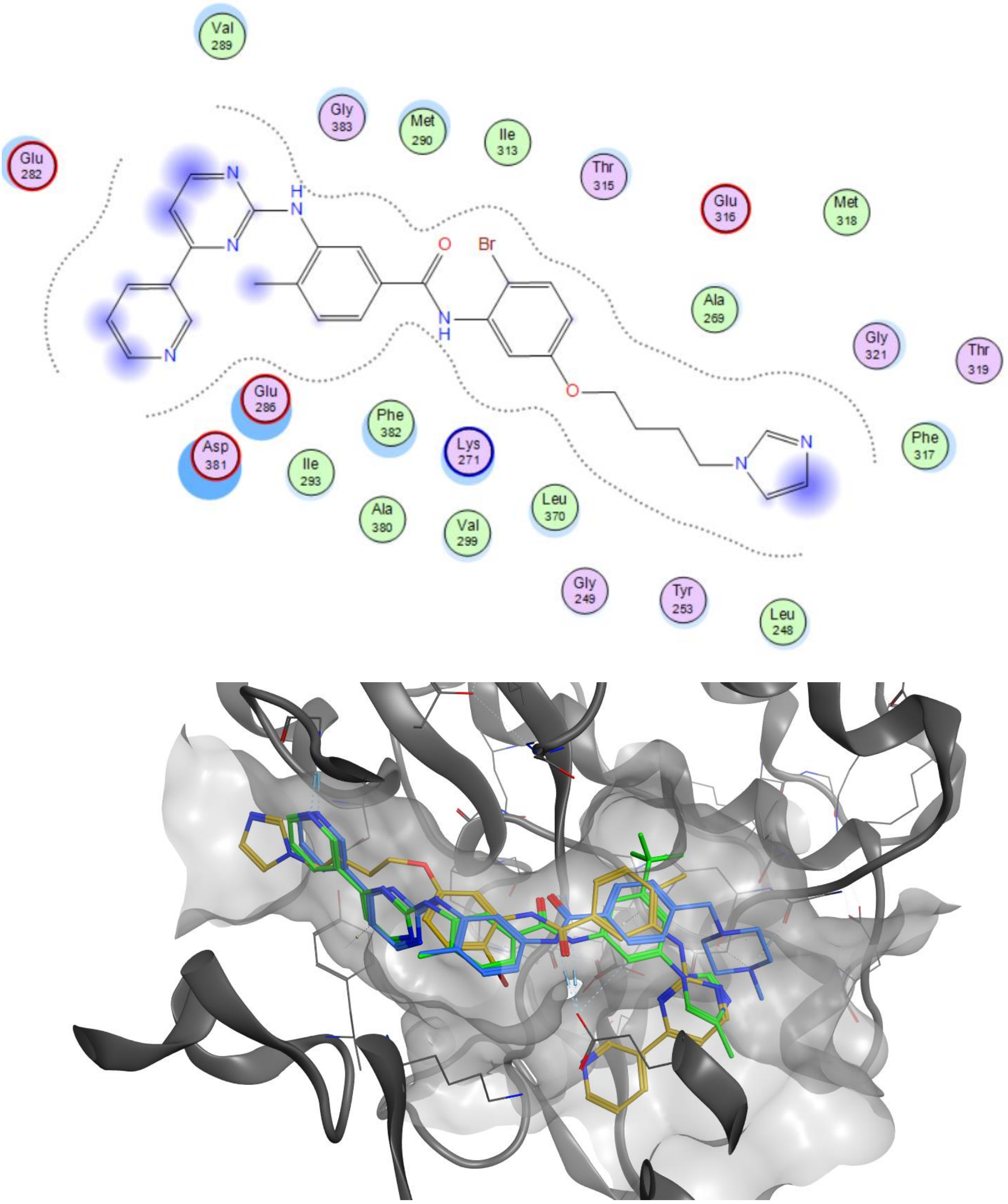

Figure S28. Up) **1d** 2D interaction with BCR-ABL kinase. Down) Docked poses of imatinib (blue), nilotinib (green) and **1d** (yellow) inside the binding pocket of BCR-ABL kinase (PDB ID: 1IEP).

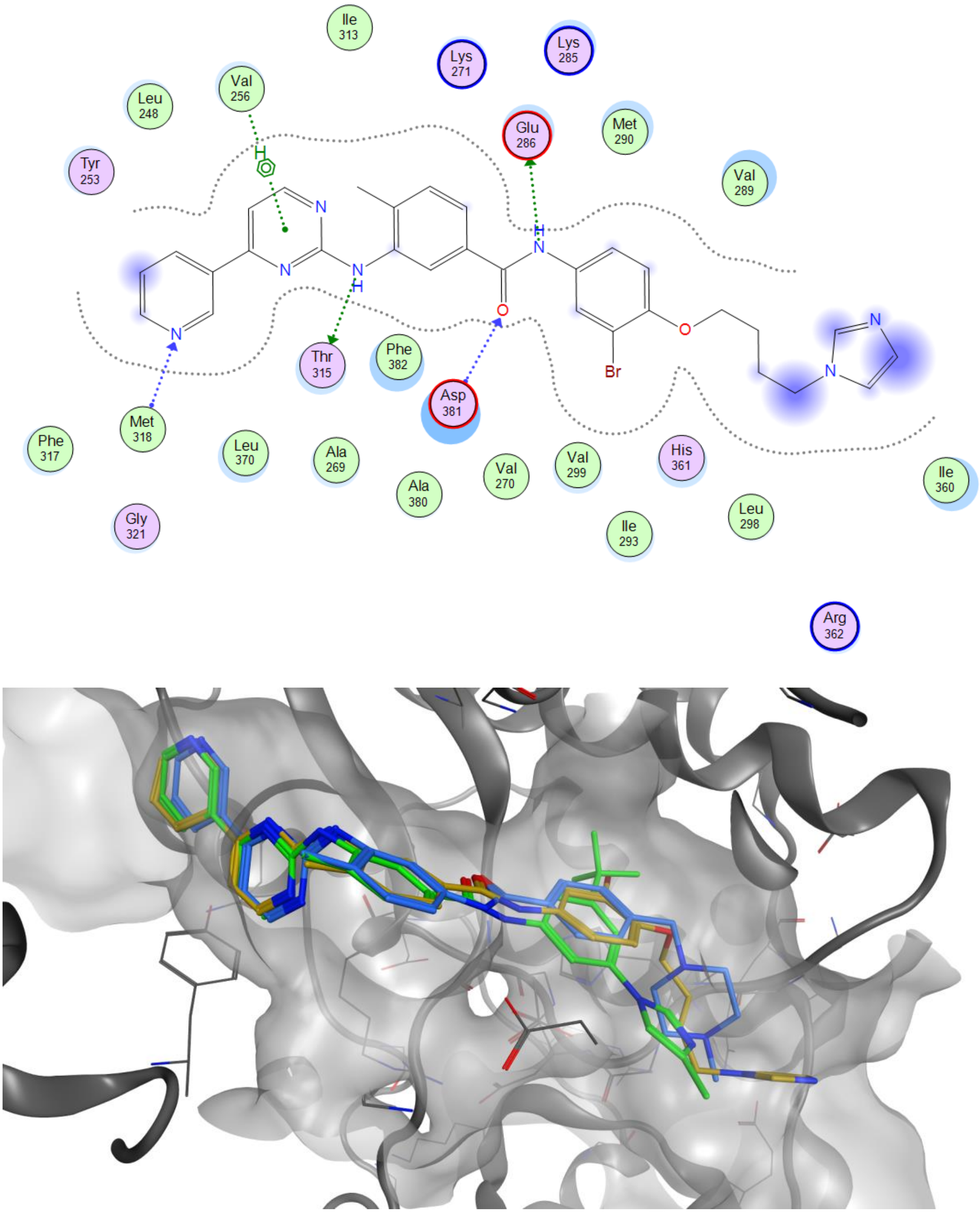

Figure S29. Up) **1e** 2D interaction with BCR-ABL kinase. Down) Docked poses of imatinib (blue), nilotinib (green) and **1e** (yellow) inside the binding pocket of BCR-ABL kinase (PDB ID: 1IEP).

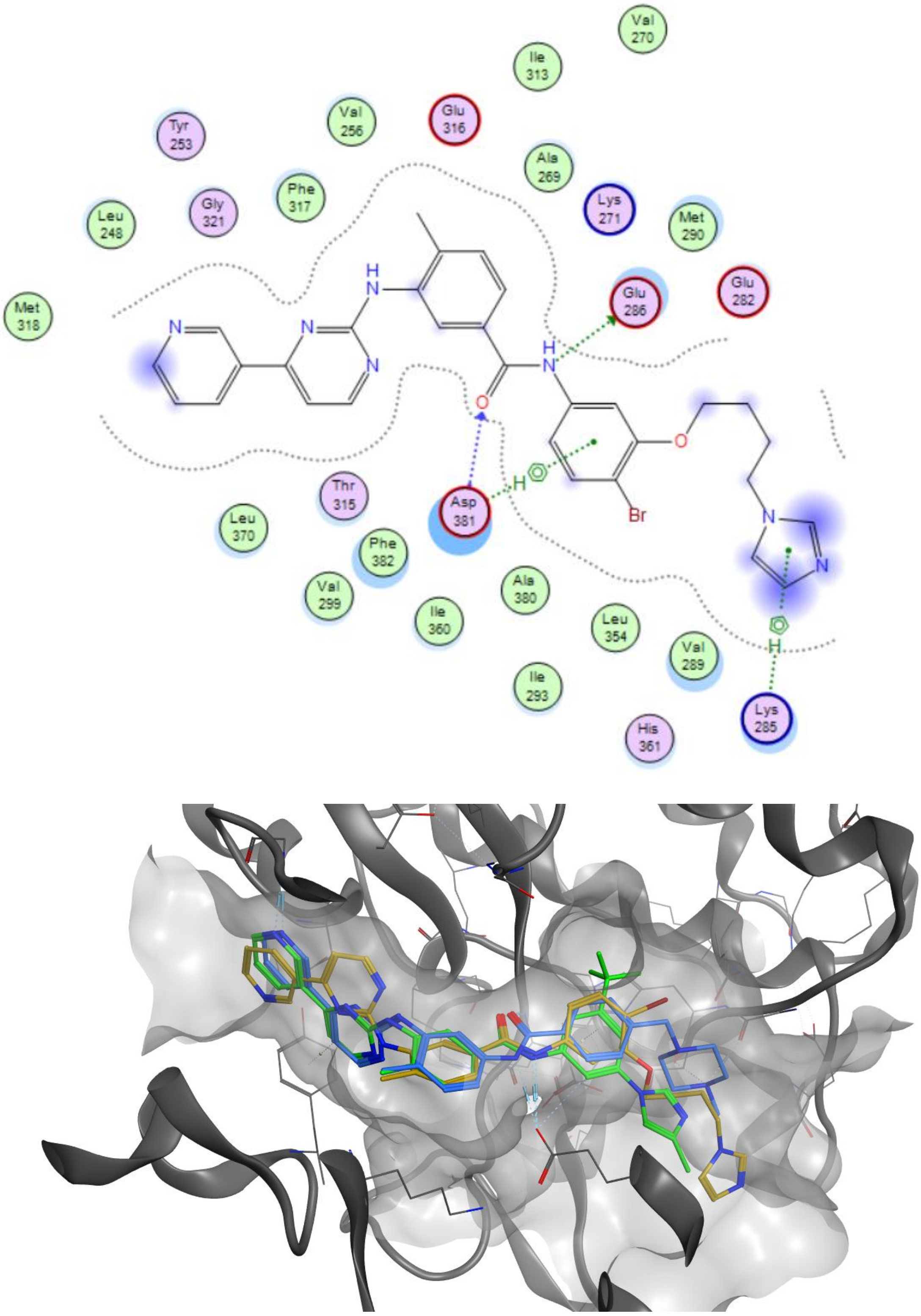

Figure S30. Up) **1f** 2D interaction with BCR-ABL kinase. Down) Docked poses of imatinib (blue), nilotinib (green), and **1f** (yellow) inside the binding pocket of BCR-ABL kinase (PDB ID: 1IEP). .

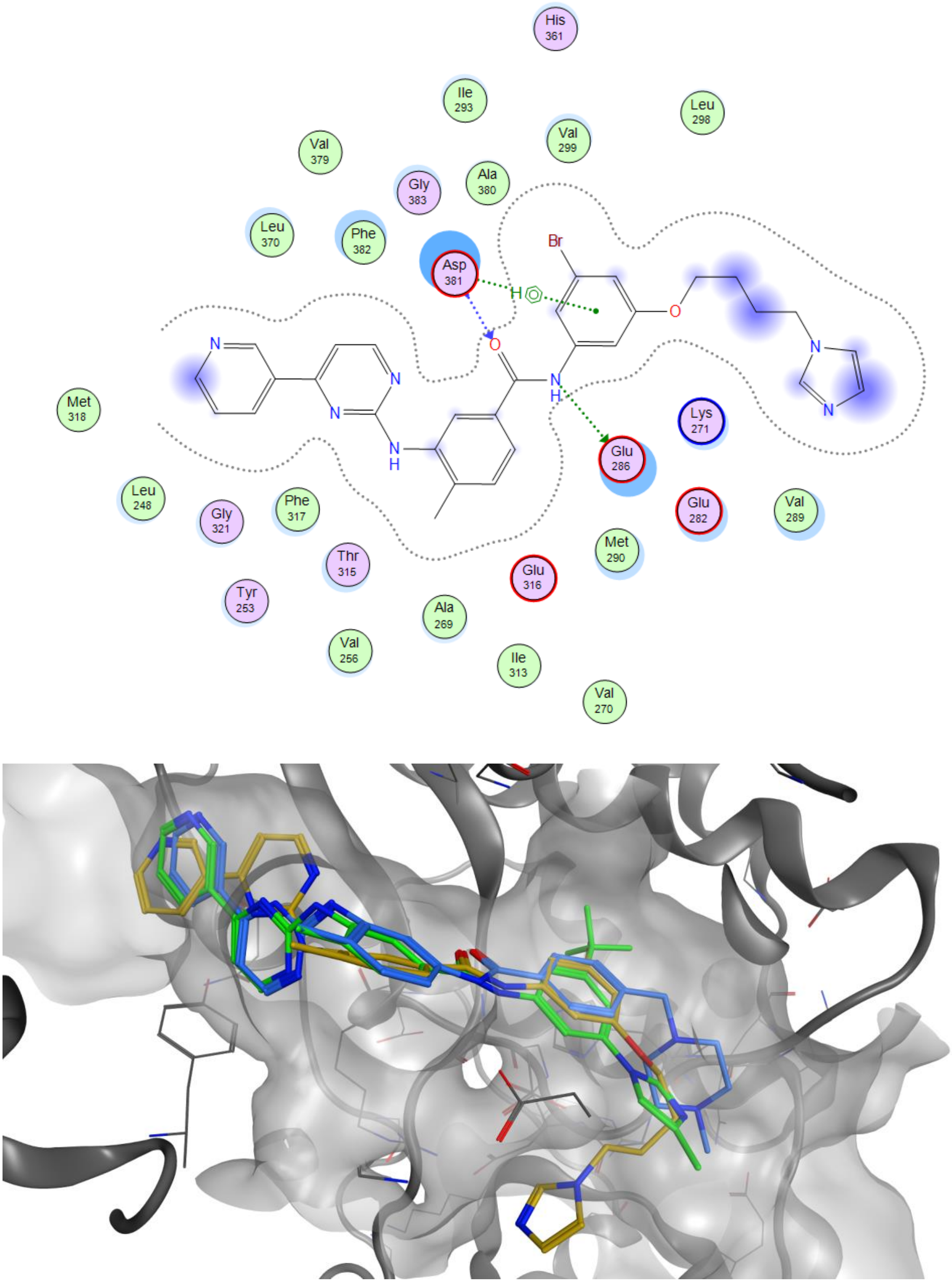

Figure S31. Up) **1g** 2D interaction withBCR-ABL kinase. Down) Docked poses of imatinib (blue), niltinib (green), and **1g** (yellow) inside the binding pocket of BCR-ABL kinase (PDB ID: 1IEP). .

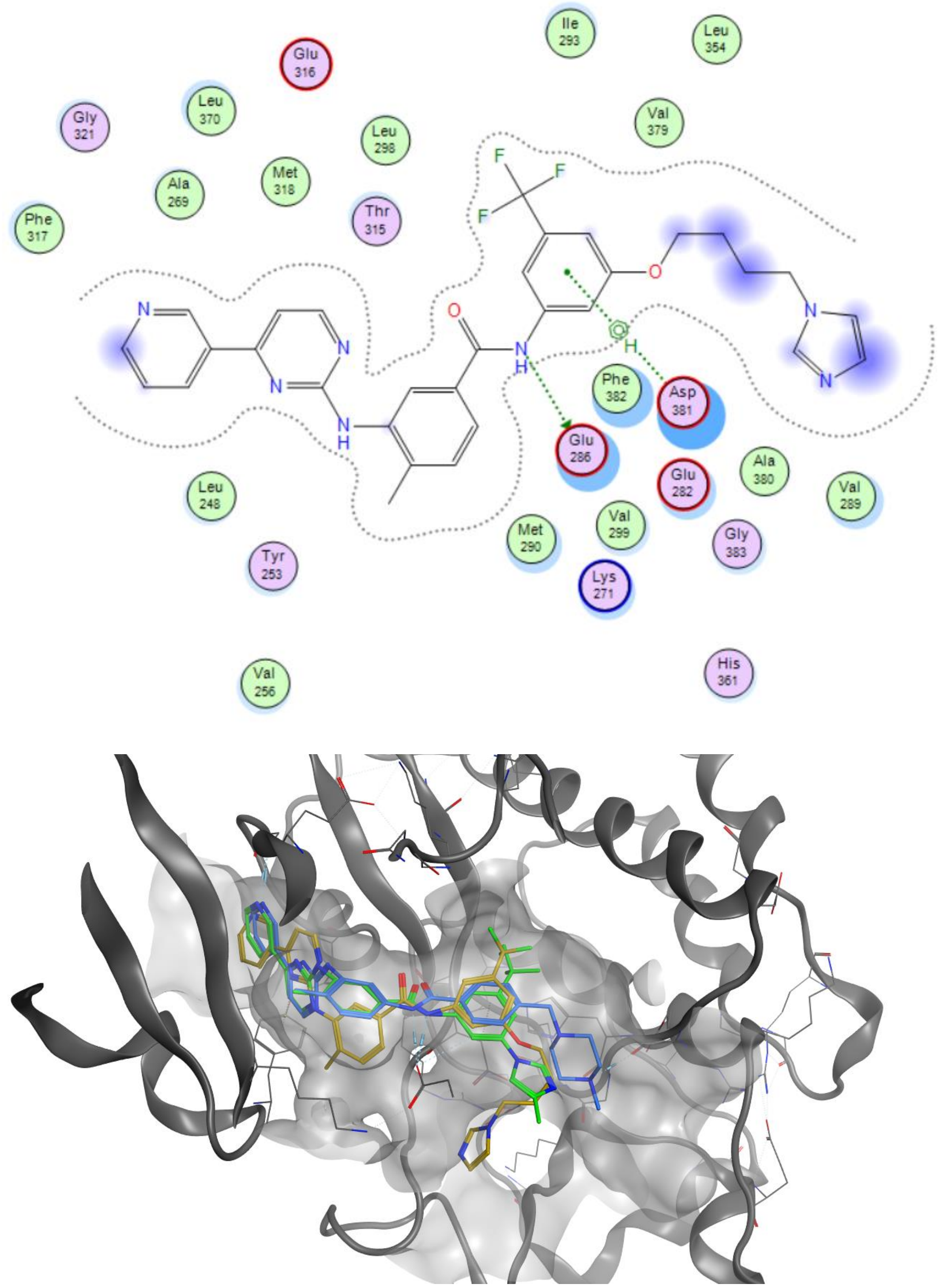

Figure S32. Up) **1h** 2D interaction withBCR-ABL kinase. Down) Docked poses of imatinib (blue), nilotinib (green), and **1h** (yellow) inside the binding pocket of BCR-ABL kinase (PDB ID: 1IEP).

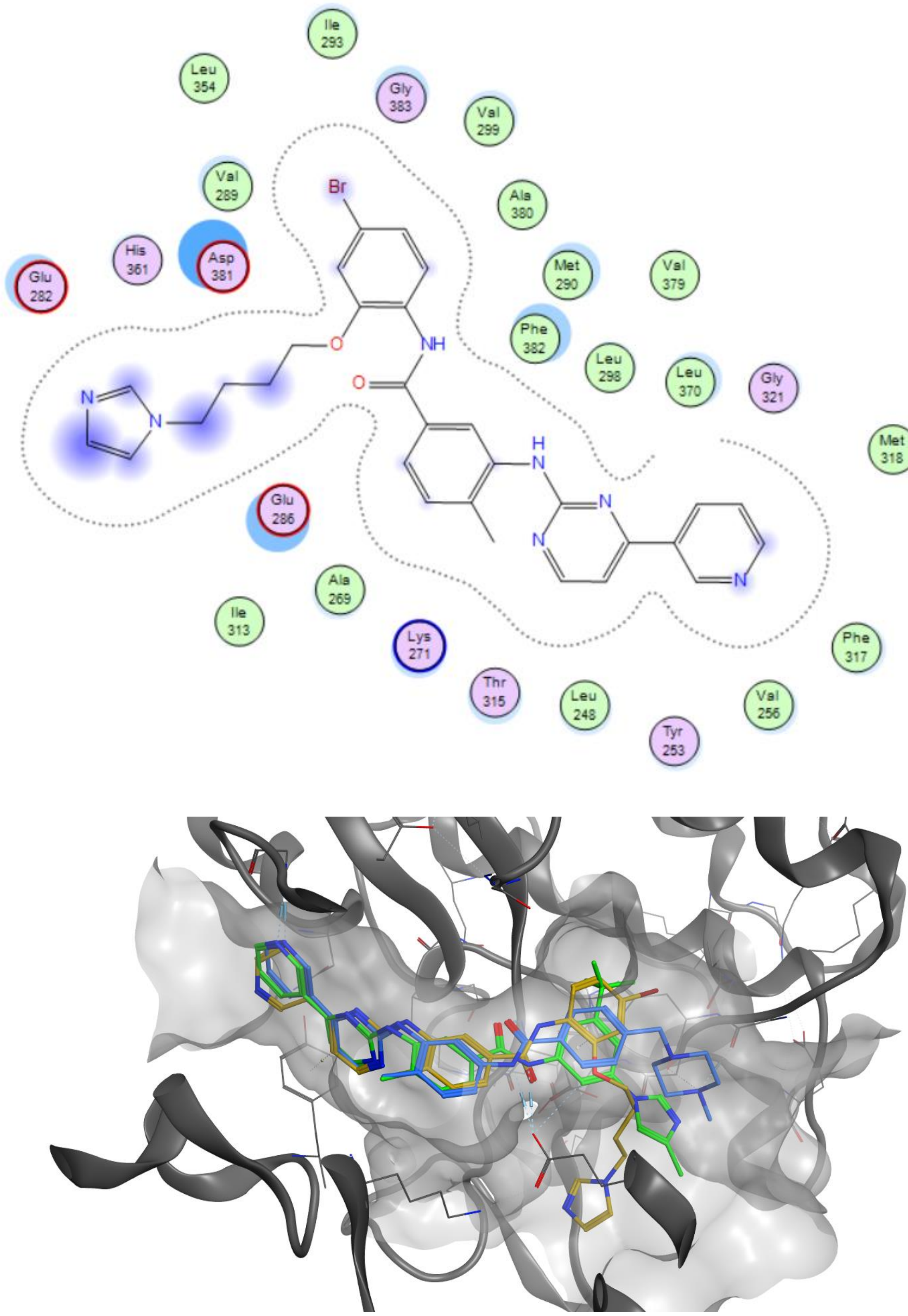

Figure S33. Up) **1i** 2D interaction with BCR-ABL kinase. Down) Docked poses of imatinib (blue), nilotinib (green), and **1i** (yellow) inside the binding pocket of BCR-ABL kinase (PDB ID: 1IEP).

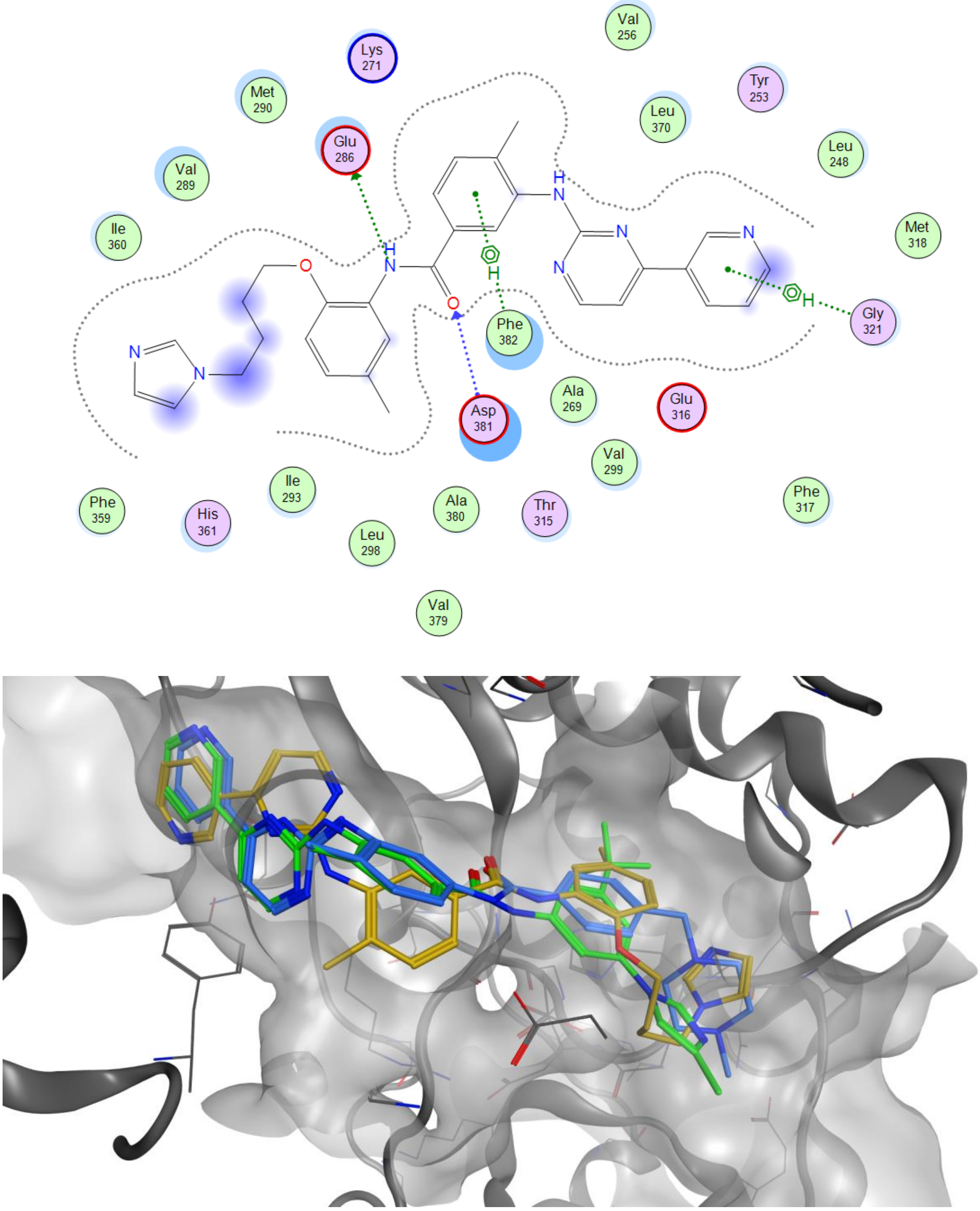

Figure S34. Up) **1j** 2D interaction with BCR-ABL kinase. Down) Docked poses of imatinib (blue), nilotinib (green) and **1j** (yellow) inside the binding pocket of BCR-ABL kinase (PDB ID: 1IEP).

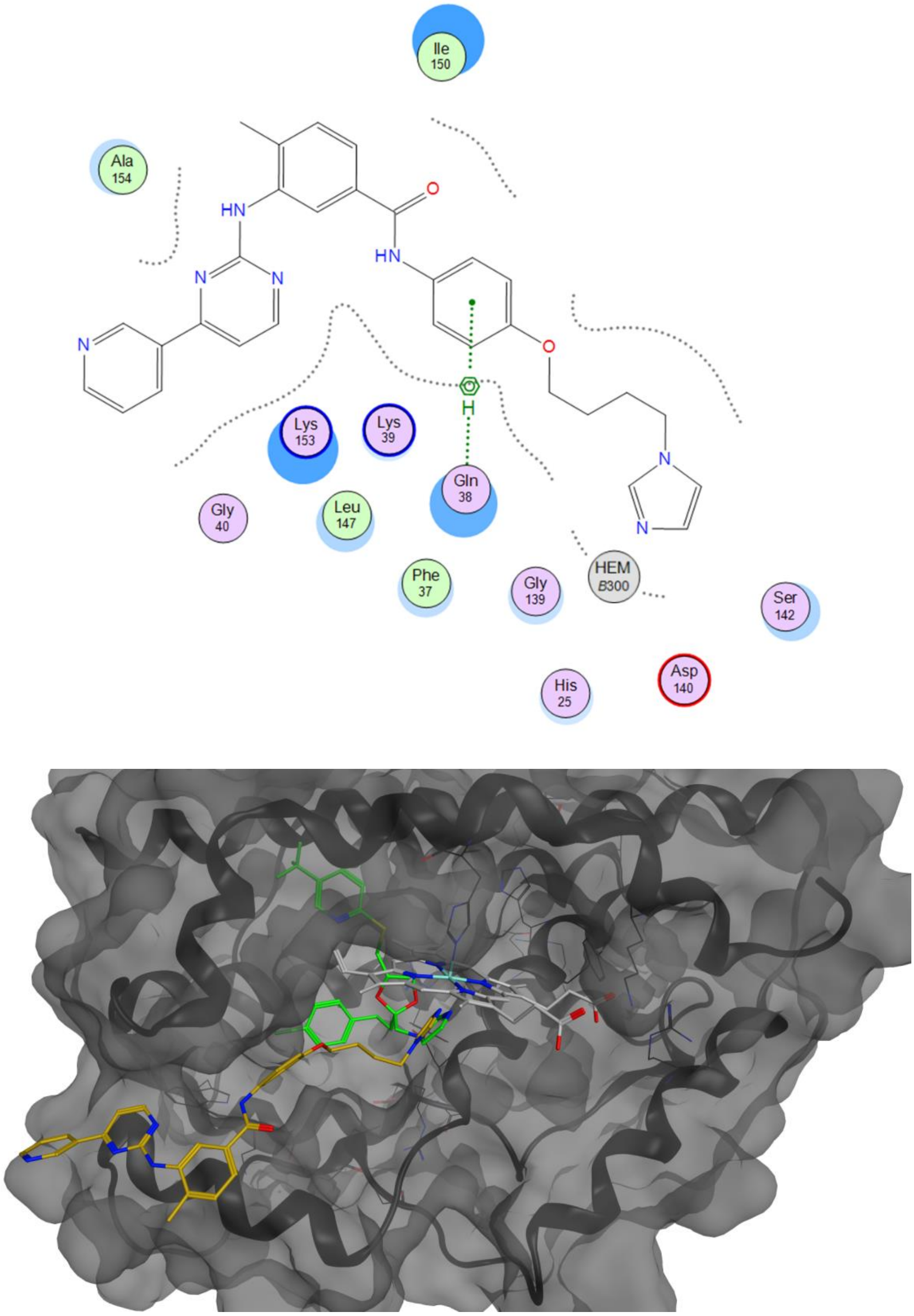

Figure S35. Up) **1a** 2D interaction with HO-1. Down) Docked poses of QC-80 (green) and **1a** (yellow) inside the binding pocket of HO-1 (PDB ID: 3HOK).

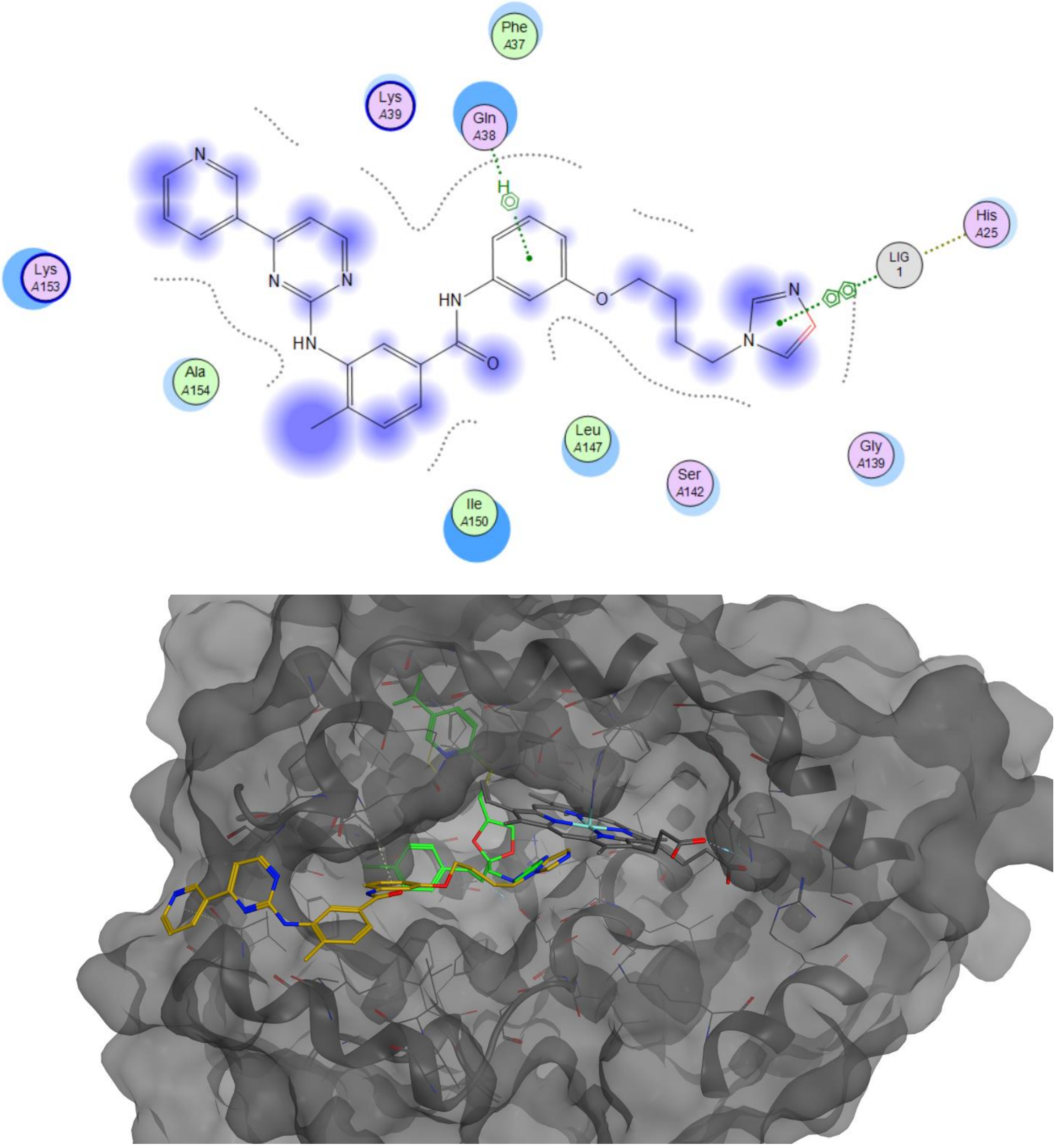

Figure S36. Up) **1b** 2D interaction with HO-1. Down) Docked poses of QC-80 (green) and **1b** (yellow) inside the binding pocket of HO-1 (PDB ID: 3HOK).

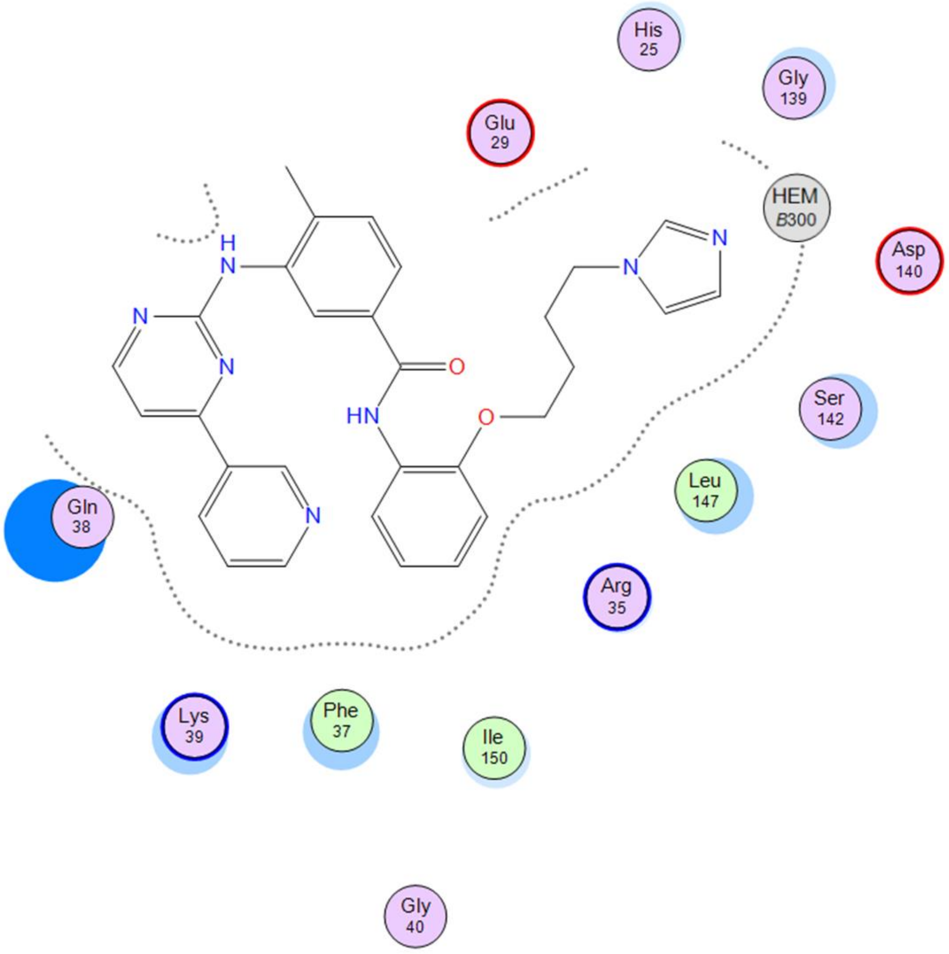

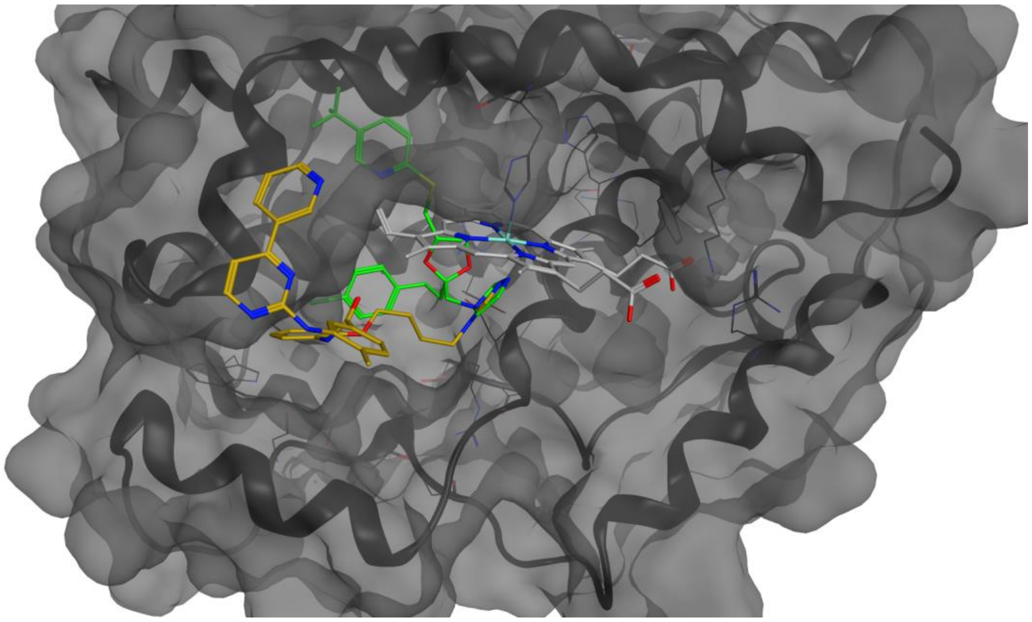

Figure S37. Up) **1c** 2D interaction with HO-1. Down) Docked poses of QC-80 (green) and **1c** (yellow) inside the binding pocket of HO-1 (PDB ID: 3HOK).

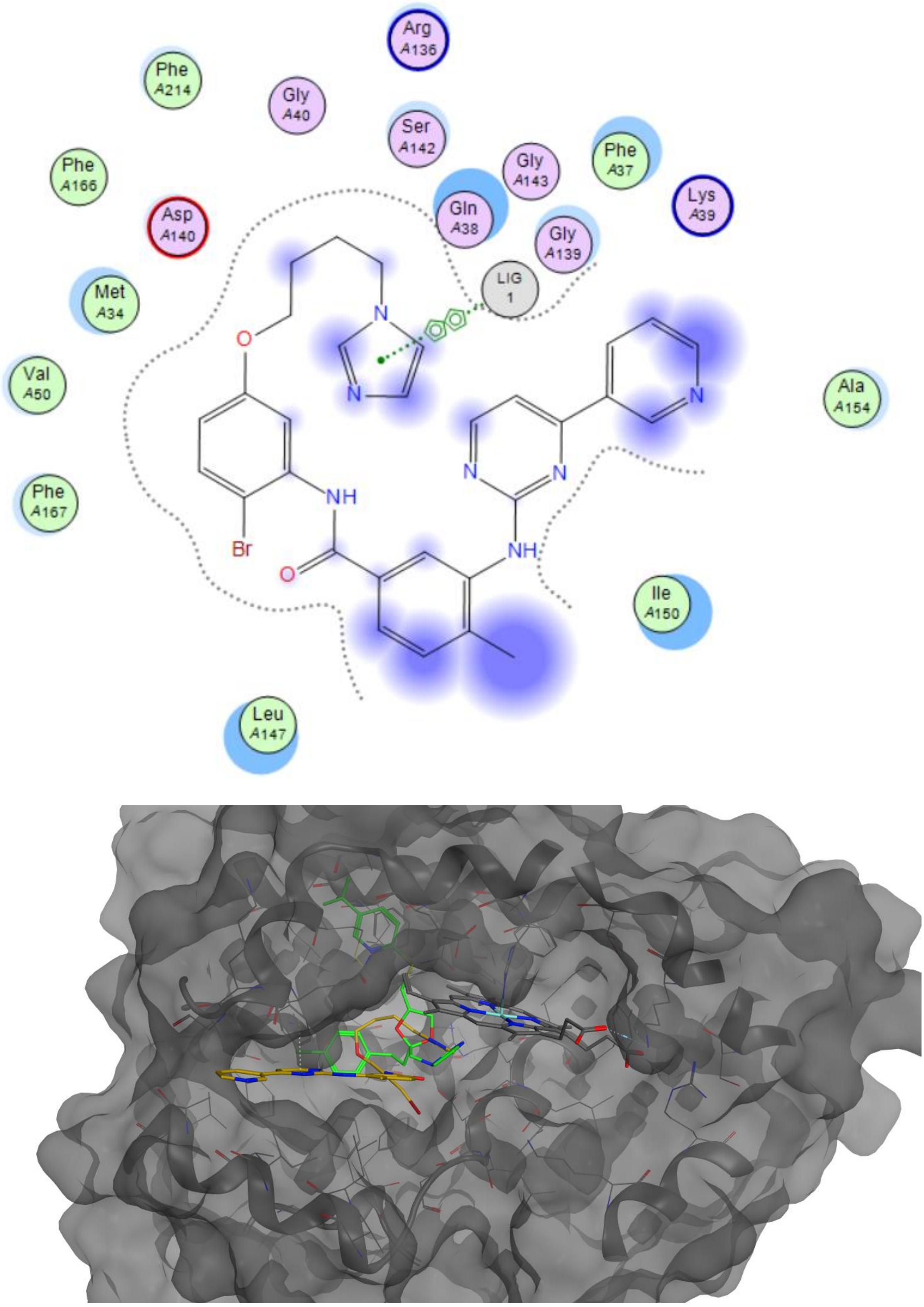

Figure S38. Up) **1d** 2D interaction with HO-1. Down) Docked poses of QC-80 (green) and **1d** (yellow) inside the binding pocket of HO-1 (PDB ID: 3HOK).

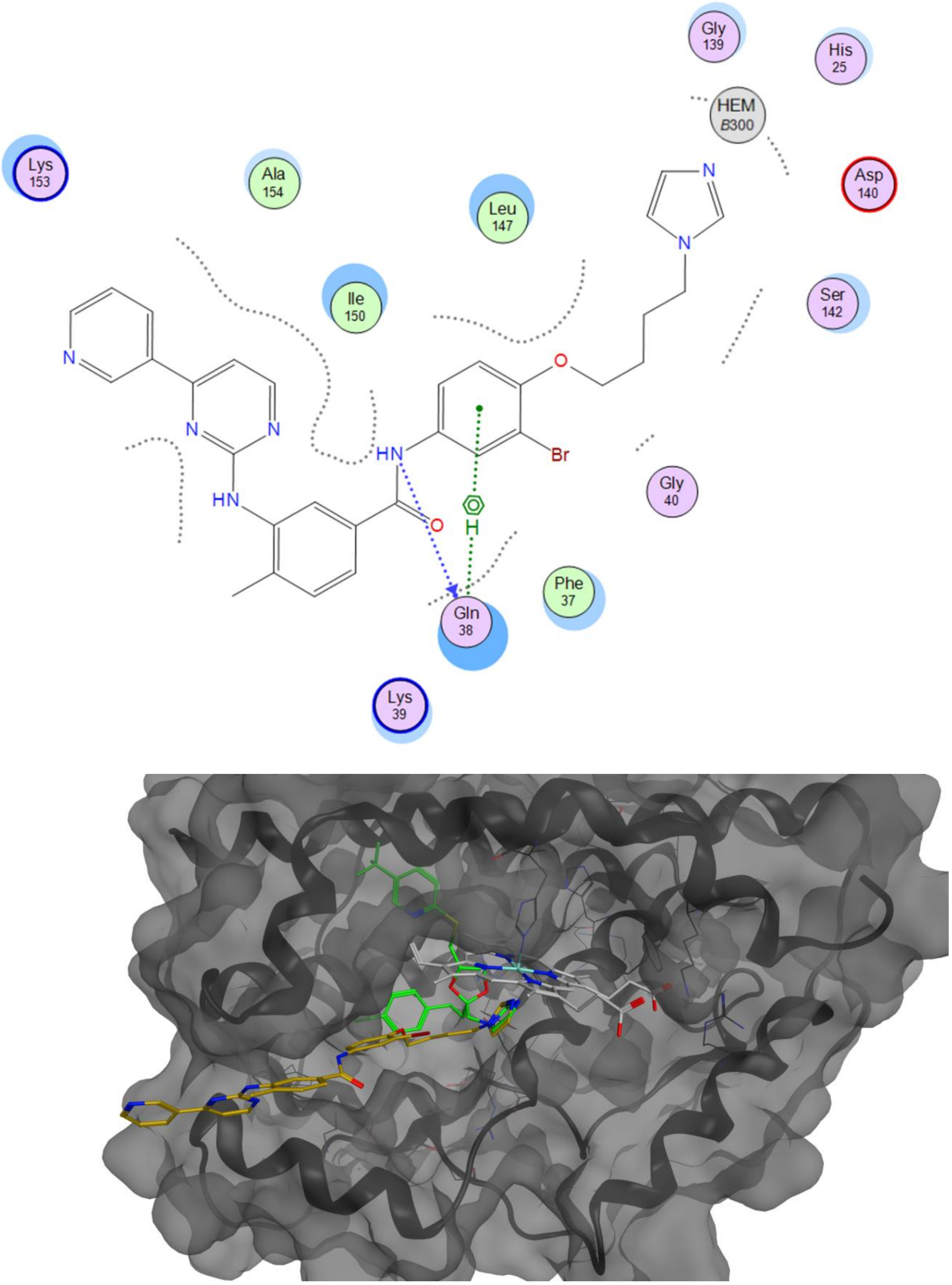

Figure S39. Up) **1e** 2D interaction with HO-1. Down) Docked poses of QC-80 (green) and **1e** (yellow) inside the binding pocket of HO-1 (PDB ID: 3HOK).

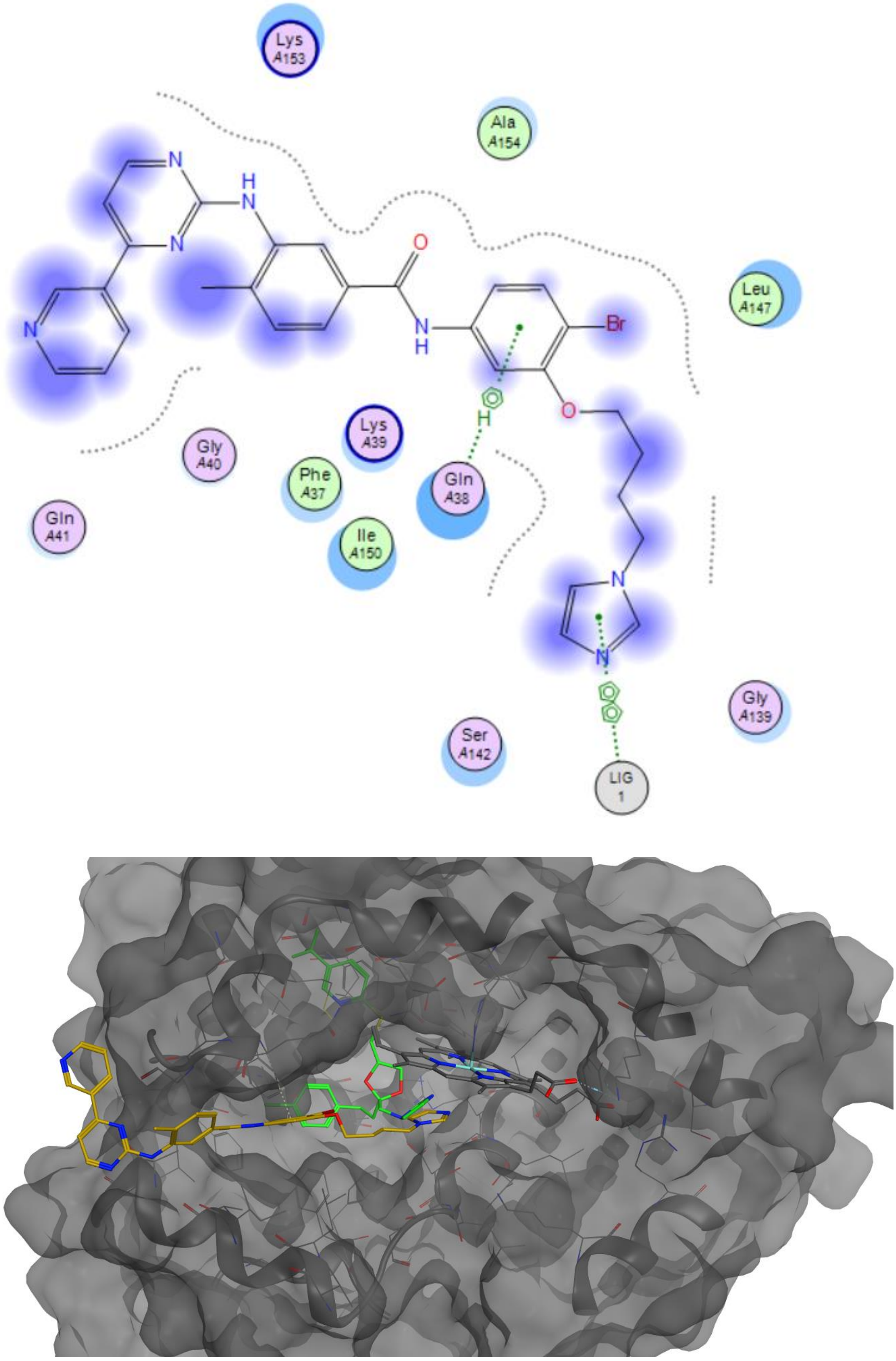

Figure S40. Up) **1f** 2D interaction with HO-1. Down) Docked poses of QC-80 (green) and **1f** (yellow) inside the binding pocket of HO-1 (PDB ID: 3HOK).

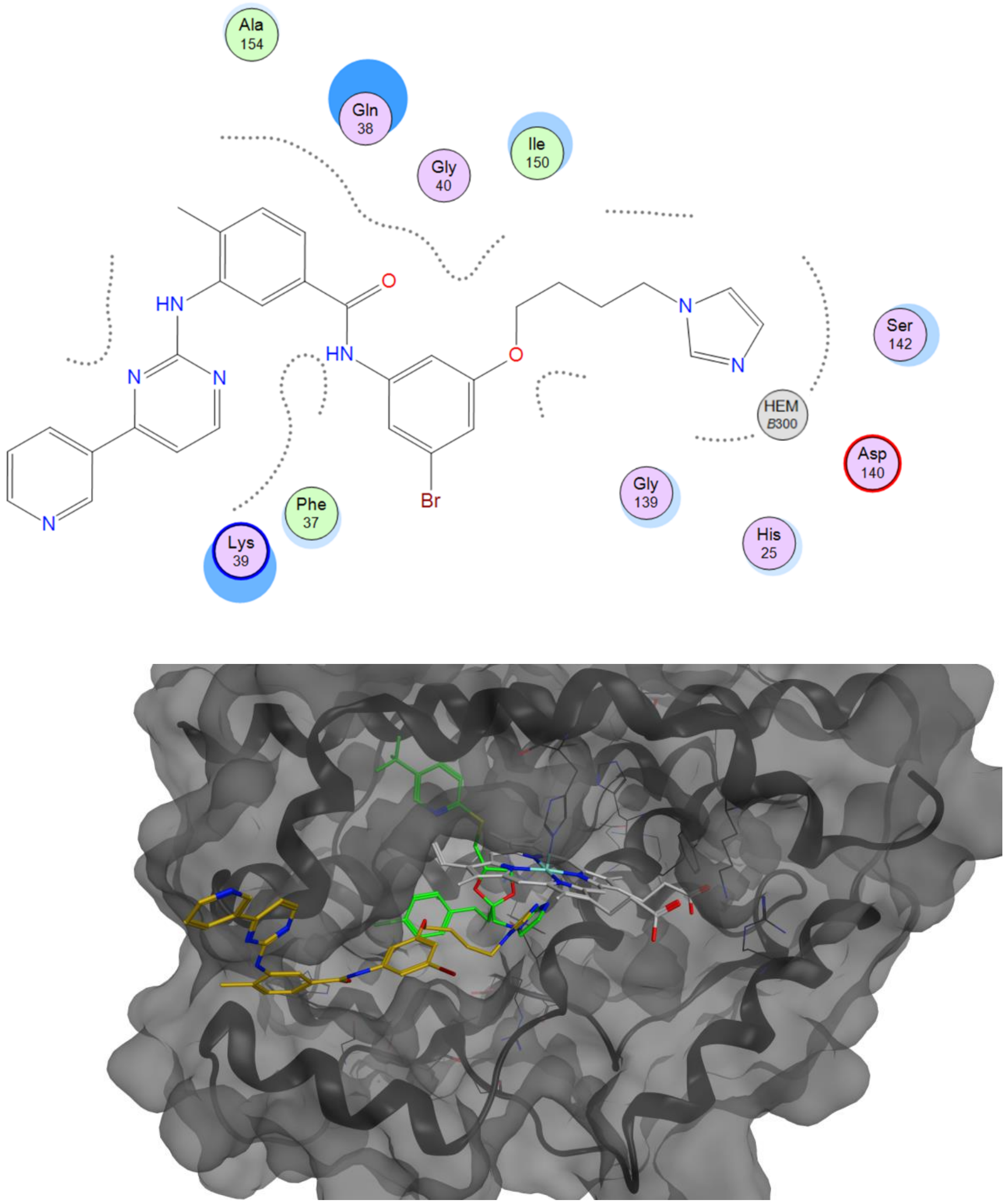

Figure S41. Up) **1g** 2D interaction with HO-1. Down) Docked poses of QC-80 (green) and **1g** (yellow) inside the binding pocket of HO-1 (PDB ID: 3HOK).

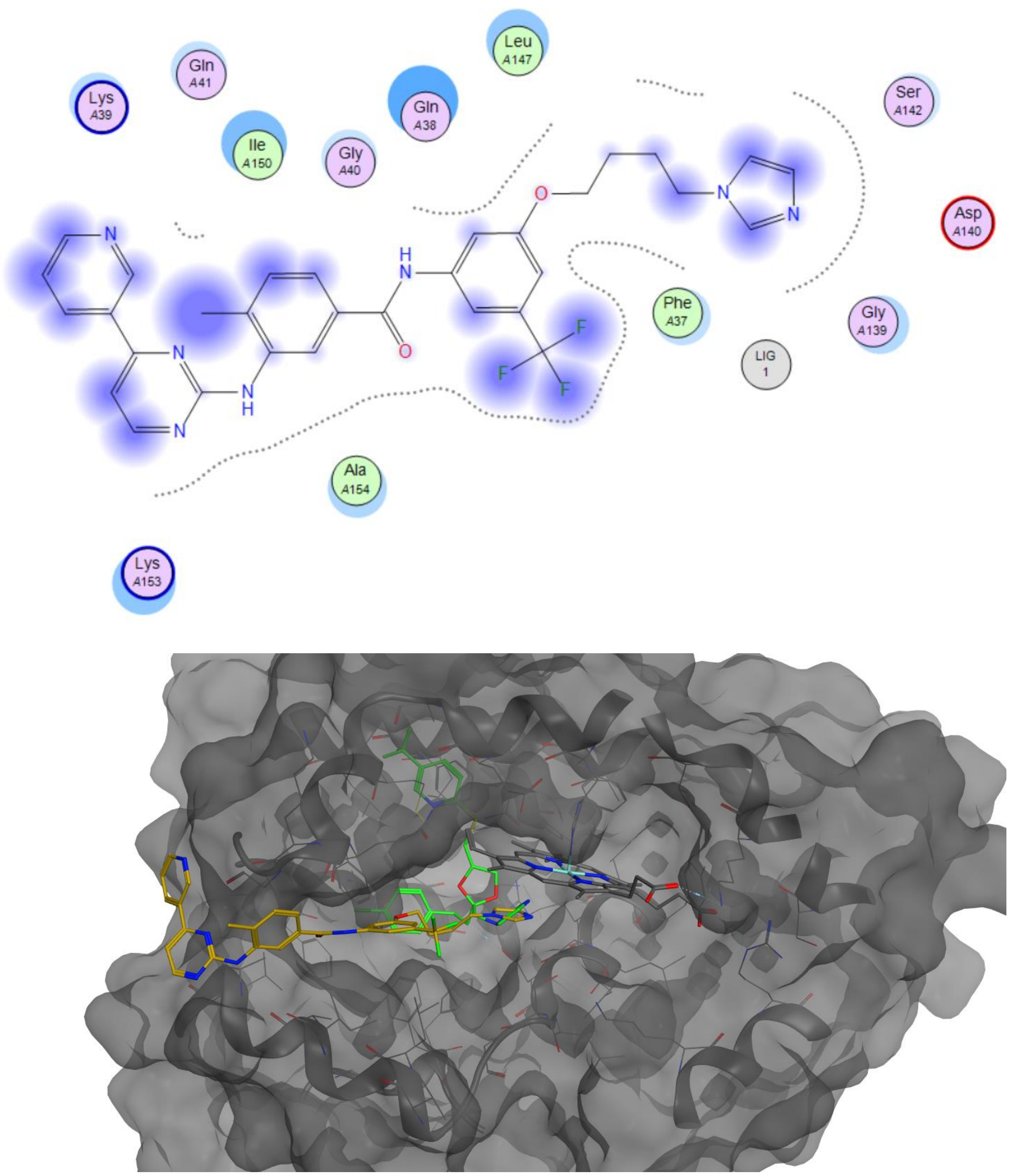

Figure S42. Up) **1h** 2D interaction with HO-1. Down) Docked poses of QC-80 (green) and **1h** (yellow) inside the binding pocket of HO-1 (PDB ID: 3HOK).

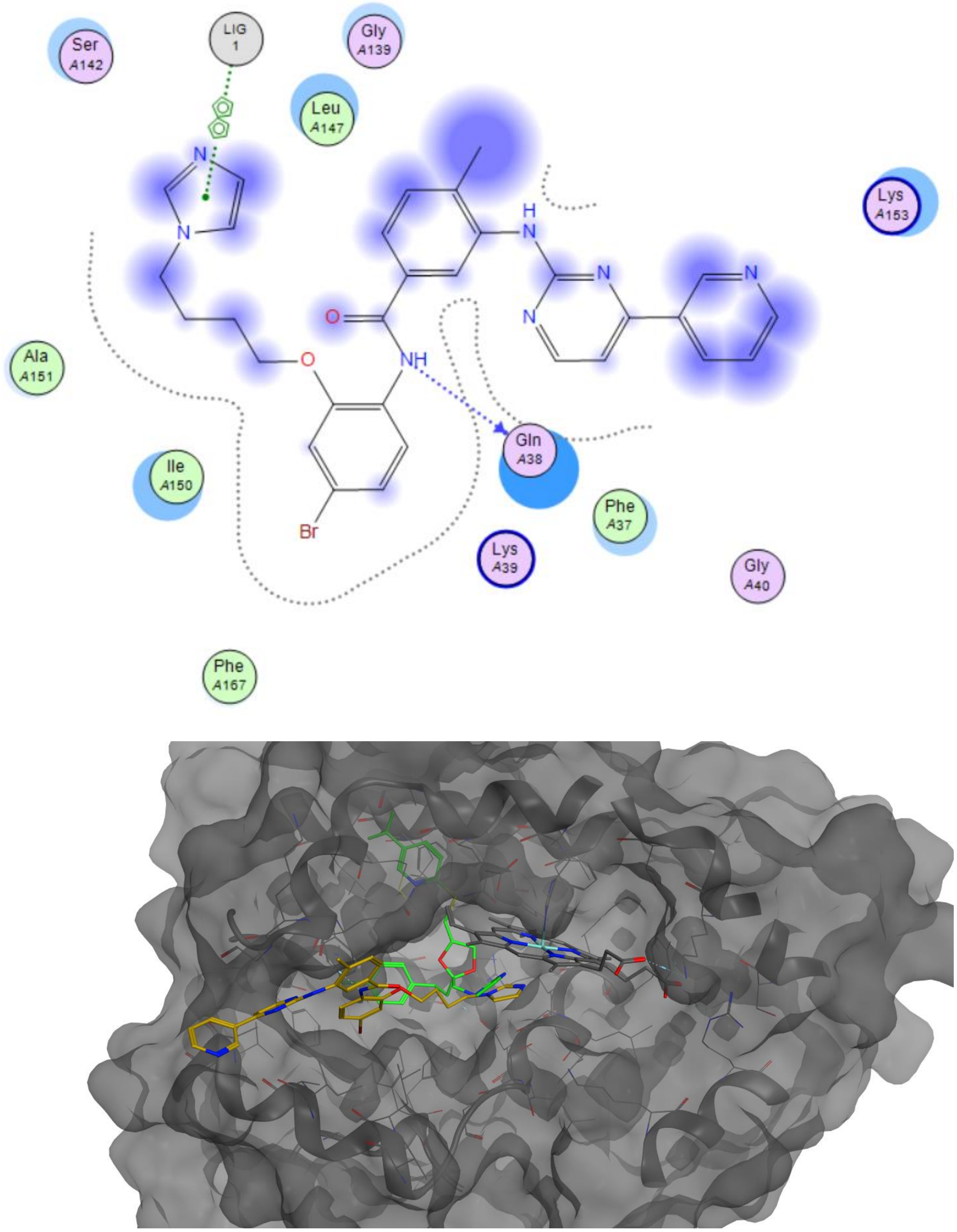

Figure S43. Up) **1i** 2D interaction with HO-1. Down) Docked poses of QC-80 (green) and **1i** (yellow) inside the binding pocket of HO-1 (PDB ID: 3HOK).

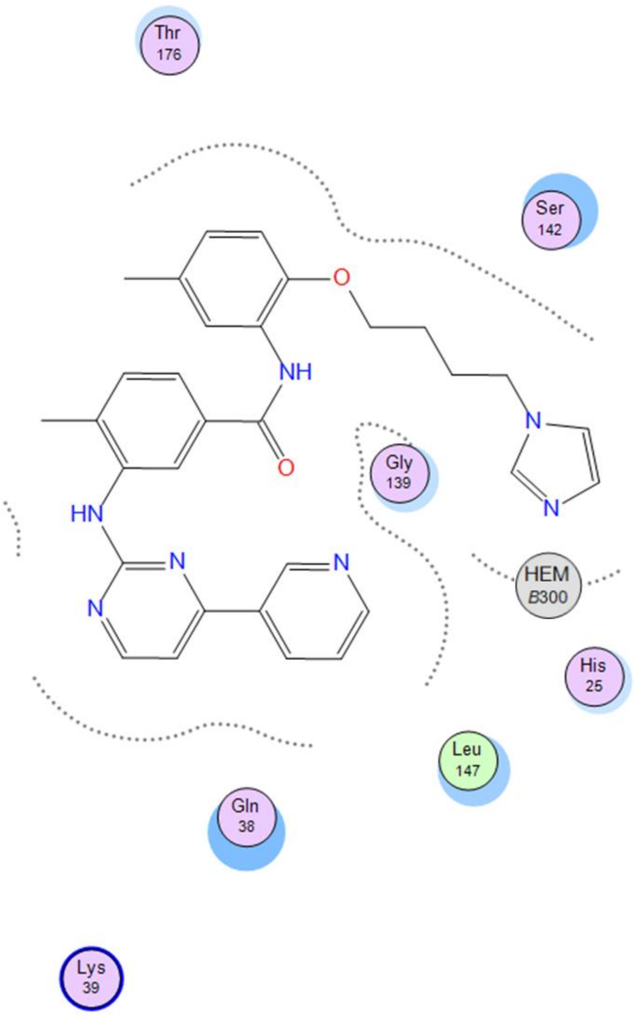

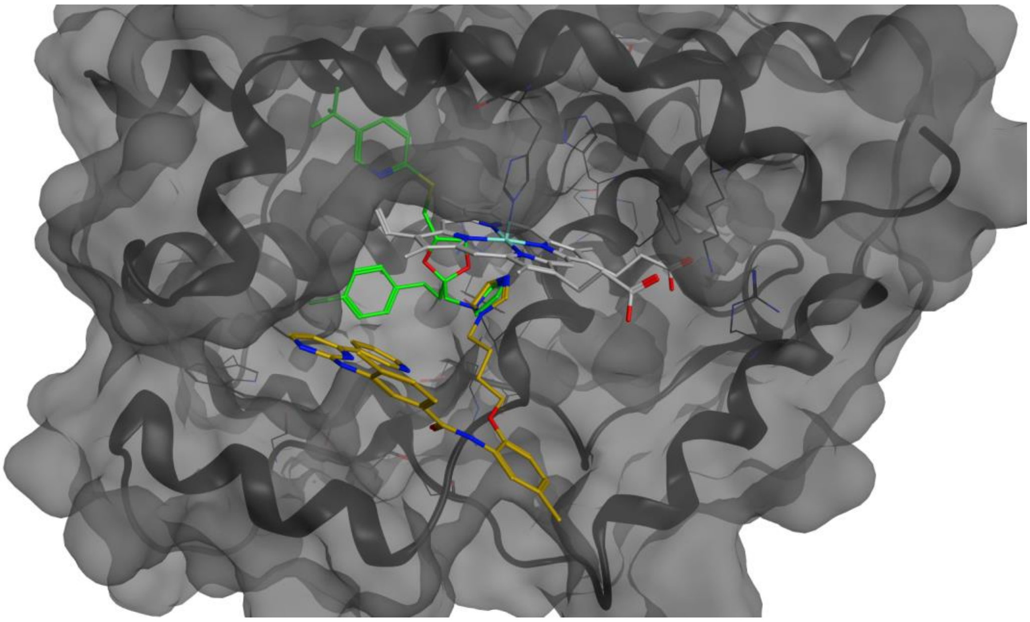

Figure S44. Up) **1j** 2D interaction with HO-1. Down) Docked poses of QC-80 (green) and **1j** (yellow) inside the binding pocket of HO-1 (PDB ID: 3HOK).
